# A novel class of sulphonamides potently block malaria transmission by targeting a *Plasmodium* vacuole membrane protein

**DOI:** 10.1242/dmm.049950

**Published:** 2023-01-30

**Authors:** Sabrina Yahiya, Charlie N. Saunders, Sarah Hassan, Ursula Straschil, Oliver J. Fischer, Ainoa Rueda-Zubiaurre, Silvia Haase, Gema Vizcay-Barrena, Mufuliat Toyin Famodimu, Sarah Jordan, Michael J. Delves, Edward W. Tate, Anna Barnard, Matthew J. Fuchter, Jake Baum

**Affiliations:** ^1^ Department of Life Sciences, Imperial College London, Sir Alexander Fleming Building, Exhibition Road, South Kensington, London SW7 2AZ, UK; ^2^ Department of Chemistry, Imperial College London, Molecular Sciences Research Hub, White City Campus, Wood Lane, London W12 OBZ, UK; ^3^ Centre for Ultrastructural Imaging, New Hunt's House, Guy's Campus, King's College London, London SE1 1UL, UK

**Keywords:** *Plasmodium falciparum*, Malaria, High-throughput screening, Target identification, Target validation, Transmission, Drug discovery, Exflagellation, Gametocytes, Gametogenesis

## Abstract

Phenotypic cell-based screens are critical tools for discovering candidate drugs for development, yet identification of the cellular target and mode of action of a candidate drug is often lacking. Using an imaging-based screen, we recently discovered an *N-*[(4-hydroxychroman-4-yl)methyl]-sulphonamide (*N-*4HCS) compound, DDD01035881, that blocks male gamete formation in the malaria parasite life cycle and subsequent transmission of the parasite to the mosquito with nanomolar activity. To identify the target(s) of DDD01035881, and of the *N*-4HCS class of compounds more broadly, we synthesised a photoactivatable derivative, probe 2. Photoaffinity labelling of probe 2 coupled with mass spectrometry identified the 16 kDa *Plasmodium falciparum* parasitophorous vacuole membrane protein Pfs16 as a potential parasite target. Complementary methods including cellular thermal shift assays confirmed that the parent molecule DDD01035881 stabilised Pfs16 in lysates from activated mature gametocytes. Combined with high-resolution, fluorescence and electron microscopy data, which demonstrated that parasites inhibited with *N-*4HCS compounds phenocopy the targeted deletion of *Pfs16* in gametocytes, these data implicate Pfs16 as a likely target of DDD01035881. This finding establishes *N-*4HCS compounds as being flexible and effective starting candidates from which transmission-blocking antimalarials can be developed in the future.

## Teaser

**Editor's choice:** A new class of antimalarials based on a *N-*[(4-hydroxychroman-4-yl)methyl]-sulphonamide scaffold potently prevents malaria parasite transmission to the mosquito by targeting the *Plasmodium falciparum* vacuole membrane protein Pfs16.

## INTRODUCTION

Malaria continues to devastate millions of people, with 247 million cases and 619,000 deaths from malaria reported in 2021 alone (World Health Organization, 2022). The causative agent of malaria, the *Plasmodium* parasite, transitions between a mammalian host and *Anopheles* mosquito vector, demonstrating extensive cellular plasticity in form across the different life cycle stages (Prudêncio et al., 2006). Symptomatic malaria disease is restricted to the asexual blood stages and can be targeted by current frontline antimalarials, including artemisinin and its derivatives (Wells et al., 2015). Sexual forms (male and female gametocytes) are, however, relatively dormant in the blood and generally more resistant to the effects of conventional antimalarials, yet they are entirely responsible for human to mosquito transmission (Sinden, 2017).

Although huge gains have been made in reducing malaria burden since the turn of the millennium, the current control measures are threatened by the emergence and spread of parasite resistance to artemisinin-based combination therapies, along with mosquito resistance to insecticides (World Health Organization, 2022). To combat rising parasite resistance, new antimalarial drugs with alternative modes of action are critically needed (Wells et al., 2015). Transmission of the parasite from humans to mosquitoes is one of the major bottlenecks in the parasite life cycle (Graumans et al., 2020) and transmission-blocking interventions (Blagborough et al., 2013) are emerging as a key target for future antimalarial drug development (Yahiya et al., 2019; Birkholtz et al., 2022). A prime candidate for targeting the process of transmission is the dormant, circulating gametocyte that is responsible for establishing infection in the mosquito after an infected bloodmeal. Drugs that either kill or sterilise gametocytes in the human host or prevent gamete fertilisation in the mosquito gut post feed can block transmission (Birkholtz et al., 2016). Currently, however, the only approved antimalarials with defined transmission-blocking activity are 8-aminoquinolines, tafenoquine and primaquine, both being impeded by their clinical safety (Watson et al., 2021; Ashley et al., 2014). New drugs that target the transmission process could, therefore, have substantial impact in malaria control.

Gametocytes mature over five distinct morphological stages in the human host, sequestering in the bone marrow and spleen until they reach maturity (Ngotho et al., 2019). Mature stage V gametocytes are able to re-enter the host circulation, transmitting to *Anopheles* mosquitoes during a bloodmeal with subsequent gametogenesis (gamete formation) occurring in the mosquito midgut. During gametogenesis, male gametocytes transform into eight haploid microgametes (microgametogenesis), whereas female gametocytes form a single haploid macrogamete (macrogametogenesis) (Sinden et al., 1978; Sinden, 1982). Microgametogenesis is a notably complex and rapid process, involving simultaneous egress from host erythrocytes, three rounds of DNA replication alternating with endomitotic division, and the eventual formation of axonemes, which emerge as haploid microgametes during exflagellation (Sinden et al., 1978; Sinden, 1982; Yahiya et al., 2022). Gamete fusion and fertilisation give rise to diploid zygotes that, following mosquito midgut colonisation, eventually yield haploid motile sporozoites responsible for the next transmission cycle to humans (Prudêncio et al., 2006).

Efforts to identify novel drugs targeting processes from symptomatic to transmissible stages have been significantly bolstered by advances in high-throughput phenotypic screening of compound libraries (Yahiya et al., 2019). However, although phenotypic screens supersede target-based drug discovery in their ability to rapidly identify candidate drugs, the lack of knowledge on their target or mode of action can result in a lengthy process of development. Elucidation of the cellular target(s) and mode of action of a hit compound prior to clinical testing is therefore essential to improve the progress of any drug through development (Hughes et al., 2011). A recent exemplary high-throughput screen involved testing of a large diverse chemical library [the Global Health Chemical Diversity Library (GHCDL)] against both transmissible and asexual *Plasmodium* stages. The GHCDL screen identified hits with diverse activity profiles across the parasite life cycle, including hits with multistage, transmission-specific, sex-specific and stage-specific activity (Delves et al., 2018). Within the library, a novel class of compounds having an *N-*[(4-hydroxychroman-4-yl)methyl]-sulphonamide (*N-*4HCS) scaffold was found to potently inhibit the formation of *Plasmodium* male gametes from mature stage V male gametocytes. The most potent hit from the class, DDD01035881, has since been extensively studied by structure-activity relationship analyses, yielding hits with half maximal inhibitory (IC_50_) concentrations in the nanomolar range (Rueda-Zubiaurre et al., 2019). Importantly, the viability of HepG2 mammalian cells was found to be minimally impacted by treatment with DDD01035881 and its derivatives, suggesting that they have low human toxicity (Rueda-Zubiaurre et al., 2019).

To advance the *N-*4HCS chemotype for future antimalarial development, here, we sought to identify the mode of action and cellular target of DDD01035881. Using photoaffinity labelling (PAL), label-free cellular thermal shift assay (CETSA) and cellular analysis of treated parasites, we present evidence that the *Plasmodium falciparum* 16 kDa parasitophorous vacuole membrane (PVM) protein Pfs16 is a potential target of DDD01035881. Given the power of transmission-blocking therapeutics and drive for the discovery of further, novel combination therapies, these data suggest that the *N-*4HCS class of compounds might serve as an excellent foundation for future antimalarial treatment or preventative regimens and identify Pfs16 as a target for further exploration.

## RESULTS

**Fig. 1.**
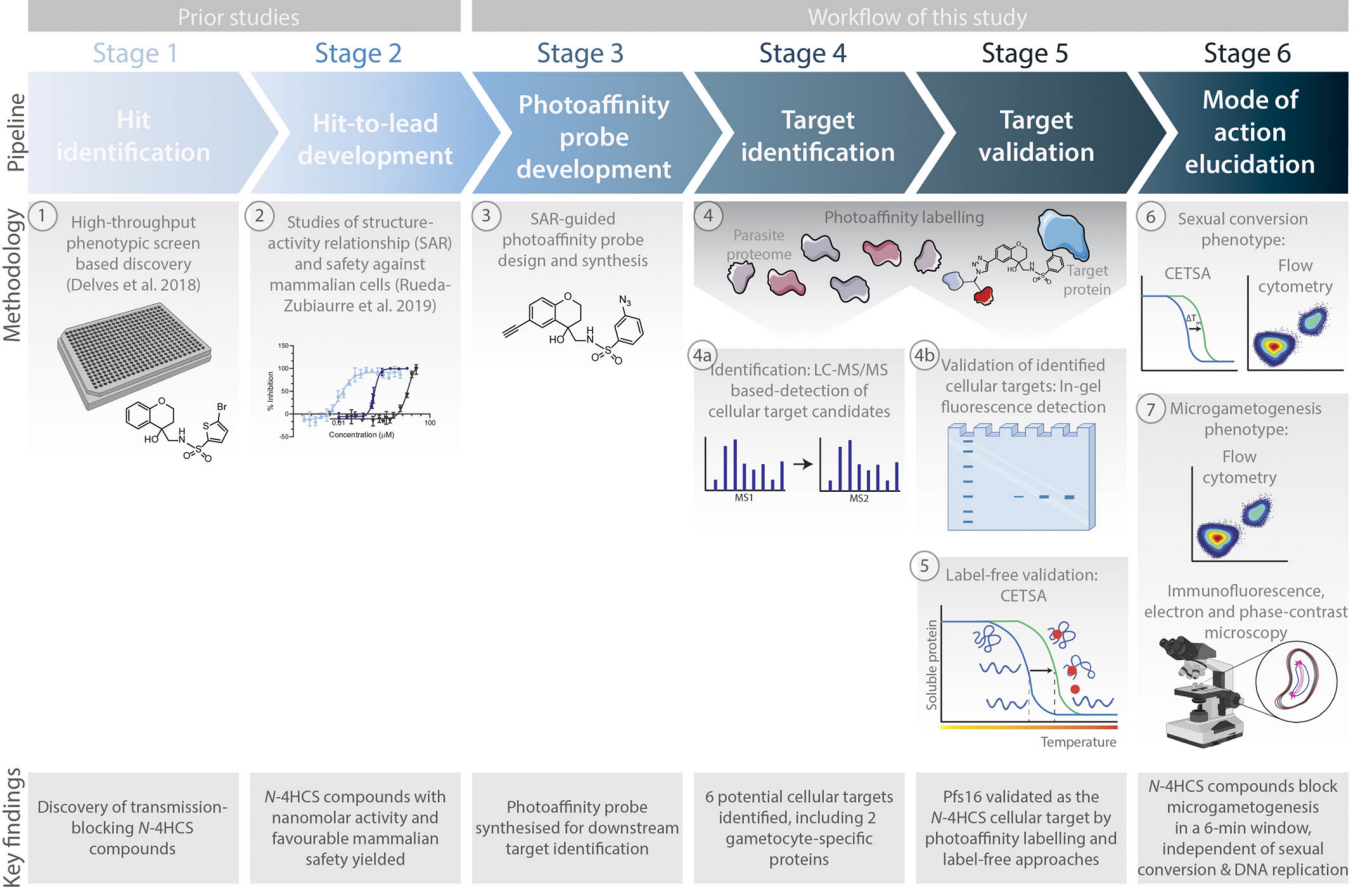
**Discovery and developmental workflow of *N-*4HCS compounds.** (1) Following initial discovery of the *N*-4HCS transmission-blocking compounds in a phenotypic screen of the Global Health Chemical Diversity library (Delves et al., 2018), a traditional drug discovery and developmental workflow was followed. (2) Hits were developed in a structure-activity relationship (SAR) study (Rueda-Zubiaurre et al., 2019) and (3) SAR knowledge guided the subsequent development of a photoaffinity labelling (PAL)-compatible probe. (4) PAL experiments were used to (4a) identify and (4b) validate cellular target candidates, using LC-MS/MS and gel-based detection methods, respectively. (5) The label-free cellular thermal shift assay (CETSA) validated PAL findings. (6) The mode of action of the *N*-4HCS compound was then elucidated by determining the gametocytogenesis and sexual conversion phenotypes by CETSA and flow cytometry. (7) The precise phenotypic impact of the compounds during microgametogenesis was determined by flow cytometry and detailed immunofluorescence, electron and phase-contrast microscopy. Elements of the figure were created with BioRender.com and adapted from Servier Medical Art (smart.servier.com; licensed under a Creative Commons Attribution 3.0 Unported License).

*N-*4HCS compounds were recently identified in a high-throughput screen for transmission-blocking antimalarials (Delves et al., 2018), with subsequent development to improve their activity by medicinal chemistry (Rueda-Zubiaurre et al., 2019). Given the potency of *N-*4HCS compounds in inhibiting *P. falciparum* microgametogenesis (Delves et al., 2018), we sought to identify their potential cellular target(s) and phenotypic effects using a range of techniques as outlined in Fig. 1.

### Synthesis of an *N-*4HCS photoaffinity probe for downstream target identification

To enable identification of cellular target(s) via PAL, we first derivatised DDD01035881 and the related compound DDD01028076, *N-*4HCS compounds discovered in the initial screen (Delves et al., 2018), to incorporate both a photoactivatable group and clickable alkyne moiety. An alkyne handle and aryl azide moiety were separately incorporated onto the *N-*4HCS scaffold to yield photoaffinity probe 2. Parent molecule 1 was synthesised to resemble the structure of probe 2 but lacking the photoactivatable and clickable moieties. Parent molecule 1 was thus able to serve as an active competitor of probe 2 by mimicking its biological activity. Critically, parent molecule 1 and probe 2 retained micromolar to nanomolar IC_50_ values in the *in vitro* male gamete formation assay (Table 1).

**Table 1.**
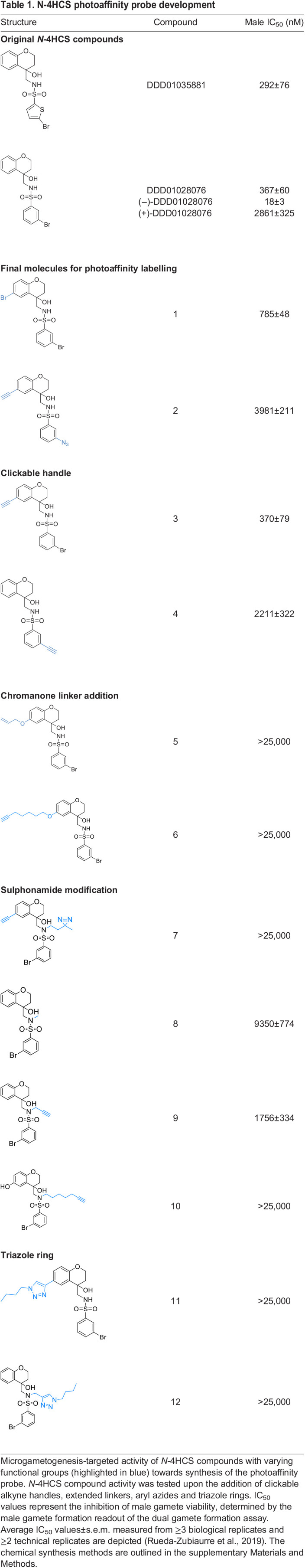
N-4HCS photoaffinity probe development

Prior to yielding final molecules 1 and 2 for PAL, we first synthesised and tested a range of *N-*4HCS structures, guided by knowledge from our previous structure-activity relationship study (Rueda-Zubiaurre et al., 2019). Firstly, *N*-4HCS activity was tested following the addition of a clickable alkyne handle, a functional group required for ligation to a fluorescent reporter. As demonstrated by the loss of a micromolar IC_50_ value of compound 4 but retention of sub-micromolar activity of compound 3, alkyne addition was favoured at the C-6 of the chromanone over the aromatic ring (Table 1). As a fully functional photoaffinity probe required both a photoactivatable moiety and alkyne handle, and having found the *N-*4HCS chromanone to be amenable to structural change, we next incorporated longer linkers to this group which might favour target binding. Extended linkers were, however, detrimental to activity (compounds 5 and 6; Table 1) and suggested that the *N-*4HCS scaffold was unable to tolerate larger groups.

As an alternate strategy, we incorporated the photoactivatable diazirine functional group onto the *N-*4HCS sulphonamide and alkyne handle onto the chromanone group, knowing that the latter modification was tolerated. These combined modifications were, however, poorly tolerated as evident by lack of activity of compound 7 (Table 1). To test whether activity was lost due to sulphonamide modification, we further modified this group, yielding compounds 8, 9 and 10. Although compounds 8 and 10 demonstrated a clear loss in activity, compound 9 retained micromolar activity, suggesting that alkyne addition at this region was feasible, whereas diazirine addition was not tolerated (Table 1).

Given our goal to perform target enrichment, two approaches were possible: (1) photoaffinity probes photo-crosslinked to cellular target(s) prior to their ligation to a reporter, for downstream enrichment with beads or (2) probe attachment to beads ahead of lysate addition, to capture protein target(s). As the latter approach relied on the retention of probe activity upon the clicking to beads, we tested whether compounds 3 and 9 remained active following click reactions that resulted in triazole ring formation. Click reactions with both 3 and 9, yielding compounds 11 and 12, respectively, hampered activity (Table 1). Thus, we focussed our efforts on pursuing the capture of cellular targets via photo-crosslinking prior to reporter ligation. Probe 2, which incorporated both an alkyne handle and aryl azide onto the *N-*4HCS scaffold, was subsequently synthesised and found to retain activity. Probe 2 was photo-crosslinked to live gametocytes *in vitro* prior to downstream target enrichment, using parent molecule 1 as an active competitor.

Preliminary testing of probe 2 was performed to test cross-linking and ligation to the azido-tetramethylrhodamine (TAMRA)/biotin capture reagent (AzTB) (Wright et al., 2015). Cell lysates derived from activated mature gametocytes were irradiated in the presence of increasing concentrations of probe 2 or DMSO to test the concentration-dependent photo-crosslinking of proteins. Lysates containing photo-crosslinked proteins were then ligated to the AzTB capture reagent via copper-catalysed azide-alkyne cycloaddition (CuAAC) prior to streptavidin enrichment. In the presence of probe 2, the enrichment was found to be AzTB dependent as measured by in-gel fluorescence (IGF), confirming successful photo-crosslinking and CuAAC (Fig. S1A).

### Gametocyte-specific *N-*4HCS target identification by chemical proteomics

**Fig. 2.**
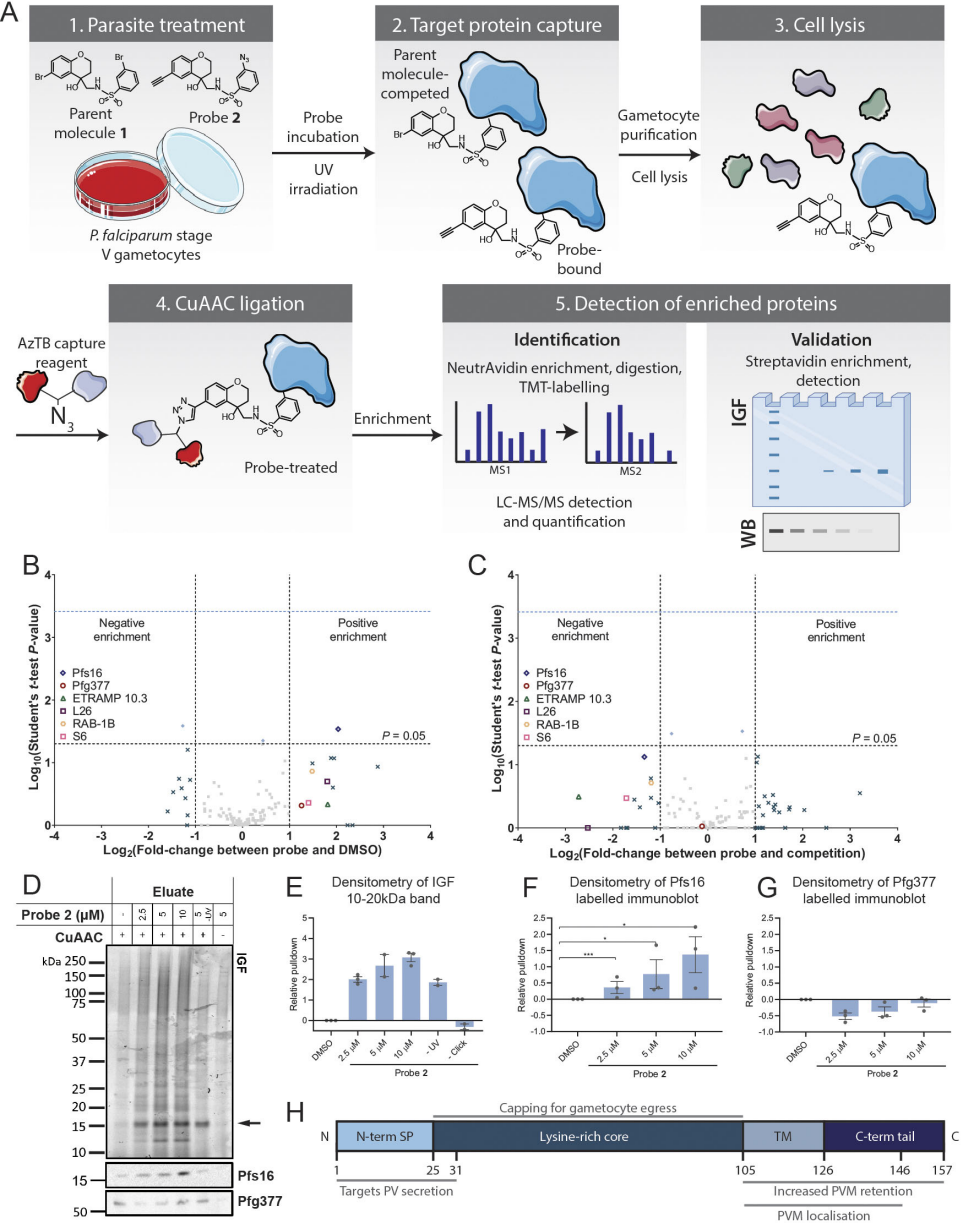
**Identification and validation of Pfs16 as a specific interaction partner with *N-*4HCS compounds.** (A) Photoaffinity-labelling workflow for *N-*4HCS target identification and validation. (1) Live stage V *P. falciparum* NF54 strain gametocytes were treated with DMSO, probe 2 or a combination of probe 2 and parent molecule 1, prior to UV irradiation to (2) capture protein targets. (3) Parasite lysates were obtained and (4) ligated to AzTB capture reagent by copper-catalysed azide-alkyne cycloaddition (CuAAC) to permit downstream target enrichment. (5) Cellular target(s) were identified by TMT labelling and nLC-MS/MS detection or validated by in-gel fluorescence (IGF) and western blotting (WB). Elements of the figure were created with BioRender.com and adapted from Servier Medical Art (smart.servier.com; licensed under a Creative Commons Attribution 3.0 Unported License). (B,C) *P. falciparum* proteome-wide target identification results. Plots depict log_10_-transformed unpaired two-sample two-tailed Student's *t*-test *P*-values against log_2_-transformed fold change in peptide-hit enrichment between (B) probe 2- and DMSO-treated samples and (C) probe 2-treated and competition (co-treatment of probe 2 and parent molecule 1) samples. Fold change was calculated from the average enrichment of three biological replicates per condition. The *P*-value of 0.05 is marked by the horizontal grey dashed line. Dark blue crosses represent proteins with a log-transformed difference in enrichment either above 1 or below −1, for positive and negatively enriched proteins, respectively. Enriched proteins with *P*<0.05 are depicted as light blue diamonds. Grey squares represent the proteins not fitting the enrichment and *P*-value thresholds. The enriched hits Pfs16 (blue cross), Pfg377 (red circle), ETRAMP 10.3 (green triangle), 60S ribosomal protein L26 (L26, purple square), Rab1B (pale orange hexagon) and 40S ribosomal protein S6 (S6, pink square) are labelled. The hits enriched in both conditions are listed in Table 2. (D,E) Validation of Pfs16 and Pfg377 binding by live probe 2 treatment, AzTB-ligation and streptavidin-biotin affinity enrichment. IGF (D) was used to identify streptavidin-enriched proteins and the corresponding immunoblots validated the specificity of pulldowns to Pfs16 and Pfg377. IGF revealed an abundantly TAMRA-labelled protein between 15 kDa and 20 kDa (black arrow), likely corresponding to Pfs16. Densitometry analysis (E) of the 16 kDa protein band with increasing probe 2 concentration, depicted relative to DMSO controls. Values depict mean±s.e.m. of three biological replicates. (F,G) Densitometry analysis of Pfs16 (F) and Pfg377 (G) band intensities from the corresponding immunoblots, relative to DMSO controls and depicted as the mean±s.e.m. of three biological replicates (see Fig. S2 for immunoblots and gels). The significance was determined with unpaired two-tailed *t*-tests. *P<0.05; ***P<0.001. (H) Predicted Pfs16 domains, consisting of an N-terminal signal peptide (SP), a lysine-rich core, a transmembrane (TM) domain and a charged residue-enriched C-terminal tail. The associated functions were described by Eksi and Williamson (2011). Parasitophorous vacuole, PV; parasitophorous vacuole membrane, PVM.

Target identification was performed using the *N-*4HCS photoaffinity probe 2 and a 9plex tandem mass tag (TMT) methodology (Fig. 2A). Live *P. falciparum* stage V gametocytes were treated with either DMSO, probe 2 (10 µM) or a combination of probe 2 and parent molecule 1 to act as a competitor (10 µM each), acquiring triplicate samples of each condition. Live treated gametocytes were irradiated at 254 nm to photo-crosslink the probe to protein targets, then purified and lysed before ligation with AzTB in a CuAAC reaction. AzTB-ligated proteins were subsequently enriched with NeutrAvidin agarose beads before preparing peptides for TMT labelling and quantification. Peptide samples were then prepared for analysis by nanoscale liquid chromatography-tandem mass spectrometry (nLC-MS/MS) on a Q Exactive orbitrap mass spectrometer.

**Table 2.**
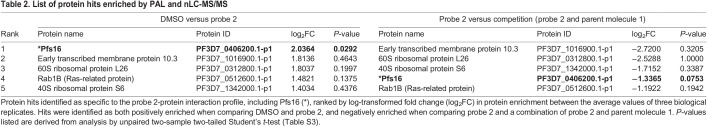
List of protein hits enriched by PAL and nLC-MS/MS

For the 125 protein hits identified (for raw data, see Table S3), the specific probe-protein interaction profile was determined by excluding any hits identified in DMSO-treated samples as non-specific-binding interactions. Comparing DMSO- and probe 2-treated fractions, probe-specific interactions revealed 13 protein hits positively enriched specifically as a result of probe 2 labelling (Fig. 2B). The specificity of probe-protein interactions is often confirmed with a competition control, in which samples are co-treated with both the probe and a parent molecule competitor, such that a more potent parent molecule would theoretically outcompete probe-target interactions. This approach was taken here by treating samples with probe 2 in the presence of parent molecule 1 and comparing protein enrichment to that of samples treated with probe 2 alone. It should be noted, however, that, given the reversible nature of *N-*4HCS compounds (highlighted in Fig. 5B), the value of the control might be questionable as photo-crosslinking might favour interactions with probe 2 (competent for cross-linking) over parent molecule 1 in competition samples (discussed in this section below). Noting this caveat, when compared in this way, outcompeted probe-specific interactions revealed 14 protein hits, depicted as negatively enriched proteins in Fig. 2C. Of these 14 hits, five were also positively enriched in the initial comparison of DMSO- and probe 2-treated fractions (Fig. 2B,C; Table 2).

Among the five protein hits specific to probe 2 (Table 2), the protein most positively enriched when comparing DMSO-treated and probe 2-treated samples was the gametocyte-specific 16 kDa *P. falciparum* PVM protein Pfs16 (PlasmoDB ID: PF3D7_0406200) (Bruce et al., 1994). Crucially, Pfs16 represented the only hit that was significantly enriched when comparing DMSO- and probe 2-treated samples (Table 2). As the *N-*4HCS compounds have been shown to solely inhibit male gametocyte viability but to exert no effect on other *Plasmodium* life cycle stages (Delves et al., 2018), Pfs16 represented the most promising *N-*4HCS-specific target candidate. Four other protein hits were identified, although their enrichment did not reach significance (unpaired two-sample two-tailed Student's *t*-test). In addition, these proteins were not specific to the gametocyte stages. We therefore deemed these four hits as the likely result of either non-specific-binding interactions or inhibition-independent binding. For example, the parasitophorous vacuole protein early transcribed membrane protein 10.3 (ETRAMP 10.3) is expressed during the asexual and liver stages (MacKellar et al., 2010), and there is evidence for transcriptional upregulation of the *etramp* 10.3 gene in gametocytes (López-Barragán et al., 2011). Similarly, the ribosomal targeted proteins L26 and S6, which were identified as hits, are unlikely targets of transmission-specific effects, given their critical role in asexual stages, stages that are not affected by *N-*4HCS chemotypes (Delves et al., 2018). Rab1B is believed to have a potential role in endoplasmic reticulum to Golgi transport (Quevillon et al., 2003) and has been shown to lie adjacent to the endoplasmic reticulum in early asexual blood stages (Taku et al., 2020). As the *rab1b* gene is predicted to be essential for asexual-blood-stage parasite growth, this would again reduce the likelihood of Rab1B being a specific target (Zhang et al., 2018). We cannot rule out poly-pharmacological activity, but, for the purpose of this study, we focused our attention on the sexual-stage specificity of *N-*4HCS compounds, prioritising the significant hit Pfs16 for further validation.

It is worth highlighting that although Pfs16 was significantly enriched upon comparison of DMSO- and probe 2-treated samples, negative enrichment of Pfs16 in probe 2-treated compared to competed samples, i.e. co-treatment of parent molecule 1 and probe 2, did not reach the assigned significance threshold. Furthermore, no proteins enriched under both conditions demonstrated significance within this comparison. Given that *N*-4HCS activity was reversed upon washing of treated parasites (see ‘The activity window of DDD01035881 coincides with that of Pfs16 during microgametogenesis’ in Results below; highlighted in Fig. 5B), it is highly plausible that irradiation of gametocytes in the presence of both probe 2 and parent molecule 1 favoured photo-crosslinking of probe 2 to the protein target. Although competition control samples often provide valuable insights into the specificity of compound binding, this was complicated by the reversibility of *N-*4HCS compound-binding and it was unsurprising that competition was not significant. Fundamentally, the significant enrichment of Pfs16 in probe 2-treated samples relative to DMSO-treated samples provided a clear indication that *N-*4HCS compounds bound and inhibited this protein.

Pfs16 is a 157 amino acid protein with a transmembrane domain (Fig. 2H) and is known to be an early marker of sexual conversion in *Plasmodium* (Lanfrancotti et al., 2007). Importantly, with respect to validating the phenotype described for *N-*4HCS compounds (Delves et al., 2018), targeted gene disruption of *Pfs16* is also known to block microgametogenesis (Kongkasuriyachai et al., 2004). Of note, the *P. falciparum* female gametocyte-specific protein Pfg377 (PlasmoDB ID: PF3D7_1250100) (de Koning-Ward et al., 2008) was also positively enriched in the probe 2-treated samples compared to the DMSO-treated samples, although this was not significant. Unlike Pfs16, Pfg377 was not negatively enriched when comparing samples treated only with probe 2 and competition samples, in which parasites were co-treated with probe 2 and parent molecule 1 (Fig. 2B). In contrast to Pfs16, Pfg377 is only associated with female gametocytes and would therefore be a less likely target of *N-*4HCS compounds, given their specific inhibition of male gamete formation. Nonetheless, as the only other gametocyte-specific candidate target, we investigated Pfg377 and Pfs16 in parallel.

### In-gel PAL validation of Pfs16 as a gametocyte-specific target of the *N-*4HCS scaffold

To validate TMT-dependent identification of Pfs16 as a target of *N-*4HCS compounds and Pfg377 as a potential non-specific binder, PAL was repeated and analysed by IGF and immunoblotting (Fig. 2A). As with the TMT identification, live mature gametocytes were treated with increasing concentrations of probe 2 and irradiated with ultraviolet (UV) light to enable bioconjugation to cellular target(s). The probe-tagged proteins present in the resulting cell lysates were biotinylated with TAMRA-containing AzTB capture reagent (Broncel et al., 2015) and pulled down with streptavidin-coated magnetic beads. Enriched proteins ligated to AzTB (and thus TAMRA labelled) were analysed by IGF, and the experiment was performed in triplicate. A notable protein band could be seen between 15 and 20 kDa, consistent with the 16 kDa protein Pfs16, demonstrating a probe 2 concentration-dependent pulldown in each replicate, which was not seen in DMSO control samples (Fig. 2D). Densitometry analysis revealed that the band intensities of the enriched protein for probe 2-treated samples, relative to bands corresponding to DMSO controls, increased with higher concentrations of probe 2 over each replicate (Fig. 2E). Corresponding immunoblotting and densitometry analyses were then used to analyse the specificity of the pulldown to Pfs16 and Pfg377 using increasing concentrations of probe 2 and antibodies specific to each of the proteins (see Fig. S2 for the full triplicate IGF data of protein pulldowns and the corresponding immunoblots). Although a dose-dependent pulldown of Pfs16 was clearly observed (Fig. 2F), there was no correlation between probe 2 concentration and the amount of Pfg377 pulled down by the probe (Fig. 2G), supporting our hypothesis of the latter being a non-specific interaction. Dose-dependent pulldown of Pfs16 further supports its being the likely target of *N-*4HCS compounds.

Pulldowns were additionally performed on parasites treated with a combination of probe 2 and the parent compound DDD01035881 acting as a competitor. In the presence of the more potent parent compound, probe 2 would theoretically have been outcompeted and would subsequently reduce the pulldown of the protein target. However, given the reversibility of the *N-*4HCS compounds, the irreversible nature of photo-crosslinking could still favour probe 2 binding. Although enrichment of a 15-20 kDa band by IGF was significantly reduced in the DMSO-treated samples relative to probe 2-treated samples, 2.5 and 10 µM DDD01035881 did not significantly reduce the extent of pulldown (Fig. S3). This served as additional evidence that photo-crosslinking of probe 2 surpassed the increased potency of DDD01035881. It should be noted that 0.1 µM DDD01035881 did appear to increase the extent of pulldown of the 15-20 kDa protein, highlighting variability in the experiment (Fig. S3). Fundamentally, probe 2 consistently increased pulldown of a 15-20 kDa protein, likely representing Pfs16.

### Label-free validation of Pfs16 as a potential target of DDD01035881

**Fig. 3.**
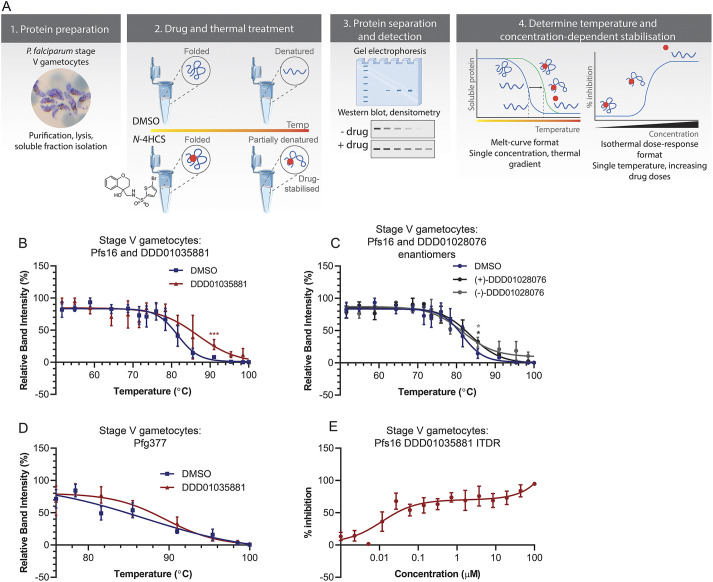
**Label-free validation of Pfs16 interaction with DDD01035881 in mature gametocytes.** (A) Workflow and premise of CETSA. (1) Stage V gametocytes were purified and lysed, and the soluble protein fraction was isolated. (2) The soluble proteins were then treated with an *N-*4HCS compound or DMSO prior to thermal challenge. In the presence of the drug, protein targets were stabilised such that the level of temperature-induced denaturation was reduced compared to a vehicle control. (3) Proteins not stabilised by drug treatment were separated by gel electrophoresis and detected by immunoblotting. (4) Densitometric analysis of immunoblots were used to obtain melting curves and isothermal dose response curves. Elements of the figure were created with BioRender.com and adapted from Servier Medical Art (smart.servier.com; licensed under a Creative Commons Attribution 3.0 Unported License). (B-E) Target validation by CETSA. (B) Pfs16 and (D) Pfg377 melt curves measured with activated stage V gametocyte lysates, treated with DMSO or DDD01035881. The mean±s.e.m. of two to three biological replicates are shown. (C) Pfs16 melting curves in the presence of DMSO, (−)-DDD01028076 or (+)-DDD01028076 using stage V gametocyte lysates. The significance was measured using unpaired two-tailed *t*-tests and. *P<0.05; ***P<0.001. (E) The corresponding isothermal dose response (ITDR) curve of B depicting the concentration-dependent stabilisation of Pfs16 by DDD01035881 in activated stage V gametocyte lysates, relative to the highest and lowest band intensities. Average values±s.e.m. of four biological replicates are shown (see Fig. S4 for full immunoblots).

PAL is known to be sensitive to false-positive results, with clickable derivatives potentially binding non-specifically to proteins other than the target of interest (Smith and Collins, 2015). To circumvent this issue and validate the specificity of DDD01035881 engagement with Pfs16 and Pfg377, label-free CETSA was used with lysates from mature gametocyte cultures and the original DDD01035881 compound (Fig. 3A). CETSA is based on the premise that proteins irreversibly aggregate when thermally challenged and the modulation of a given protein when bound to a ligand can alter this process, resulting in increased thermal stability and melting temperature (T_m_) of the protein (Martinez Molina et al., 2013). CETSA was performed on parasite lysates, applying the lysis conditions used in the PAL target identification study. Parasites were lysed with a 1% Triton X-100-containing lysis buffer, which was expected to solubilise a PVM protein target such as Pfs16, prior to compound treatment and thermal challenge.

Mature gametocyte cell lysates from activated stage V gametocytes were incubated with either DDD01035881 or DMSO as a control, thermally challenged and probed by immunoblotting using a Pfs16-specific antibody (Bruce et al., 1994) to explore the engagement of DDD01035881 and Pfs16. Pfs16 melt curves were obtained to compare the T_m_ between DMSO-treated and DDD01035881-treated soluble gametocyte protein fractions (Fig. 3). DDD01035881 treatment clearly resulted in a positive shift in the Pfs16 melting curve (Fig. 3B). When compared to DMSO-treated fractions, the relative band densities of DDD01035881 treated fractions were found to be significantly different at 91°C (unpaired two-tailed *t*-test, *P*<0.001). A shift in the Pfs16 melting curve was also evident in the presence of (−)-DDD01028076 and (+)-DDD01028076, the separated enantiomers of a potent analogue of DDD01035881 (Fig. 3C). A significant difference in the relative band intensity between the enantiomer and DMSO-treated samples was measured at 85.5°C (unpaired two-tailed *t*-test, *P*<0.05). Although the shifts in Pfs16 melting curves do not reflect the >100-fold difference in *in vitro* IC_50_ values (Table 1), this is not surprising given that CETSA was performed at 100 µM to observe target engagement. The lack of differential between the enantiomer curves is therefore a likely result of saturated target engagement. Critically, using the same approach, DDD01035881 did not show significant stabilisation of Pfg377 at any temperature investigated (Fig. 3D). This aligns with the published female-specific role of Pfg377 in macrogametogenesis (de Koning-Ward et al., 2008) and lack of activity of DDD01035881 against female gametocytes. These results strongly support a specific interaction between DDD01035881 and Pfs16 in mature activated male gametocytes, corroborating the PAL results. The lack of interaction with the female-specific Pfg377 suggests its detection by PAL was a false positive, consistent with our understanding that DDD01035881 specifically targets males.

To further validate the positive shift in the T_m_ of Pfs16 following DDD01035881 treatment of activated gametocyte lysates, an isothermal dose response (ITDR) format of CETSA was applied. ITDR-CETSA utilises the same premise as the melt curve format, except that the proteins are thermally challenged with a single temperature and treated with varying compound concentrations (Black et al., 2010). The single temperature applied in ITDR-CETSA is that at which Pfs16 is mostly aggregated in the DMSO-treated fraction but is largely stabilised in the DDD01035881 fraction (derived from the melt curve, Fig. 3B). ITDR-CETSA with DDD01035881 was performed at 78.4°C using concentrations between 1 nM and 100 µM with the results analysed by immunoblotting. A clear concentration-dependent stabilisation of Pfs16 was seen (Fig. 3E), adding substantial support to Pfs16 being a specific interactor of the label-free DDD01035881.

### DDD01035881 specifically inhibits microgametogenesis without impacting gametocytogenesis

**Fig. 4.**
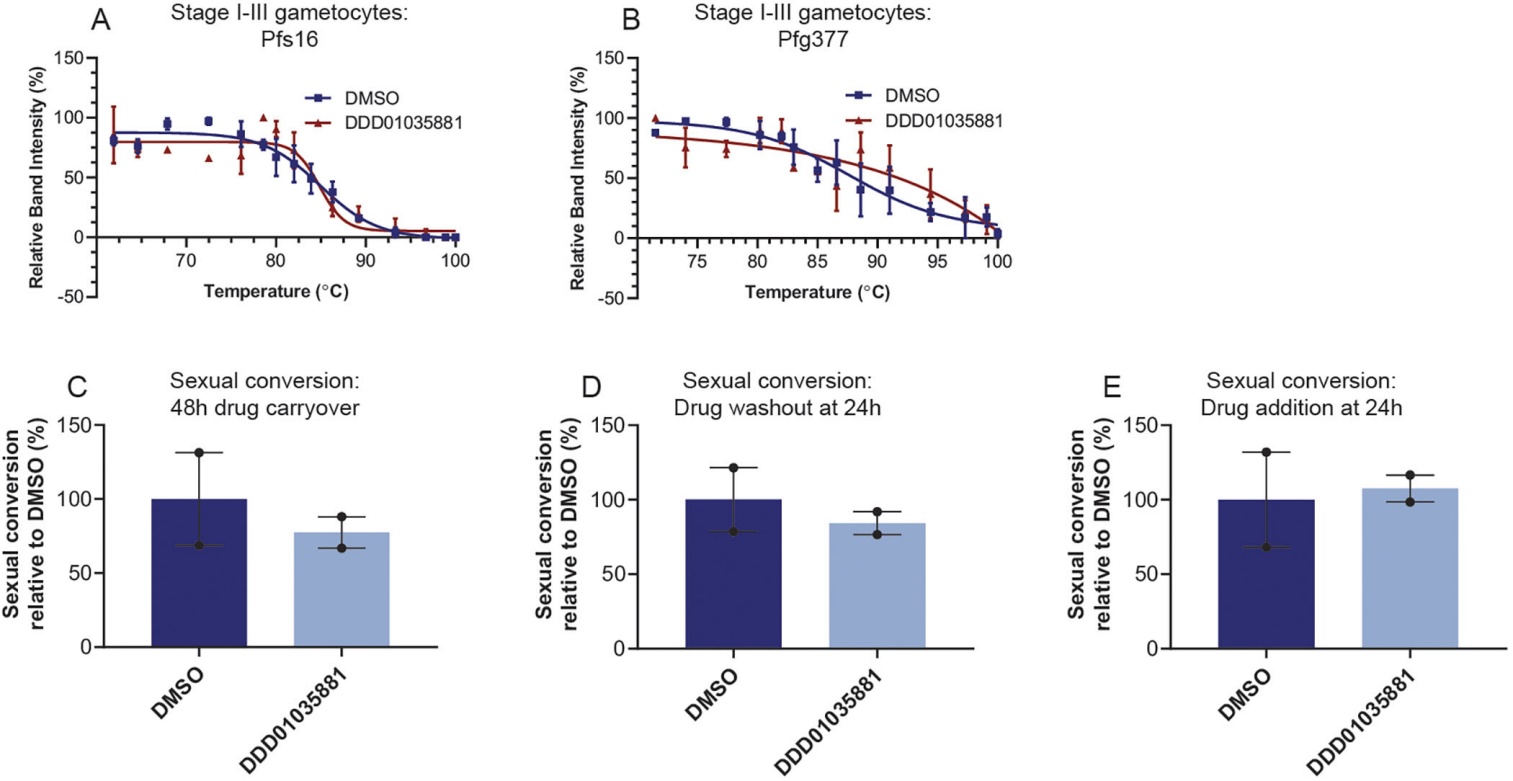
**Effect of *N-*4HCS compounds on gametocytogenesis.** (A,B) DDD01035881 engagement with (A) Pfs16 and (B) Pfg377 in stage I-III gametocyte lysates as determined by CETSA, depicted as the mean±s.e.m. of two to three biological replicates (full immunoblots in Fig. S5). No significant DDD01035881-induced stabilisation of Pfs16 or Pfg377 was measured relative to DMSO. (C-E) Sexual conversion rate of *Pf*2004/164-tdTomato parasites determined by tdTomato expression following gametocytogenesis induction. The conversion rates of DDD01035881-treated parasites are expressed as rates relative to DMSO-treated parasite conversion rates. Perturbations to sexual conversion were determined by maintaining treatment over two intraerythrocytic cycles (C) and the reversibility of any perturbations was determined by removing the compound at 24 h (D). Perturbations to early gametocyte development were probed by administration of compounds in a subsequent intraerythrocytic cycle (E). See Fig. S6A-D for the gating strategy and conversion rates of other *N-*4HCS compounds. All conversion rates were calculated using the initial parasitaemia (100,000 cells) and subsequent gametocytaemia (400,000 cells). Error bars represent the s.e.m. of two biological replicates.

Pfs16 is reported to be an early marker of sexual conversion, with gene disruption studies suggesting that it plays a crucial role in commitment to gametocytogenesis (Kongkasuriyachai et al., 2004). We sought to study the effect of DDD01035881 treatment on sexual conversion and early gametocyte development. To determine the stage specificity of Pfs16 binding, CETSA was performed on immature gametocyte cell lysates derived from stage I-III gametocyte culture. Stage I-III gametocyte lysates were treated with DDD01035881 and thermally challenged to quantify the stabilisation of Pfs16 and Pfg377. No statistically significant difference was found between the DMSO- and DDD01035881-treated fractions for either Pfs16 (Fig. 4A) or Pfg377 (Fig. 4B). These findings suggest that DDD01035881 specifically binds Pfs16 in mature gametocytes, with no binding observed for Pfs16 in immature gametocytes or Pfg377 at any stage.

The stage specificity of Pfs16 binding *in vitro* was validated by measuring the conversion rates of a transgenic *P. falciparum* line, which expresses tdTomato at the point of sexual conversion (*Pf*2004/164-tdTomato; Brancucci et al., 2015), measured by flow cytometry (Fig. S6A-D). Here, gametocytes were treated with either DDD01035881 or DMSO under multiple conditions to quantify: (1) inhibition of sexual conversion (Fig. 4C), (2) the reversibility of any inhibitory effect on conversion (Fig. 4D) and, finally, (3) effects on early gametocyte development (Fig. 4E). The respective conditions were: (1) prolonged compound exposure from the point of induction (Fig. 4C), (2) compound removal 24 h post induction (Fig. 4D) and (3) late compound treatment 24 h after induction (Fig. 4E; the gating strategy and conversion rates of additional *N-*4HCS compounds can be found in Fig. S6A). We found no significant reduction in relative conversion rates under any of the three treatment conditions, suggesting that DDD01035881 acts specifically during microgametogenesis and not during sexual conversion or early gametocyte development.

### The activity window of DDD01035881 coincides with that of Pfs16 during microgametogenesis

**Fig. 5.**
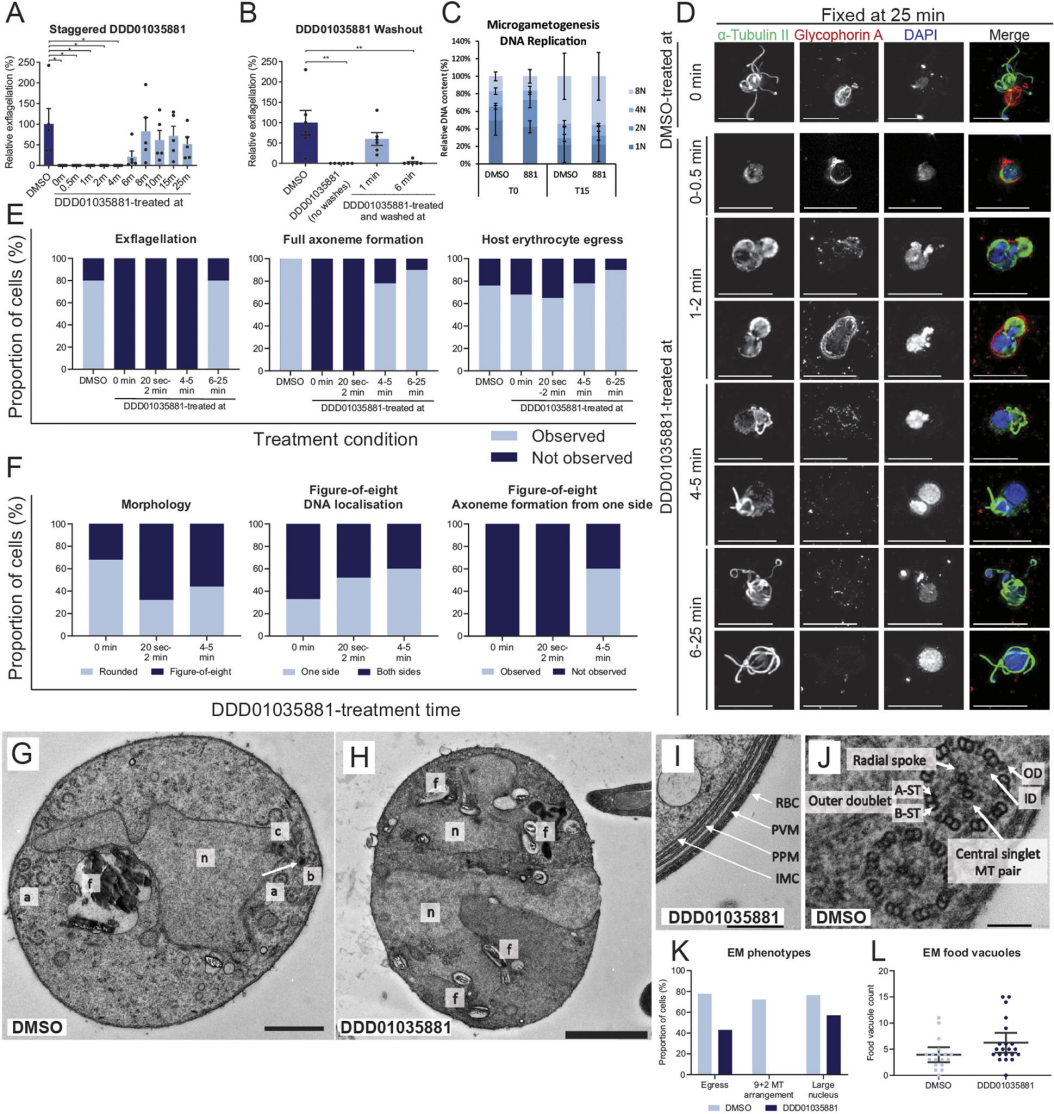
**Microgametogenesis phenotype under DDD01035881 treatment.** (A,B) Exflagellation rates at 25 min relative to DMSO controls which were either (A) activated in the absence of the drug, then treated with 5 µM DDD01035881 at the stated time points, or (B) treated with DDD01035881 or DMSO, then activated and washed at the labelled time points for compound removal. Average rates±s.e.m. of five to six biological replicates are shown. The significance was determined with unpaired two-tailed *t*-tests. *P<0.05; **P<0.01. (C) Relative DNA content of *PfDyn*GFP/*P47*mCherry male gametocytes at 0 and 15 min post activation with DMSO or DDD01035881 (881) treatment. Parasite ploidy (1N, 2N, 4N or 8N) was determined by Vybrant DyeCycle intensity by flow cytometry, depicted as averages±s.e.m. of three biological replicates, measuring 100,000 cells per sample. (D) Morphological phenotype of DDD01035881-treated male gametocyte parasites, determined by immunofluorescence microscopy, depicting the α-tubulin-labelled cytoskeleton (green), glycophorin A-labelled erythrocyte (red) and DNA (blue). Gametocytes were treated at the stated time points relative to microgametogenesis activation and fixed at 25 min post activation. Scale bars: 10 µm. (E) Quantification of the proportion of cells exhibiting exflagellation (left), axoneme formation (middle) and egress (right) phenotypes compared to DMSO controls, determined by immunofluorescence imaging. Data are representative of 142 cells in total from three biological replicates per condition. (F) Quantification of DDD01035881-specific phenotypes that were characterised based on rounded or figure-of-eight morphologies (left), subsequently measuring DNA localisation (middle) and gamete formation of parasites displaying a figure-of-eight morphology (right). Data are representative of 97 cells in total from three biological replicates per condition. (G-J) EM images of DDD01035881- or DMSO-treated gametocytes that were activated and fixed at 25 min. (G) DMSO-treated gamete preparing for emergence. The kinetosomal sphere and granule (white arrow) and kinetosomal basket (‘b’) are located at the centriolar plaque within the nucleus (‘n’) and adjacent to the food vacuole (‘f’), bearing an intranuclear spindle and chromatin (c). Several 9+2 microtubule arrangements (‘a’) can be at the cell periphery, preparing for axoneme emergence. Scale bar: 1 µm. (H) DDD01035881-treated microgametocyte with a disrupted nuclear structure (‘n’) and food vacuoles (‘f’). Scale bar: 2 µm. (I) DDD01035881-treated microgametocyte failing to egress, displaying an intact four-layer membrane. Inner membrane complex, IMC; parasite plasma membrane, PPM; parasitophorous vacuole membrane, PVM; red blood cell, RBC. Scale bar: 500 nm. (J) Characteristic 9+2 arrangement of microtubules, with the ‘A’ and ‘B’ sub-tubule (‘ST’) pairs spaced around the central singlet microtubule (MT) pair via radial spokes. Inner dynein arms, ID; outer dynein arms, OD. Scale bar: 100 nm. (K,L) EM phenotypes were quantified to measure the proportion of cells exhibiting egress, 9+2 microtubule arrangement and enlarged nuclei (K), and food vacuole number (L) in DMSO and DDD01035881-treated parasites. Data are representative of 39 cells from one biological replicate. Error bars in L show the mean±s.e.m.

Having defined Pfs16 as a likely target for the *N-*4HCS compounds, we next assessed the precise cellular phenotype of the parent molecule DDD01035881, beginning with defining its window of action during microgametogenesis. As DDD01035881 inhibits microgametogenesis without requiring prior incubation with gametocytes, we hypothesised that the compound might continue to exert inhibitory activity beyond gametocyte activation. To define an activity window for *N-*4HCS compounds, gametocytes were activated in the absence of any drug and subsequently treated with DDD01035881 in time increments up to the point of exflagellation. Exflagellation rates were counted at 25 min post activation and calculated as a percentage relative to DMSO controls. As depicted in Fig. 5A, DDD01035881 inhibited microgametogenesis up to 6 min following activation. Of note, this time window is consistent with presence of the PVM, the membrane in which Pfs16 is found, the function and breakdown of which plays a part in microgametogenesis (Andreadaki et al., 2018).

To determine whether DDD01035881 binding is reversible during the active window, gametocytes were next treated with DDD01035881 at the point of activation and washed to remove the compound, and exflagellation rates were measured. The reversibility of DDD01035881 treatment was shown to be time dependent. Removal of the compound at 1 min restored exflagellation; however, no exflagellation was seen when the compound was removed at 6 min (Fig. 5B), the point at which DDD01035881 loses activity. Combining these observations, we conclude that inhibition by DDD01035881 reversibly blocks microgametogenesis within a 6 min activity window post gametocyte activation. Beyond 6 min post activation, the Pfs16-containing PVM (Baker et al., 1994; Bruce et al., 1994; Lanfrancotti et al., 2007) is lost due to microgametocyte egress (Andreadaki et al., 2018) and, hence, loss of DDD01035881 activity beyond the 6 min window points to reversible binding to a target related to PVM function. Given the evidence for Pfs16 binding by DDD01035881, the downstream inhibition of exflagellation suggests a corresponding window of Pfs16 activity.

### DDD01035881 treatment does not impact ploidy during microgametogenesis

We next sought to decipher whether DDD01035881 treatment plays a role in DNA replication, one of the key events in microgametogenesis. The ploidy of gametocytes was determined by flow cytometry analysis of a transgenic parasite, *PfDyn*GFP/*P47*mCherry, which expresses GFP in male or mCherry in female gametocytes (Fig. S6E) (Lasonder et al., 2016). Vybrant DyeCycle Violet staining was used as a measure of male gametocyte DNA content at 0 and 15 min post activation. To measure ploidy, the GFP and Vybrant DyeCycle Violet double-positive gametocyte population was gated, from which discrete populations of 1N, 2N, 4N and 8N male gametocytes could then be measured (Fig. S6E-H).

For DMSO-treated control parasites at 0 min, gametocytes with a 1N genome were the most abundant, with a smaller proportion having 2N, 4N or 8N genomes, the latter smaller proportion likely the result of premature activation or selective gene amplification (Janse et al., 1988). Conversely, most gametocytes had an 8N genome at 15 min post activation, indicative of three successful rounds of DNA replication (Janse et al., 1988). A reduced proportion of gametocytes failed to fully replicate DNA, with 1N, 2N and 4N genomes found at different ratios. Ploidy of DDD01035881-treated and DMSO-treated gametocytes were found to be similar at both 0 and 15 min post activation, with no statistically significant differences found (Fig. 5C). Thus, treatment with DDD01035881 does not disrupt DNA replication during microgametogenesis. By extension, this suggests that Pfs16 does not function in or signal upstream of DNA replication (Fig. 5C).

### DDD01035881 treatment disrupts cytoskeletal, nuclear and food vacuole structure

Having defined that DDD01035881 activity is specific to microgametogenesis without impacting gametocytogenesis, we next sought to define the compound phenotype during microgametogenesis. Perturbances to microgametogenesis under 5 µM DDD01035881 treatment were analysed by either immunofluorescence (IF) microscopy or electron microscopy (EM) (Fig. S7). For IF analysis, gametocytes were activated in the absence of the drug and then treated with DDD01035881 at various timepoints relative to activation, before being fixed and stained for analysis. As shown in Fig. 5D, DDD01035881 treatment resulted in a time-dependent perturbance to microgametogenesis, with distinct phenotypes observed depending on the time of treatment relative to activation. DDD01035881 treatment at 0-0.5 min post activation blocked exflagellation and the cytoskeletal rearrangement of parasites, with gametocytes failing to form mitotic spindles or axonemes (Fig. 5D,E). The gametocytes succeeded in rounding up, but the egress phenotype was mixed, with some parasites failing and some succeeding to egress from the host erythrocyte (Fig. 5D,E). Following DDD01035881 treatment at 1-2 min post activation, exflagellation failed and the parasite cytoskeleton adopted a figure-of-eight morphology (Fig. 5D,F). The mixed egress phenotype was retained, with erythrocyte vesicles remaining close to the parasite as expected in microgametogenesis (Andreadaki et al., 2018); those failing to egress also demonstrated some erythrocyte vesiculation, although to a lesser extent. DNA staining suggested that replication was successful, with DNA either localising to one or both sides of the figure-of-eight structure (Fig. 5F). Similarly, DDD01035881 treatment at 4-5 min post activation resulted in a cytoskeletal figure-of-eight morphology, but egress and erythrocyte vesiculation were successful. However, a truncated flagellum formed from the larger side of the figure-of-eight structure, with tubulin staining markedly more diffuse on the opposing end of the parasite (Fig. 5D,F). Following DDD01035881 treatment at 6-25 min post activation, exflagellation appeared to match that of the DMSO control (Fig. 5D), specifically in axoneme formation, DNA replication and egress from host erythrocytes, although the onwards viability of gametes was not determined. Again, by extension, this points to a potential function of Pfs16 upstream of cytoskeletal rearrangements that underpin microgamete development.

EM was used to bring ultrastructural resolution to the DDD01035881 phenotype. Gametocytes were treated with either DDD01035881 or DMSO prior to activation and fixed at 25 min post activation. Fig. 5G shows an example of a DMSO-treated control gametocyte preparing for exflagellation, depicting the 9+2 organisation of microtubules that is characteristic of axonemes, which, upon full formation, emerge from the cell body of gametocytes (Fig. 5J). In contrast, gametocytes treated with DDD01035881 prior to activation demonstrated a disruption to the structural integrity of the nucleus and food vacuole (Fig. 5K), with multiple haemozoin-containing vesicles dispersed across the cytosol of the parasite (Fig. 5H,L). This finding was consistent with the phenotype of previously described lines with targeted disruption of the *Pfs16* gene (Kongkasuriyachai et al., 2004). The mixed egress phenotype, quantified in Fig. 5K, was visualised with gametocytes lacking (Fig. 5H) and retaining the four-layer membrane (Fig. 5I).

Of note, DDD01035881 IF phenotypes were markedly different to those of the inhibitors of calcium-dependent protein kinase 4 (CDPK4) (inhibitor 1294; Ojo et al., 2014) or cyclic-GMP dependent protein kinase (PKG) (inhibitor ML10; Baker et al., 2017), or canonical inhibitors of microtubules (colchicine) or actin microfilaments (cytochalasin B) (Fig. S8). The activity windows of 1294 and ML10 (Fig. S8C,D), corresponding to 0-20 s and 0-8 min, respectively, also differed from the 6 min DDD01035881 activity window, suggesting that *N-*4HCS compound activity has a discrete function to that of these known regulators of microgametogenesis.

### DDD01035881-treated parasites demonstrate disruption of parasitophorous vacuole and Pfs16 release

**Fig. 6.**
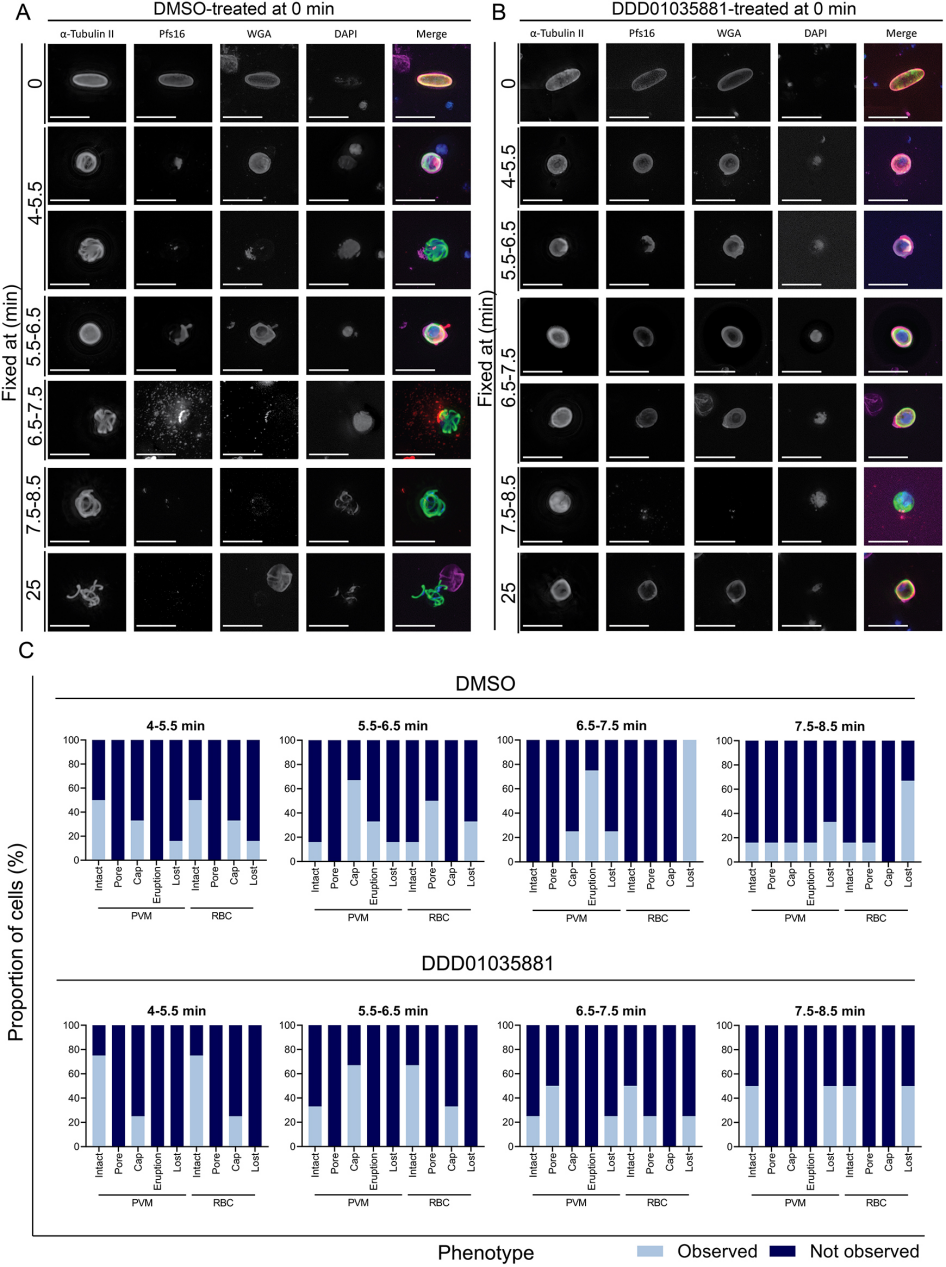
**Pfs16 localisation during microgametogenesis with and without DDD01035881 treatment.** (A,B) Representative images of microgametogenesis time-courses displaying localisation of the PVM protein Pfs16 (red) during microgametocyte egress, co-stained for α-tubulin (green), the erythrocyte membrane (WGA, magenta) (Hale et al., 2017) and DNA (blue). Scale bars: 10 µm. (A) DMSO-treated gametocytes depicting Pfs16 capping or gathering at a pore at 5.5 min post activation, as the PVM prepared to disintegrate prior to erythrocyte egress. Capped or pore-localised Pfs16 vesiculated and burst at around 6.5 min during egress, and Pfs16 was not detected following egress. (B) Mixed phenotypes of DDD01035881-treated gametocytes, either failing to egress (retained Pfs16 localisation at the PVM) or successfully egressing (Pfs16 capping but no vesiculation). (C) Quantified phenotypes from A,B depicting the proportion of cells in which the PVM and host erythrocyte (RBC) were intact, formed a pore, capped, erupted (PVM only) or were completely lost. Phenotypes were quantified based on Pfs16 labelling (PVM) or WGA staining (erythrocyte). Data are representative of 40 cells in total from three biological replicates per condition.

Finally, having defined when the compound is active, we next sought to correlate DDD01035881 action with the cellular distribution of and effects on Pfs16 directly during microgametogenesis (Kongkasuriyachai et al., 2004). Pfs16 is localised to the PVM (Baker et al., 1994; Bruce et al., 1994; Lanfrancotti et al., 2007), which vesiculates and disintegrates prior to host erythrocyte egress, in an inside-out mechanism of egress during microgametogenesis (Andreadaki et al., 2018). Following egress, Pfs16 has been detected in both so-called ‘Garnham’ bodies and multi-laminated whorls formed from the PVM after rupture (Baker et al., 1994). By IF labelling of Pfs16, we show that Pfs16 surrounded microgametocytes at 0 min before either capping or forming a pore at a single side of the activated gametocyte in preparation for egress at around 5.5 min (Fig. 6A). Upon host erythrocyte egress, Pfs16 was often observed to localise to vesicles that were expelled from the single pore or cap at 6.5 min. Minimal remnants of Pfs16 remained attached to the parasite from 8.5 min, with no Pfs16 detected on microgametes at 20 min (Fig. 6A).

At egress, DDD01035881 treatment was found to disrupt Pfs16 localisation with two distinct phenotypes (Fig. 6B). In the first phenotype, gametocytes failed to egress from the host erythrocyte with Pfs16 retaining localisation at the PVM that, similar to the erythrocyte, does not disintegrate or vesiculate. In the second phenotype, successful but aberrant erythrocyte egress was detected, as Pfs16 capped to a single end of the parasite but failed to vesiculate and erupt from a pore, as evident at 7.5-8.5 min (Fig. 6B,C). With the latter phenotype, minimal remnants of Pfs16 were detected at 8.5 min, with no detectable wheat germ agglutinin (WGA) staining. These results demonstrate that DDD01035881 inhibition often disrupts the expulsion of the PVM and associated Pfs16 localisation during microgametogenesis. The combined experimental data therefore not only points to Pfs16 being the target of the *N-*4HCS scaffold, but also corroborates the key role of Pfs16 in PVM degradation as a critical step in microgametogenesis.

## DISCUSSION

As resistance inevitably threatens the long-term success of artemisinin and its derivatives in treating malaria, there is an urgent need for new antimalarials with novel chemotypes and modes of action (Burrows et al., 2017). Here, we have validated the cellular effects of a potent class of transmission-blocking compounds based on an *N-*4HCS scaffold and identified the *P. falciparum* 16 kDa protein Pfs16 as a likely cellular target of the compounds. The hit compound series was first identified in a phenotypic high-throughput screen (Delves et al., 2018), with hits further optimised by medicinal chemistry (Rueda-Zubiaurre et al., 2019). With identification of a potential target and its low cytotoxicity, the *N-*4HCS scaffold is clearly poised for development as a combination, transmission-blocking therapeutic.

Based on detailed phenotypic analysis, *N-*4HCS compounds specifically result in the potent inhibition of microgametogenesis [fitting with the Medicines for Malaria Venture target candidate profile 5 (TCP5); Burrows et al., 2017], although without impacting sexual conversion or early gametocyte development. The phenotypic effect of the compounds is clear and rapid, traversing the erythrocyte membrane and acting within the first 0-6 min post activation of gametocytes. The activity window supports evidence that the target is vital to microgametogenesis in its earliest stages, during which the PVM remains associated with the parasite. Beyond PVM expulsion from 6 min, the effect of the compound stops, suggesting that the target protein or proteins are no longer essential beyond this point. Evidence suggesting that Pfs16 is a potential cellular target of the *N*-4HCS compounds fits with studies highlighting an impact to microgametogenesis upon the targeted gene disruption of *Pfs16* (Kongkasuriyachai et al., 2004).

An inconsistency that was noted is that DDD01035881-treated parasites did not show any defect in gametocyte commitment, which might otherwise be expected for a compound targeting Pfs16, an early marker of sexual conversion. An explanation for this might relate to the *N*-4HCS binding site in Pfs16. A previous study on protein trafficking across the parasitophorous vacuole to the PVM in *P. falciparum* used parasites transformed with different Pfs16-GFP constructs to determine the amino acid sequences required for PVM targeting and retention (Eksi and Williamson, 2011). Interestingly, the study suggested that the region for PVM targeting and retention differs from the region required for capping during gametogenesis, supporting a dual role for the protein (Fig. 2H). PVM targeting and retention was shown to be sufficient with the inclusion of the 53 C-terminal amino acids of Pfs16, containing a motif conserved in other known PVM proteins, and, hence, an unlikely target of a microgamete-specific compound. The specific signal for PVM targeting was localised to the 42 amino acids constituting the transmembrane domain (22 amino acids) and part of the C-terminal tail (20 of the 31 amino acids). The protein membrane interaction was found to be stabilised with 11 C-terminal amino acids, which, when removed, reduced the level of retention but did not affect PVM targeting. In contrast, amino acids between the N*-*terminal secretory signal sequence and transmembrane domain were crucial for capping involved in egress during gametogenesis, although they were not required for PVM targeting (Eksi and Williamson, 2011). This difference in function of the different regions of the Pfs16 protein might clarify why *N-*4HCS compounds specifically target gametogenesis without impacting commitment to gametocytogenesis, despite the protein being present in both stages. *N-*4HCS compounds might feasibly bind the region involved in capping, which is suggested to be located within the parasitophorous vacuole in association with the gametocyte surface. In contrast, the region involved in membrane retention is shown to be conserved in other PVM proteins and therefore unlikely to be the target. Given that the episomal expression of integral PVM proteins tagged with a fluorescent protein can result in parasitophorous vacuole localisation, the study of Eksi and Williamson (2011) and conclusions they made might need further corroboration. Crucially, the structure of Pfs16 remains elusive, along with details on any possible post-translational modifications or complex formations. Additional structural investigation of the specific binding of DDD01035881 to the Pfs16 protein would certainly validate the hypothesis of a direct interaction.

DDD01035881 has demonstrated potent inhibition of both *P. falciparum* and *Plasmodium berghei* transmission, blocking *in vivo* oocyst formation in both species (Delves et al., 2018). Previous studies have demonstrated that the compound blocked *in vitro* ookinete formation in *P. berghei* (Delves et al., 2018), in addition to the potent block in *P. falciparum* exflagellation demonstrated both here and in preceding studies (Delves et al., 2018). These findings suggest that Pfs16 is fundamental to transmission in *P. berghei*. However, knockout of the putative, although divergent, Pfs16 orthologue expressed in *P. berghei* (Pbg16; PlasmoDB ID: PBANKA_1003900) has been reported not to impair gametocyte production, exflagellation or ookinete formation (Deligianni et al., 2018). *Pbg16* knockout did, on the contrary, reduce oocyst formation, pointing to its potential importance in the transition from ookinete to oocyst (Deligianni et al., 2018). Although our understanding of *N-*4HCS compounds and their mode of action does not seem to align with Pbg16 function, these inconsistencies might be explained by several hypotheses relating to either divergence in protein function between the two species or the divergence in their sexual-stage biology. Of note, prior studies on the DDD01035881 phenotype in *P. berghei* focused only on ookinete and oocyst formation, whereas impediments to microgamete viability or exflagellation were not measured (Delves et al., 2018). Further investigation into the structural and species-specific impacts of *N-*4HCS compounds would be of value to resolve these questions.

The immediate and potent activity of DDD01035881 upon gametogenesis activation (and proven activity in a murine *in vivo* model in this context; Delves et al., 2018) gives confidence about the ability of this class of molecules to halt parasite development in the mosquito. However, for effective inhibition of gametogenesis to occur following a mosquito feed, the compound would need to exhibit protracted longevity in host circulation to be taken up by a feeding mosquito. Evidence suggests that this likely represents the key challenge for this class of molecule, as parasite transmission is blocked upon the introduction of DDD01035881 to an infectious bloodmeal 30 min prior to mosquito exposure, but efficacy is lost with treatment 24 h prior to a bloodmeal (Delves et al., 2018). It might, therefore, be desirable to maximise the longevity and bioavailability of *N*-4HCS compounds, with either further medicinal chemistry or formulation for slow release, such as via engineering nanoparticles or other substrates that facilitate slow release in the blood stream (Kamaly et al., 2016). However, the microgametogenesis-specific activity of *N*-4HCS compounds would be best capitalised by targeting treatment directly to the vector, serving essentially as antimalarials for mosquitoes. Recent work has simulated the impregnation of bed nets or attractive sugar baits with antimalarials to target transmission (Fiorenzano et al., 2017; Paton et al., 2019), demonstrating that the cytochrome b inhibitor atovaquone is efficacious in blocking *P. falciparum* transmission by tarsal exposure (via the mosquito legs) (Paton et al., 2019). Such an approach might complement the immediate microgametogenesis-targeted activity of the *N-*4HCS compounds. A recent study reported a reduction in *P. falciparum* transmission in the presence of an anti-Pfs16 polyclonal antibody (Niu et al., 2021), proving this protein to be of further interest in vaccine-mediated malaria control or antibody-based therapies.

A constant challenge with mode-of-action identification in *Plasmodium* sexual stages is their non-replicative state, which precludes traditional approaches such as long-term culture to determine the genetic-basis of antimalarial resistance (Luth et al., 2018). Thus, although this chemogenetic approach has been hugely valuable in mapping the druggable genome, it remains unattainable for studying sexual stage-targeted compounds. This demonstrates the power of CETSA, especially with the advancement of protocols specifically for *P. falciparum* drug-target identification (Dziekan et al., 2020). Combining CETSA with PAL, as we have shown here, will, we believe, accelerate further identification and validation of drug cellular targets for non-replicative-stage-targeting drugs.

In summary, we have identified Pfs16, described as an early marker of sexual conversion, as a candidate cellular target for the *N-*4HCS transmission-blocking compound scaffold. Further investment in both the compound and the protein target itself is now clearly warranted, with the structure of the protein and subsequent attempts at co-crystallisation being a key priority. With further development in the chemistry of transmission-blocking drugs and the exploration of avenues to either co-formulate with treatment drugs or deliver via alternative vector-targeting approaches, transmission-blocking drugs should strongly be considered as important components of future antimalarial combination therapies.

## MATERIALS AND METHODS

### *In vitro* culture of *Plasmodium falciparum* NF54 asexual blood stages and gametocytes

The *Plasmodium falciparum* NF54 strain [sourced from the Malaria Research and Reference Reagent Resource Center (https://www.beiresources.org/About/MR4.aspx) and regularly checked for contamination], the *PfDyn*GFP/*P47*mCherry strain [kindly gifted by Edwin Lasonder (Plymouth University) and Richard Bartfai and colleagues (Radboud University); Lasonder et al., 2016] and *Pf*2004/164-tdTom parasites (kindly gifted by Nicolas Brancucci and Matthias Marti, Harvard T.H. Chan School of Public Health; Brancucci et al., 2015) were cultured for asexual and sexual stage growth as previously described (Delves et al., 2016). In brief, asexual blood stage cultures were maintained at 0.75-5% parasitaemia and 4% haematocrit using O+ or A+ human erythrocytes [National Health Service (NHS) National Blood Service] supplemented with 30 units/ml heparin (Sigma-Aldrich). The culture medium was prepared from RPMI-1640 with 25 mM HEPES (Life Technologies) supplemented with 50 µg/ml hypoxanthine (Sigma-Aldrich), 2 g/l sodium bicarbonate and 10% A+ human serum (Interstate Blood Bank). The culture medium was changed daily and cultures were maintained at 37°C under 3% O_2_, 5% CO_2_ and 92% N_2_ (BOC Special Gases). For *Pf*2004/164-tdTom parasites, asexual parasites and gametocytes were cultured as described, but maintained at 5% haematocrit in medium supplemented with 4 nM WR 99210 (Jacobus Pharmaceutical).

Gametocytes were induced from asexual blood stage cultures at 3% parasitaemia and 4% haematocrit. Gametocytes were grown in RPMI-1640 with 25 mM HEPES supplemented with 2 mg/ml D-glucose, 150 µg/ml L-glutamine, 2.78 mg/ml sodium bicarbonate, 50 µg/ml hypoxanthine, 5% A+ human serum and 5% AlbuMAX II (Life Technologies). The gametocyte medium was changed daily without the addition of fresh erythrocytes for 14 days following induction, at which point stage V gametocytes were the most abundant. The functional viability of gametocytes was determined at day 14 post induction by measuring the percentage of exflagellation relative to the total erythrocyte density. The cultures were activated with ookinete medium [culture medium prepared as above supplemented with 100 µM xanthurenic acid (Sigma-Aldrich), lacking serum or AlbuMAX II] and exflagellation events were counted with a haemocytometer (VWR) using a Nikon Leica DC500 microscope.

### Compounds

DDD01035881 and DDD01028076 were purchased from Life Chemicals and maintained at 10 mM in DMSO (Honeywell). DDD01028076 was also synthesised by A.R.-Z. in house (Rueda-Zubiaurre et al., 2019).

For full methods on the synthesis of clickable derivatives based on the *N-*4HCS scaffold, see supplementary Materials and Methods. ML10 was kindly donated by the Baker laboratory, London School of Hygiene and Tropical Medicine (Baker et al., 2017), whereas BKI-1294 was kindly donated by the Van Voorhis laboratory, University of Washington (Ojo et al., 2014). Colchicine (Sigma-Aldrich) and cytochalasin B (Sigma-Aldrich) were commercially sourced. All compounds were made up to 10 mM stock solutions in DMSO and stored at −20°C.

### IC_50_ determination by male gamete formation assay

The male gamete formation assay was adapted from the previously described dual gamete formation assay (Ruecker et al., 2014) to identify male gamete-targeted activity. Briefly, compounds were dispensed into 384-well plates using the D300 Digital Dispenser (Hewlett-Packard) in serial dilutions. Single point controls, a DMSO negative control (0.25% final assay volume) and 20 µM DDD01028076 as a positive control were included across the plate. Maintaining room temperature (RT), 10 µl ookinete medium was dispensed into each well, followed by addition of 50 µl of stage V NF54 gametocyte culture (day 14 post induction and onwards). Gametocyte cultures, demonstrating ≥0.2% exflagellation, were diluted to 25 million erythrocytes/ml for the assays. The plates were then immediately incubated at 4°C for 4 min, followed by incubation at 28°C for 5 min.

Exflagellation events were recorded using a Nikon Eclipse Ti inverted microscope by automated data capture. Using the JOBS module of NIS elements (Nikon), a 10-frame time-lapse movie was captured at 4 fps under phase contrast at a ×4 objective, 1.5× zoom. Time-lapse movies were recorded at a *z*-coordinate determined from a prior autofocus step.

A semi-automated algorithm designed in Icy Bioimage Analysis software (de Chaumont et al., 2012) was used to obtain an exflagellation centre count based on the size, pixel intensity and circularity of disruptions in the erythrocyte monolayer of each time-lapse movie. Raw data with a Z′ factor ≥0.4 were normalised to positive and negative controls to calculate a percentage inhibition using the following equation:

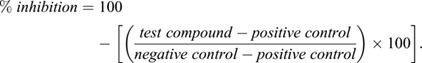


The IC_50_ of each compound was then determined using GraphPad Prism software.

### Activated gametocyte lysate preparation

Stage V *P. falciparum* NF54 gametocytes were purified by differential sedimentation using NycoPrep 1.077 (Axis-Shield Diagnostics) to remove the asexual parasite reservoir. Purified gametocytes were treated with ookinete medium to activate gametogenesis before halting the process at 2 min post activation at 4°C with 0.01% saponin (Sigma-Aldrich), used to lyse erythrocytes. Five saponin lysis steps were repeated at 4°C and the parasites were washed in PBS before snap freezing in liquid nitrogen and storing at −80°C. The pellets were used in lysate-labelling assays and CETSA.

### Target identification

#### Lysate labelling assays for IGF

##### Probe 2 treatment of cell lysates

For IGF detection of probe 2-treated lysates (Fig. S2A), 120 ml of untreated *P. falciparum* NF54 stage V gametocyte culture was purified using NycoPrep 1.077 and saponin lysis as above. The resulting cell pellet was lysed by gentle agitation in 1.2 ml lysis buffer [1% Triton X-100, 10 mM Tris, 150 mM NaCl, 1× cOmplete EDTA-free protease inhibitor (Roche Diagnostics)] for 30 min at 4°C. The protein concentration was determined using the DC protein assay (Bio-Rad), performed according to the manufacturer's instructions. The absorbance was measured at 750 nm and bovine serum albumin was used as a protein standard. Absorbance was measured using 96-well plates and a SpectraMax M2e Microplate Reader (Molecular Devices). Lysates were made up to 1 mg/ml in lysis buffer, transferred to microcentrifuge tubes and centrifuged (17,000 ***g***, 10 min, 4°C) to remove insoluble cellular debris. The lysates were then divided across six aliquots of 200 µl before treatment with probe 2 or DMSO, under conditions stated in Table S1. The samples were incubated at 4°C for 30 min and irradiated by UV light at 254 nm for 5 min.

##### CuAAC and protein precipitation

A click reaction master mix was prepared by combining the following reagents in order: (1) AzTB capture reagent (sourced by and synthesised as per Wright et al., 2015) (one volume of 10 mM DMSO stock; 0.1 mM final concentration), (2) CuSO_4_ (two volumes of 50 mM H_2_O stock; 1 mM final concentration), (3) tris(2-carboxyethyl)phosphine (TCEP; Sigma-Aldrich) (two volumes of 50 mM H_2_O; 1 mM final concentration) and (4) tris[(1-benzyl-4-triazolyl)methyl]amine (Sigma-Aldrich) (one volume of 10 mM DMSO stock; 0.1 mM final concentration). Then, 6 μl of this master mix was added per 100 μl protein sample, vortexed and incubated with moderate shaking for 1 h at RT. For negative click control samples, H_2_O was added in place of the CuSO_4_ catalyst. The reaction was quenched by addition of 5 mM EDTA (from a 500 mM stock in H_2_O). Proteins were precipitated by addition of methanol (four volumes), chloroform (one volume) and H_2_O (three volumes) for nLC-MS/MS, or methanol (two volumes), chloroform (0.5 volumes) and H_2_O (one volume) for gel-based analysis. The precipitated proteins were centrifuged at 17,000 ***g*** for 2 min at 4°C. The protein pellet was isolated by removal of the chloroform and methanol/H_2_O layers and washed with methanol (four volumes). The proteins were then sonicated and transferred to −80°C storage for a minimum of 20 min. The samples were centrifuged (17,000 ***g***, 5 min), methanol was removed and the resulting protein was air dried for 5 min at RT. The protein pellet was resolubilised in 2% SDS and sonicated until it was fully dissolved, before dilution to 0.2% SDS in 1× PBS.

##### Pull-down with Dynabeads (Streptavidin MyOne)

Probe 2-treated and AzTB-labelled cell lysate samples to be analysed by IGF were incubated with magnetic Dynabeads (Streptavidin MyOne) (Thermo Fisher Scientific). Beads were prewashed with three volumes of 0.2% SDS, with rotary mixing for 3 min at RT. The samples were added to the beads and moderately shaken for 2 h at RT. The supernatant was removed, retaining an aliquot for SDS-PAGE analysis. The beads were then washed with three volumes of 0.2% SDS. Enriched proteins were eluted by boiling with 2% (v/v) 2-mercaptoethanol-containing NuPAGE LDS sample loading buffer (Thermo Fisher Scientific) at 95°C for 5 min. The samples were separated by gel electrophoresis.

##### Gel electrophoresis and IGF

SDS-PAGE analysis was performed with 12% acrylamide Bis-Tris gels, using a Bio-Rad Mini-PROTEAN Tetra Cell system with MOPS running buffer (5 mM MOPS pH 7.7, 50 mM Tris base, 0.1% SDS, 1 mM EDTA), using Precision Plus Protein All Blue Standard (Bio-Rad) as a molecular mass marker. IGF was detected (excitation wavelength 552 nm, emission wavelength 570 nm) using a Typhoon FLA 9500 Imager (GE Healthcare). Further data analysis was performed with the ImageQuant software (GE Healthcare).

#### PAL for nLC-MS/MS

##### Probe 2 treatment of live *P. falciparum* gametocytes

For identification (Fig. 2B,C; Fig. S1B,C) and validation (Fig. 2C,D,E,G; Figs S2 and S3) of *N-*4HCS cellular targets by PAL, live *P. falciparum* NF54 stage V gametocyte cultures (≥0.3% exflagellation) were treated and irradiated by UV light, as opposed to the lysate-based treatment outlined above. For nLC-MS/MS, gametocytes were treated with either DMSO, probe 2 (10 µM) only or a combination of probe 2 (10 µM) and parent molecule 1 (10 µM). The DMSO concentration was normalised across all samples and samples were obtained in triplicate. Following treatment, parasites were incubated for 10 min at 37°C and subsequently irradiated with UV light at 254 nm for 10 min. The gametocytes were then purified using NycoPrep 1.077 and lysed with saponin to lyse erythrocytes. Lysis buffer (1% Triton X-100, 10 mM Tris, 150 mM NaCl, cOmplete ULTRA EDTA-free Protease Inhibitor Cocktail at pH 7.5 in H_2_O) was added to treated gametocyte pellets, and parasites were lysed by sonication (60% amplitude, 3 min; 2 s pulse, 2 s rest) and centrifuged (17,000 ***g***, 30 min, 4°C). The supernatant was retained, and probe 2-labelled proteins were ligated to AzTB by performing the CuAAC reaction as described above.

##### Pulldown for nLC-MS/MS

Samples being prepared for nLC-MS/MS analysis were incubated with NeutrAvidin agarose beads (Thermo Fisher Scientific), which produce a low background signal. For bead derivatisation, the beads were washed five times with triethylammonium bicarbonate (TEAB, 100 mM, pH 8). The beads were then gently agitated for 1 h at RT in a solution of 100 mM TEAB, 25 mM NaBH_3_CN and 0.2% formaldehyde. The reaction was quenched by washing twice with 1% ethanolamine in 100 mM TEAB before subsequently washing three times with HEPES (50 mM, pH 8). Before the addition of protein samples, the derivatised beads were washed twice with 0.2% SDS in HEPES (50 mM, pH 8). Air-dried protein samples were dissolved in 0.2% SDS in HEPES (50 mM, pH 8), added to the beads and shaken for 2 h at RT. Following incubation, the beads were recovered and the supernatant was removed. The beads were washed twice with 0.2% SDS in HEPES (50 mM, pH 8) and washed a further four times with HEPES (50 mM, pH 8).

##### LysC and trypsin digestion

To elute protein from beads, LysC (in 50 mM HEPES, pH 8) (Promega) was added to the samples and incubated for 1 h at 37°C, using 2 µl LysC per 30 µl of derivatised beads. The beads were pelleted and 50 µl of the supernatant of the samples was retained and combined. The beads were washed with 50 µl HEPES (50 mM, pH 8). TCEP (5 mM) and chloroacetamide (10 mM; Sigma-Aldrich) were added to the combined supernatants and gently agitated for 10 min at RT. Trypsin (0.5 µl of 20 µg/100 µl in HEPES 50 mM, pH 8.3; Promega) was added to each sample and incubated overnight at 37°C.

##### 9-plex TMT labelling

TMT reagents (Thermo Fisher Scientific) were prepared in acetonitrile and added to an equal volume of the sample before incubating with moderate shaking for 2 h at RT. Each reaction was quenched with 1 µl 5% hydroxylamine (Sigma-Aldrich) before combining all the samples into one tube. The sample was dried by centrifugal evaporation at 45°C.

##### Desalting and three-layer fractionation

All fractionation centrifugations were performed at 1100 ***g*** for 2 min at RT. The samples were resuspended in 150 µl of 1% (v/v) trifluoroacetic acid (TFA; Sigma-Aldrich)/H_2_O and 90% of the sample volume was transferred to a stage tip containing polystyrene-divinylbenzene copolymer modified with sulphonic acid (SDB-RPS, Supelco) and centrifuged at 3000 ***g*** for 3 min. Columns were desalted by washing with 0.2% TFA (60 µl) before elution into separate tubes, with the sequential addition of three buffers (Table S2). The samples were evaporated to dryness in a Savant SPD1010 SpeedVac Concentrator (Thermo Fisher Scientific) at 45°C. Prior to separation and analysis by Q Exactive LC-MS, the dried fractionation samples were resuspended in 2% acetonitrile (Sigma-Aldrich), 0.5% TFA in H_2_O (LC-MS grade) by gentle agitation and sonication, to give a final concentration of ∼1 µg/µl. A stage tip filter was prepared containing three layers of PVDF Durapore filter (0.1 µm; Millipore). The samples (12 µl) were transferred to stage tips and centrifuged into LC-MS vials at 4000 ***g*** for 5 min at RT.

##### nLC-MS/MS data acquisition

The peptides were separated on an Acclaim PepMap RSLC column (50 cm×75 µm inner diameter, Thermo Fisher Scientific) using a 3-h acetonitrile gradient in 0.1% aqueous formic acid at a flow rate of 250 nl/min. The EASY nLC-1000 liquid chromatograph was coupled to a Q Exactive mass spectrometer via an easy-spray source (Thermo Fisher Scientific). The Q Exactive mass spectrometer was operated in data-dependent mode with survey scans acquired at a resolution of 70,000 at a mass-to-charge ration (m/z) of 200. Scans were acquired from 350 to 1800 m/z. Up to ten of the most abundant isotope patterns (a minimum of charge 2) from the survey scan were selected with an isolation window of 1.6 m/z and fragmented by higher-energy collision dissociation with a normalised collision energy of 31 W. The maximum ion injection times for the survey scan and the MS/MS scans (acquired with a resolution of 35,000 at m/z 200) were 20 and 120 ms, respectively. The ion target value for MS was set to 106 and for MS/MS to 2×105, and the intensity threshold was set to 1.7×103.

##### Protein database search and TMT-labelling quantification

Raw files were uploaded into MaxQuant (version 1.6.1.0) (Cox and Mann, 2008) and searched against the curated UniProt *P. falciparum* NF54 proteome (UniProt, Feb 2018, 8637 entries) (UniProt Consortium, 2019) using the built-in Andromeda search engine. Cysteine carbamidomethylation was selected as a fixed modification, and methionine oxidation and acetylation of the protein N-terminus as variable modifications. For *in silico* digests of the reference proteome, the following peptide bond cleavages were allowed: arginine or lysine followed by any amino acid (a general setting referred to as ‘Trypsin/P’). Up to two missed cleavages were allowed. The false discovery rate was set to 0.01 for peptides, proteins and sites. Other parameters were used as pre-set in the software (maximal mass error 4.5 ppm and 20 ppm for precursor and product ions, respectively; minimum peptide length=7; minimum razor unique peptides=2; minimum scores for unmodified and modified peptides=0 and 40, respectively). The ‘match between runs’ option (time window 0.7 min) was allowed and the ‘unique and razor peptides’ mode was selected to allow identification and quantification of proteins in groups (razor peptides are uniquely assigned to protein groups and not to individual proteins), and for TMT quantification (MS/MS mode), the minimal ratio count of 2 was selected.

TMT labelling efficiency analysis was performed using MaxQuant (v.2.1.0.0) and calculations were done using Perseus (v2.0.5.0). TMT labels were searched at both lysines and N-termini, and labelling efficiency was calculated separately for two sets of replicates and averaged for each experiment (refer to Fig. S2B).

##### Proteomics data analysis

Data analysis was performed using Perseus (v2.0.5.0) (Kulak et al., 2014). Corrected reporter intensity values were filtered to remove rows based on ‘contaminants’ and ‘reverse’ columns. The data was log_2_ transformed and the median values within each column (TMT channel) subtracted. Protein groups with at least two valid values were retained. An unpaired two-sample two-tailed *t*-test (permutation-based false discovery rate=0.10; variance parameter S0=0.15) was applied to all proteins in the dataset and results analysed according to their statistical significance. The PlasmoDB database (https://plasmodb.org/) was used to analyse the identified proteins.

### Target validation

#### PAL analysed by IGF and western blotting

##### Treatment and CuAAC

For IGF and western blot analysis (Fig. 2D; Figs S2 and S3), live *P. falciparum* NF54 gametocytes were treated with either DMSO or probe 2 (2.5 µM, 5 µM or 10 µM) and irradiated as described above. Gametocytes were purified with Nycoprep 1.077 and lysed with saponin to obtain a parasite pellet. The pellets were lysed by sonication (60% amplitude, 3 min; 2 s pulse, 2 s rest) and centrifuged (17,000 ***g***, 30 min, 4°C) in lysis buffer (1% Triton X-100, 10 mM Tris, 150 mM NaCl, cOmplete ULTRA EDTA-free Protease Inhibitor Cocktail at pH 7.5 in H_2_O).

The protein concentration was determined using the Pierce BCA Protein Assay Kit (Thermo Fisher Scientific) following the manufacturer's instructions. The absorbance was measured using a NanoDrop 2000 spectrophotometer (Thermo Fisher Scientific), using bovine serum albumin as a protein standard. The lysed protein was made up to 0.5-1 mg/ml in PBS to a volume of 100 µl to perform the CuAAC reaction and protein precipitation, as described above. Samples of the clicked sample and crude lysate, with a total of 10 µg protein each, were set aside to be analysed by IGF and western blotting.

##### Pulldown and IGF

Following CuAAC, protein samples were incubated with Pierce streptavidin-coated magnetic beads (Thermo Fisher Scientific), using 300 µl beads per 1 mg of total protein. The beads were washed three times by moderately shaking with 0.2% SDS in PBS and partitioning with a magnet. Protein samples were added to the washed beads and incubated at RT for 2 h, with moderate shaking. The flow-through was retained for analysis by partitioning with a magnet and the beads were then washed three times with 0.2% SDS. The beads were washed once more with 0.1% Tween-20 in H_2_O before the addition of 0.1 M glycine, pH 2.0 and moderate shaking for 5 min at RT. Enriched proteins were then boiled with 2× sample loading buffer for 10 min at 95°C before centrifugation (17,000 ***g***, 10 min at RT). The resulting eluate was loaded directly onto an SDS-PAGE gel, alongside the flow-through, crude lysate and clicked samples.

Proteins were separated on NuPage 4-12% Bis-Tris gels (Novex; Thermo Fisher Scientific) and AzTB-TAMRA IGF was detected with a FLA 5000 biomolecular imager (FujiFilm). Gels were further analysed by western blotting using standard methods, developed using ECL (Amersham). Pfs16 was detected with mouse anti-Pfs16 clone 32F717:B02 (1:800, provided by and produced as per Moelans, 1995), kindly donated by Robert Sauerwein and colleagues (Radboud University). Pfg377 was detected with rabbit polyclonal serum (1:2000) (Ishino et al., 2020), kindly donated by Tomoko Ishino and colleagues (Ehime University). Secondary antibodies [horseradish peroxidase-coupled goat anti-rabbit or goat anti-mouse (STAR121P and STAR120P, Bio-Rad) were used at a 1:10,000 dilution.

##### CETSA

The melt curve and ITDR-CETSA protocols were adapted from previously described protocols (Jafari et al., 2014). An immunoblot-based approach was carried out with activated gametocyte or mixed stage I-III/asexual parasite lysates. The soluble protein fraction of parasite pellets was obtained by addition of lysis buffer (1% Triton X-100, 10 mM Tris, 150 mM NaCl, cOmplete ULTRA EDTA-free Protease Inhibitor Cocktail at pH 7.5 in H_2_O) and centrifugation at 17,000 ***g*** for 20 min at 4°C.

To obtain a melt curve for T_m_ extrapolation, the soluble protein fraction was treated with 1% DMSO or 100 µM DDD01035881. 10 µl aliquots of the protein fractions were aliquoted into PCR tubes and incubated at RT for 3 min. The fractions were then thermally challenged over a temperature gradient from either 51.6, 76.6, 61.9 or 71.5 to 100°C for 5 min using the Bio-Rad C1000 Touch Thermal Cycler and incubated for a further 3 min at 4°C. To remove the aggregated proteins, the soluble fraction of the heat-treated parasites was obtained by centrifugation at 17,000 ***g*** for 45 min. The stabilised proteins were then prepared for visualisation by immunoblotting.

The ITDR format of CETSA was performed over a range of concentrations and single temperature of 78.4°C. The temperature was derived from the melt curve as the temperature at which Pfs16 had mostly aggregated under DMSO treatment but was stabilised by DDD01035881. DDD01035881 was dispensed into 384-well plates using the D300 Digital Dispenser from 1 nM to 100 µM. Then, 10 µl soluble protein, prepared as described above, was added and incubated with DDD01035881 for 10 min. The treated protein was thermally challenged at 78.4°C for 5 min and incubated at 4°C for 3 min. The stabilised proteins were then isolated from these fractions by centrifugation at 17,000 ***g*** for 45 min and prepared for immunoblot analysis.

Gel electrophoresis and detection of Pfs16 and Pfg377 by immunoblotting was performed as described above. Densitometry analysis was carried out using ImageJ and data were normalised to the lowest and highest values to obtain relative band intensities. The normalised data were analysed to obtain melt curves using the Boltzmann sigmoid equation in GraphPad Prism; these values can be found in Table S4. ITDR data were fitted using the saturation binding curve (one site binding rectangular hyperbola) function in GraphPad Prism.

##### Flow cytometry-based measurement of sexual conversion

The sexual conversion rates of *Pf*2004/164-tdTomato parasites were determined as previously described (Brancucci et al., 2015). Asexual parasites with >2% ring-stage parasitaemia were synchronised with 5% sorbitol to bring parasites to a window of 0-24 h post invasion. Parasites were brought to an 8-h window (16 to 24 h post invasion) by repeating the synchronisation within the same intraerythrocytic developmental cycle. The synchronised parasites were washed with fresh medium, diluted to 2.5% haematocrit, and 220 µl of dilute cell suspension was plated into the wells of a 96-well plate for induction of sexual conversion.

Next, 25 µM of DDD01035881 and DMSO controls were included across the plate; all wells were normalised to 0.25% DMSO. For carryover samples, the compounds were plated prior to induction. For post-induction-treated samples, the compounds were plated 24 h after initial plating and induction. For compound-washout samples, the parasites were plated onto compound-treated plates and washed three times at 24 h post induction. Parasite controls were included by washing and plating synchronised parasites with medium treated with 20 µM choline (Sigma-Aldrich) to block sexual conversion.

Forty-eight hours after induction, the parasitaemia of samples was determined by staining 10 µl of each sample with SYBR Green (1:5000 in PBS) (Bio-Rad). Cells were stained for 20 min at 37°C and washed twice before measuring SYBR Green-positive cells as a measure of parasitaemia by flow cytometry using a BD Fortessa flow cytometer. SYBR Green was detected with the 488 nm laser using the 530/30 filter and tdTomato with the 561 nm laser using the 586/16 filter. 100,000 events were acquired per sample. Forty-eight hours after counting parasitaemia, SYBR Green staining was repeated to measure gametocytaemia by flow cytometry. SYBR Green and tdTomato double-positive cells represented gametocyte populations. 400,000 events were acquired per sample.

The sexual conversion rate of each sample was determined with the following equation:

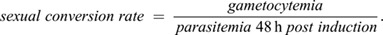


Rates are provided in Table S4.

#### Relative exflagellation counts

For incremental compound treatment post gametogenesis, five parts of stage V NF54 gametocytes (day 14 post induction and onwards) were activated with one part ookinete medium at RT. The cells were treated with varying concentrations of DDD01035881, ML10 and 1294 at several time points post activation. The viability was then determined by counting exflagellation events or by analysing cells by imaging. For viability measurements, exflagellation rates relative to erythrocyte density were determined at 25 min post activation using a haemocytometer. For immunofluorescence labelling, cells were fixed at 25 min post activation and stained as described above.

To determine the reversibility of DDD01035881, gametocytes were activated as described above and the compound was removed with three washes at 1 or 6 min post activation. The viability was determined by counting exflagellation rates at 25 min post activation. Exflagellation rates of DDD01035881-treated gametocytes were determined relative to DMSO controls. The relative rates of DDD01035881-treated parasites that were washed at 1 or 6 min were calculated relative to DMSO controls that were washed three times at 1 min post activation, accounting for reductions in exflagellation owing to centrifugation. All rates are listed in Table S4.

##### Flow cytometry measurements of microgametocyte ploidy

The extent of DNA replication of *PfDyn*GFP/*P47*mCherry gametocytes with and without DDD01035881 treatment was determined as previously described (Fang et al., 2017). Stage V *PfDyn*GFP/*P47*mCherry and NF54 gametocytes were purified with NycoPrep 1.077. Purified gametocytes were resuspended in suspended activation medium [RPMI-1640 with 25 mM HEPES (Life Technologies), 4 mM sodium bicarbonate and 5% fetal bovine serum (Thermo Fisher Scientific), pH 7.2] to permit staining at RT without premature activation of gametogenesis. For T=15 min samples, the parasites were activated with ookinete medium and gametogenesis was halted with ice-cold PBS at 15 min. For T=0 min samples, the parasites were immediately resuspended in ice-cold PBS. All samples were washed at 300 ***g*** for 2 min at 4°C, resuspended in ice-cold PBS and stained with 1:2000 Vybrant DyeCycle Violet (Thermo Fisher Scientific) for 30 min at 4°C. Stained and unstained erythrocyte and NF54 (T=0 and T=15 min) controls were prepared. The DNA content was measured as Vybrant DyeCycle Violet intensity by flow cytometry and data were analysed using FlowJo software (BD Life Sciences). GFP-positive male gametocytes were gated and ploidy was measured and expressed as a percentage of the total male population. The 530/30, 610/10 and 450/50 filters were used to analyse GFP, mCherry and Vybrant DyeCycle Violet, respectively. 100,000 events were analysed per sample. Population values are listed in Table S4.

##### Immunofluorescence staining and imaging

Mature NF54 gametocyte cultures were treated with either DMSO or test compounds. Gametocytes were treated with 5 µM DDD01035881, 1 µM 1294 and 25 µM ML10, before immediately activating without prior incubation. Gametocytes were treated with 50 µM colchicine and cytochalasin B for 48 h. For T=0 min samples, the parasites were immediately fixed in prewarmed 4% paraformaldehyde. The cultures were activated by xanthurenic acid-containing ookinete medium and fixed at several time points post activation. All fixed samples were adhered to poly-L-lysine (Sigma-Aldrich)-coated glass coverslips, before cells were washed once in PBS, permeabilised in 0.1% Triton X-100, washed thrice more in PBS and blocked with 10% fetal bovine serum. Cells were labelled with the following primary antibodies for 1 h: mouse anti-α tubulin clone DM1A (1:500, Sigma-Aldrich, CP06), rabbit anti-glycophorin A clone EPR8200 (1:1000, Abcam, ab129024) and mouse anti-Pfs16 clone 32F717:B02 (1:800, provided by and produced as per Moelans, 1995) (a kind gift from Robert Sauerwein, Radboud University Medical Centre). Cells were labelled with secondary antibodies and other stains for 45 min: anti-mouse or anti-rabbit Alexa Fluor 488 (1:500, Thermo Fisher Scientific, A-11001 and A-11008, respectively), anti-mouse or anti-rabbit Alexa Fluor 594 (1:500, Thermo Fisher Scientific, A-11005 and A-11012, respectively), 5 µg/ml WGA Alexa Fluor 633 (Thermo Fisher Scientific, W21404) and 10 nM 4′,6-diamidino-2-phenylindole (DAPI). VectaShield mountant (Vector Laboratories) was used to mount coverslips onto glass slides. Images were acquired with a Nikon Ti-E inverted widefield microscope at 100× objective in 0.2 µm increments through *z*, using NIS Elements v4.20. *Z*-stack images were deconvolved using the EpiDemic plugin (Soulez et al., 2012) and compressed to maximum-intensity projections in Icy Bioimage Analysis software.

##### Electron microscopy

Stage V NF54 gametocytes were purified by density-barrier isolation with NycoPrep 1.077. Purified gametocytes were treated with either DMSO or 5 µM DDD01035881 prior to activation with ookinete medium. Parasites were fixed at 25 min post activation with 4% high EM grade paraformaldehyde, 2.5% v/v glutaraldehyde and 0.1% tannic acid in 0.1 M sodium cacodylate buffer, pH 7.2, for 3 h at RT and washed three times in ice-cold 0.1 M sodium cacodylate buffer at 20 min intervals. The cells were treated with 1% w/v osmium tetroxide in 0.1 M sodium cacodylate for 2 h at RT, washed with 0.1 M sodium cacodylate and stained with 1% w/v aqueous uranyl acetate for 1 h at RT. Samples were then dehydrated in an ethanol series and embedded in epoxy resin (TAAB). Then, 70 nm sections were cut using a Leica EM UC7 ultramicrotome, contrasted with Uranyless (TAAB) for 2 min and with 3% Reynolds lead citrate (TAAB) for 1 min according to the manufacturer's protocols. Sections were imaged on a JEOL JEM-1400Plus transmission electron microscope (120 kV) with a Ruby Camera (2 K×2 K).

### Ethical approval

Research-grade red blood cells (RBC) were purchased from the National Health Service (NHS) Blood and Transplant Service (NHS-BTS). These RBCs are given anonymously with no clinical history. Sourcing of RBCs in this manner, including informed consent are entirely handled by NHS-BTS. The Imperial College London Research Ethics Committee determined that this does not represent human subject research, so no independent ethical approval was required.

## Supplementary Material

10.1242/dmm.049950_sup1 Supplementary information
