# Supplementary material for: A novel class of sulphonamides potently block malaria transmission by targeting a Plasmodium vacuole membrane protein
Source: Dis Model Mech. 2023 Jan 30;16(2):dmm049950. doi: 10.1242/dmm.049950 (PMC9934914; doi:10.1242/dmm.049950)
Supplement: Supplementary information [file dmm-16-049950-s1.pdf]

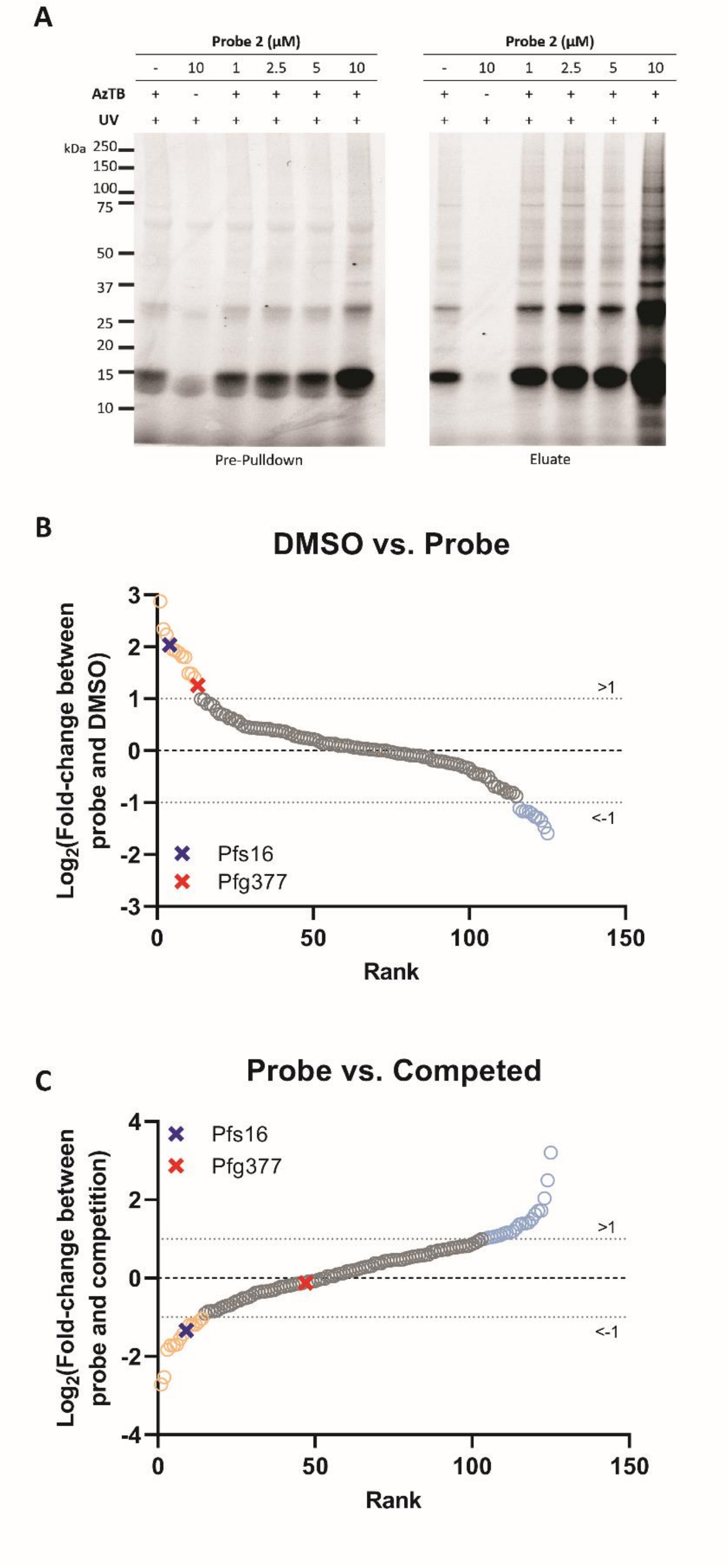

Fig. S1. (A) Confirmation of protein pulldown in the presence of AzTB capture reagent and probe **2** using cell lysate derived from *P. falciparum* gametocyte culture. Results confirm an AzTB-specific and probe **2** concentration-dependent pulldown by IGF. **(B-C)** Proteome-wide results from live treatment of *P. falciparum* stage V gametocyte analysed in a 9plex

TMT study. Plots depict the log_2-transformed fold change in protein enrichment between the of **(B)** DMSO and probe **2** treated samples or **(C)** probe **2** and competition (combined treatment probe **2** and parent molecule **1**) samples. All protein hits, represented as circles, are ranked based on the log_2-transformed fold change in enrichment, between the average values of samples. Averages were taken of 3 distinct biological replicates. Orange circles denote positive log-transformed fold change >1 and blue circles denote negative log-transformed fold change <-1 in **B**, and vice-versa in **C**. Pfs16 and Pfg377 are marked with blue and red circles, respectively, in both **B** and **C**.

Disease Models & Mechanisms • Supplementary information

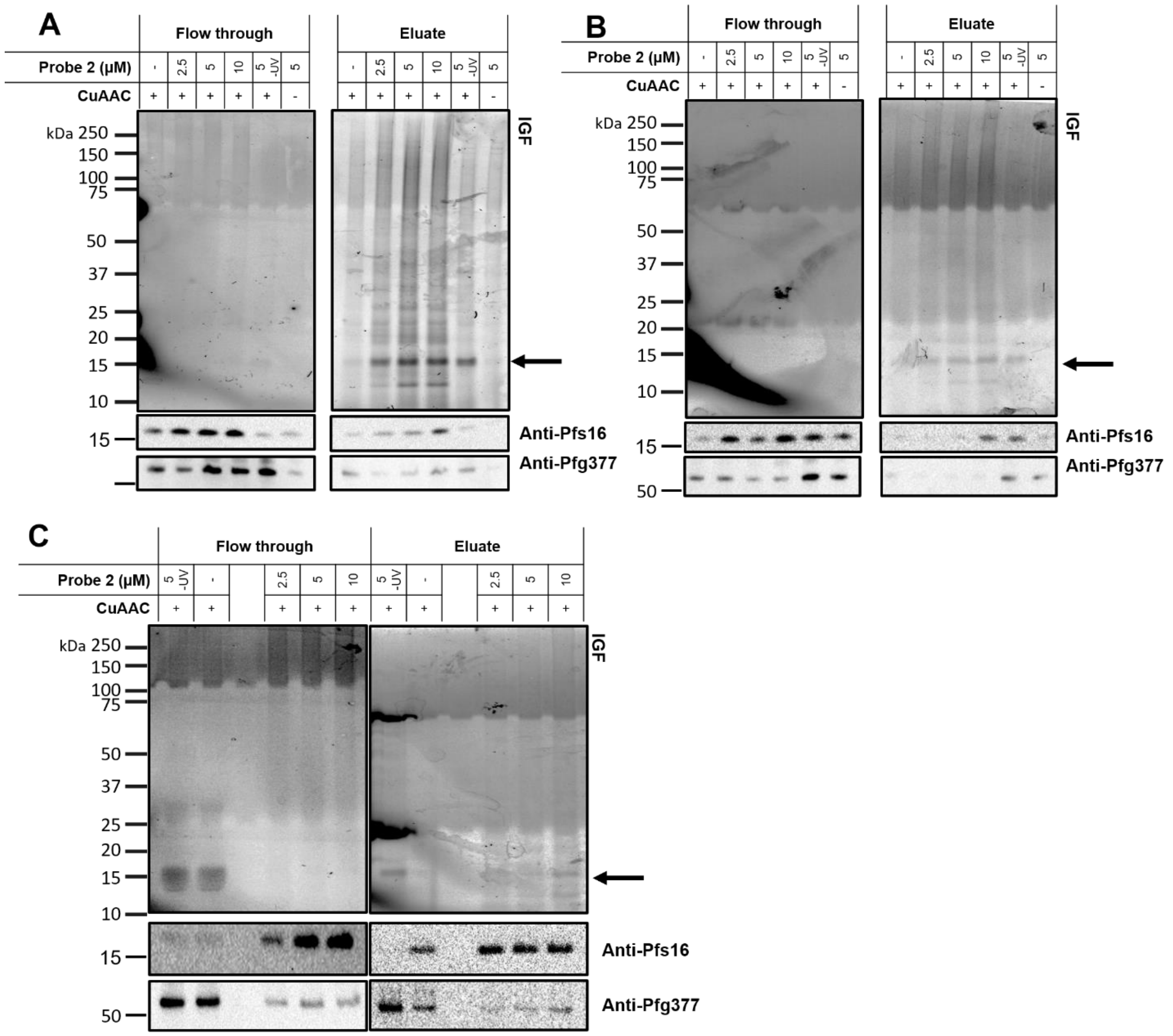

Fig. S2. Live parasite PAL full IGF and corresponding immunoblot data

(A-C) Replicates of raw data acquired from treatment of live gametocytes with increasing probe **2** doses prior to cell lysis, ligation to AzTB and streptavidin-enrichment of labelled proteins. The full IGF and corresponding immunoblots against Pfs16 and Pfg377 depict the flow through and enriched eluate samples from pulldowns.15-20kDa bands of the IGF gels, likely corresponding to Pfs16, and Pfs16 and Pfg377 labelled in immunoblots were analysed by densitometry. CuAAC denotes performance of the click reaction, samples denoted with a – represent click controls lacking a copper catalyst. -UV samples represent UV controls which were not irradiated with UV after probe **2** treatment.

Disease Models & Mechanisms • Supplementary information

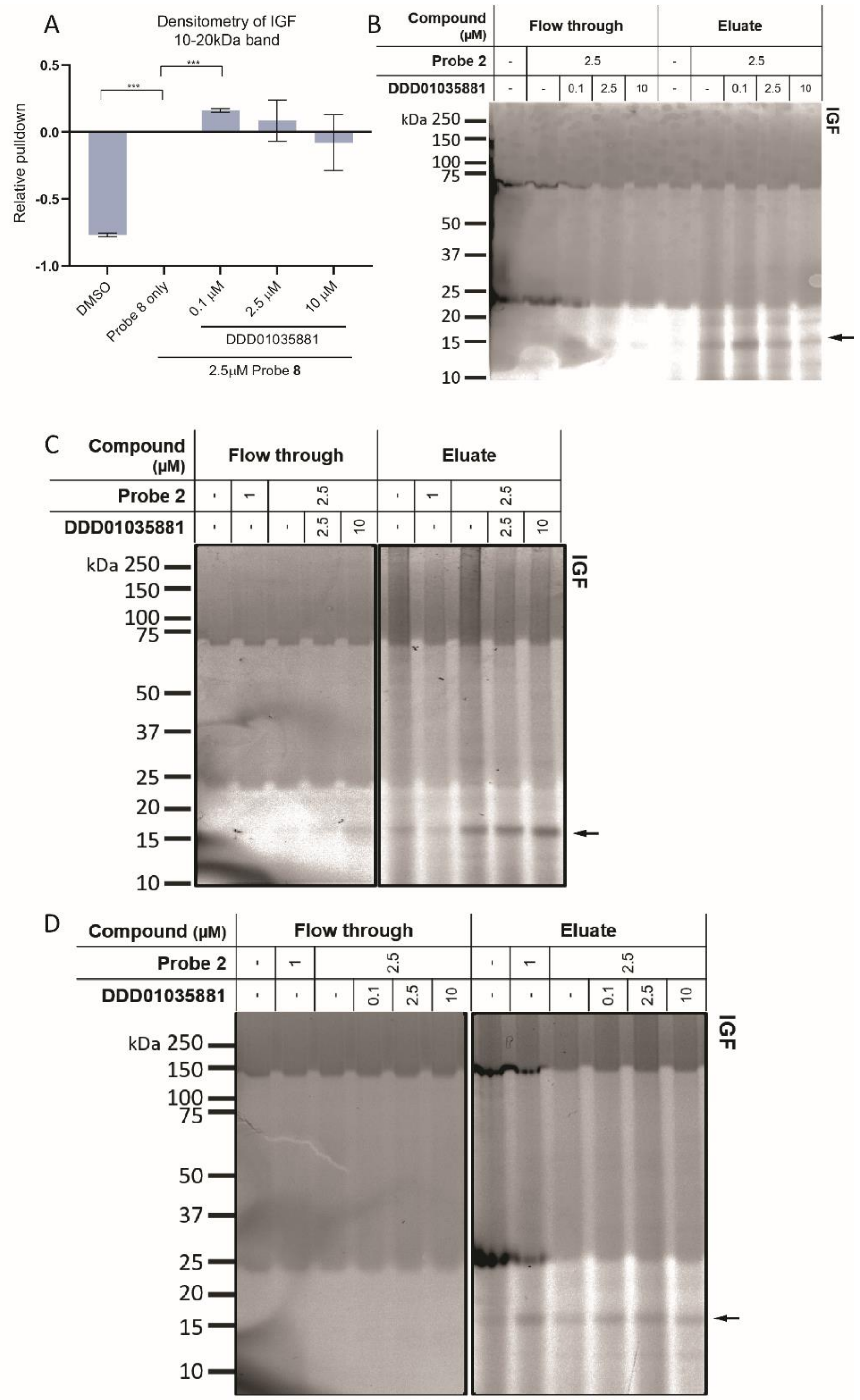

Fig. S3. IGF and corresponding densitometry of live parasite PAL performed in competition with DDD01035881

Target validation by live treatment with probe **2** and competitor, AzTB conjugation and streptavidin-biotin affinity enrichment. Increasing concentrations of competitor were incubated with a single concentration of probe **2**. **(A)** Densitometry of the 15-20kDa protein band, likely corresponding to Pfs16, in the streptavidin enriched fractions depicted as

Disease Models & Mechanisms • Supplementary information

relative band intensity, relative to a DMSO control. Error bars denote SEM of 2-3 biological replicates. Significance in unpaired two-tailed t-test denoted as *** ($p < 0.001$). **(B-D)** Replicates of raw IGF data analysed for **A** with parent compounds **DDD01035881**. The 16kDa protein band analysed by densitometry is labelled with an arrow.

Disease Models & Mechanisms • Supplementary information

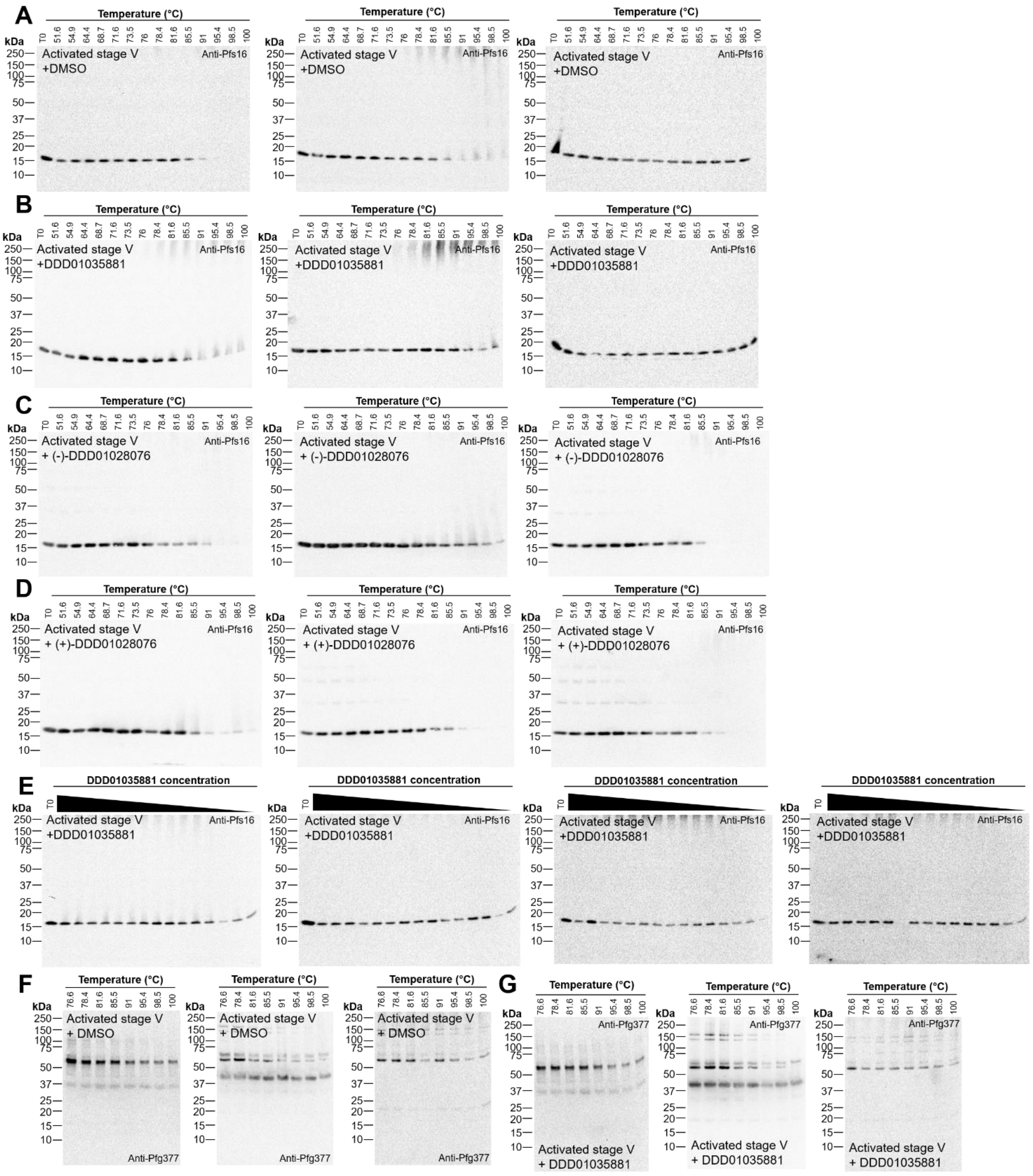

Fig. S4. CETSA stage V gametocyte lysate immunoblots

Full immunoblots obtained for densitometric analysis using activated stage V gametocyte lysate, treated with **(A)** DMSO, **(B) DDD01035881**, **(C) (-)-DDD01028076** or **(D) (+)-DDD01028076**, thermally challenged from 51.6-100°C and labelled with anti-Pfs16 antibody. **(E)** Anti-Pfs16 labelled immunoblots depicting the ITDR **DDD01035881**-treatment of activated stage V gametocyte lysate from 100µM-1nM and thermally challenged at 78.4°C. T0 controls represent

Disease Models & Mechanisms • Supplementary information

stage V gametocyte lysate. **(F-G)** Full immunoblots of activated stage V gametocyte lysate protein control labelled with anti-Pfg377, treated with **(F)** DMSO or **(G) DDD01035881** and thermally challenged from 76.6-100°C.

Disease Models & Mechanisms • Supplementary information

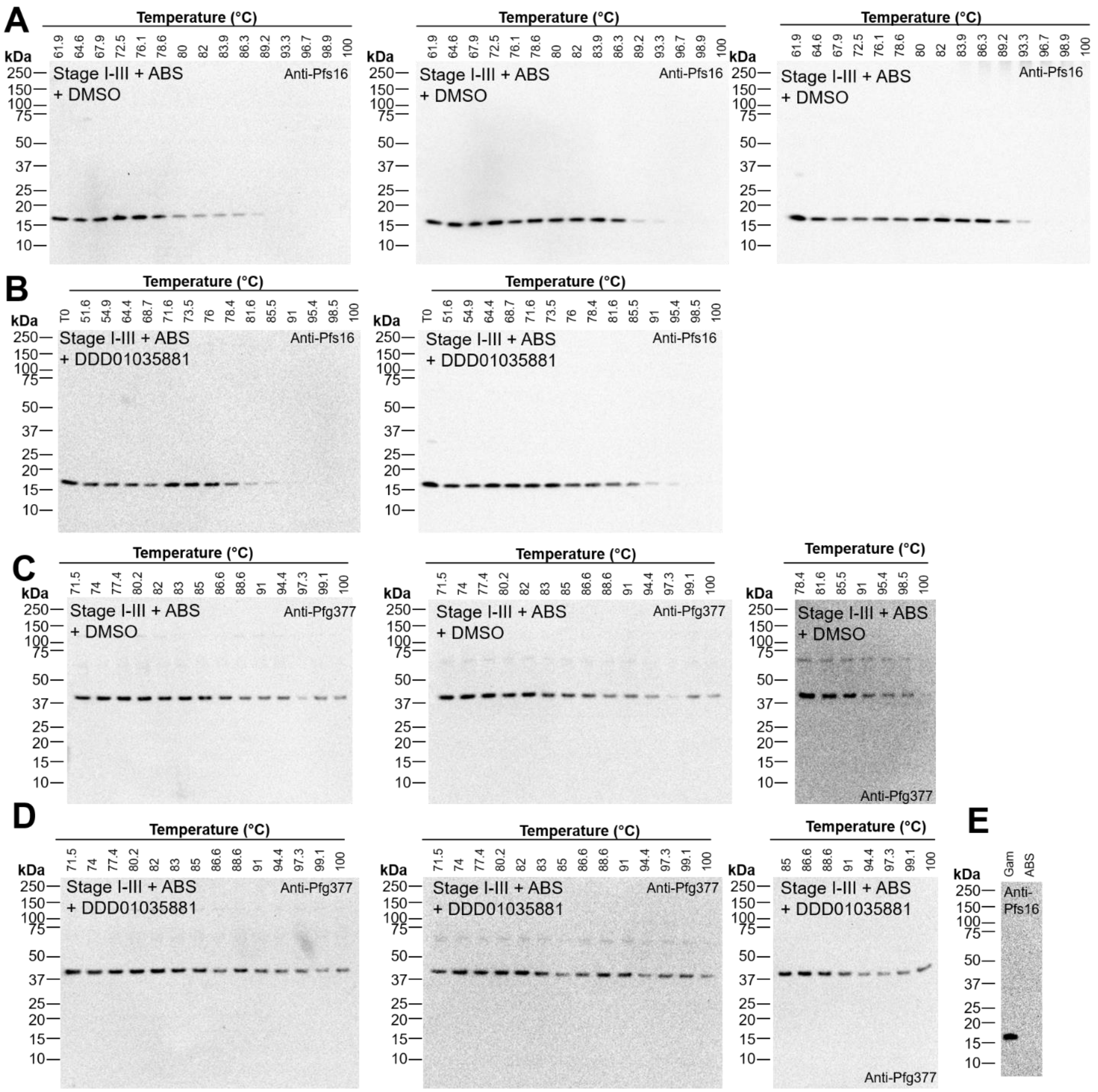

Fig. S5. CETSA early gametocyte and asexual parasite lysate immunoblots

Full immunoblots obtained for densitometric analysis using mixed ABS and stage I-III gametocyte lysate, treated with **(A)** DMSO or **(B) DDD01035881**, thermally challenged from 51.6-100°C and labelled with anti-Pfs16 antibody. Full blots of protein control labelled with anti-Pfg377, treated with **(C)** DMSO or **(D) DDD01035881** and thermally challenged from 71.5-100°C. Note, probing with anti-Pfg377 in activated stage V gametocytes contains an additional uncharacterised band by immunoblot, present at 60 kDa, which is only faintly recognised in ABS or stage I-III gametocytes. **(E)** Full immunoblot demonstrating the gametocyte specific Pfs16 expression, permitting the production of mixed ABS and stage I-III gametocyte lysate used in **A-D** without gametocyte purification.

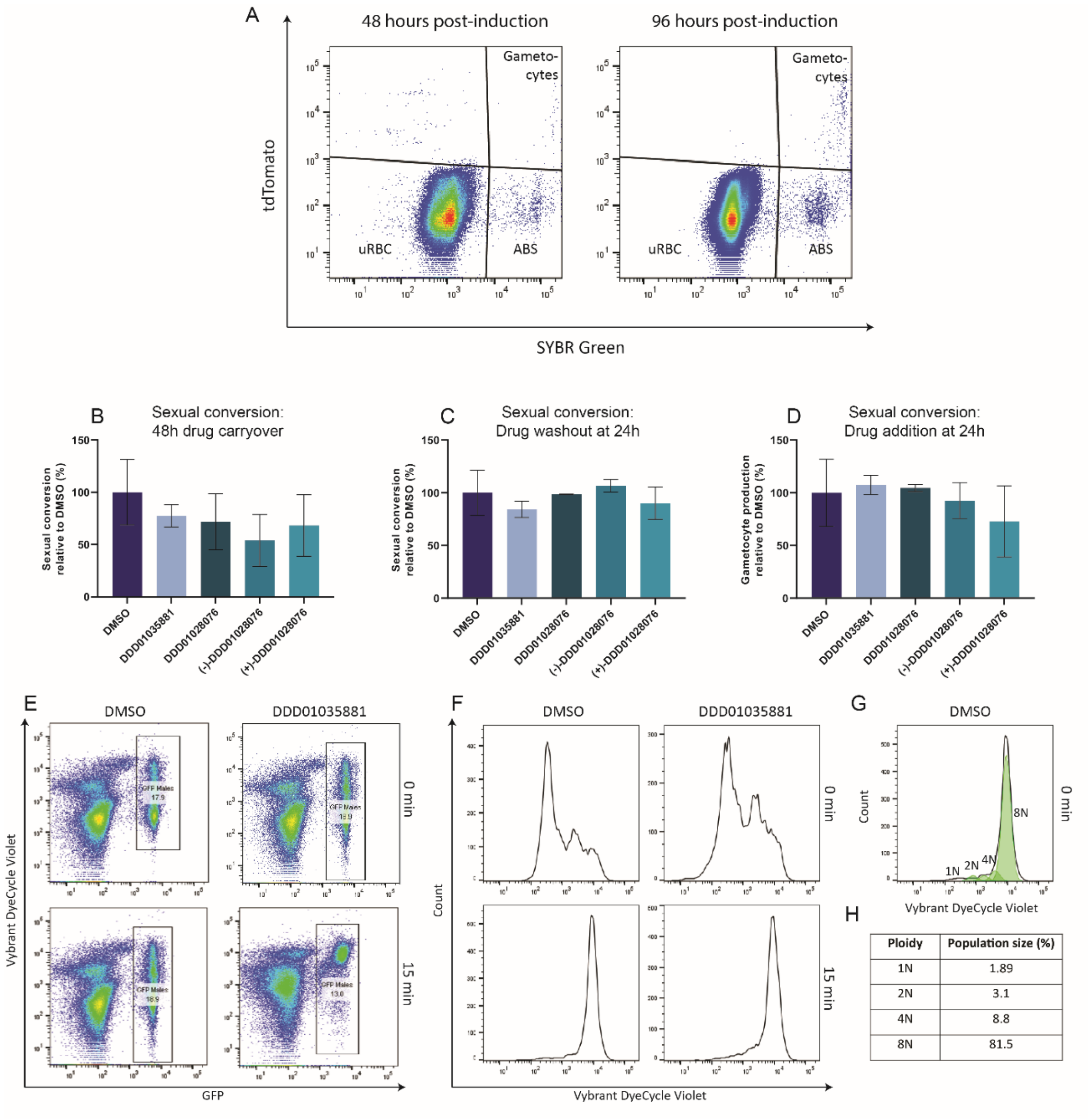

Fig. S6. Flow cytometry gating strategies and N-4HCS analogue results

(A) Flow cytometry of SYBR Green stained *Pf*2004/164-tdTomato gating strategy. Sexual conversion rates of *Pf*2004/164-tdTomato parasites were quantified as the tdTomato and SYBR Green positive gametocytes at 96 hours post-induction relative to the parasitemia at 48 hours post-induction, measured as the SYBR Green positive parasite population. Perturbation effects were then quantified relative to DMSO controls. **(B-D)** Corresponding sexual conversion rates of Pf2004/164-tdTomato parasites of **DDD01035881**, **DDD01028076**, **(-)-DDD01028076** and **(+)-DDD01028076**-treated parasites are expressed as rates relative to the conversion rates of DMSO-treated parasites. Perturbations to sexual conversion were determined by **(B)** maintaining treatment over two intraerythrocytic cycles and **(C)** the reversibility of any perturbations were determined by removing compound at 24 hours. **(D)** Perturbations to early gametocyte development were probed by administration of compounds in a subsequent intraerythrocytic cycle. Error bars represent the SEM of 2 biological replicates. **(E-H)** Perturbations to ploidy during

microgametogenesis were quantified by flow cytometry of Vybrant™ DyeCycle™ Violet stained *PfDyn*GFP/*Pf47*mCherry. Ploidy was measured by **(E)** gating GFP-positive male gametocytes from the 2 conditions of DMSO or **DDD01035881** treatment at 0 and 15-minutes post-activation. **(F)** Histograms of Vybrant™ DyeCycle™ Violet staining of males gated from **(E)** were further gated into 1N, 2N, 4N and 8N populations using the **(G)** proliferation model in FlowJo, before dividing into **(H)** relative populations.

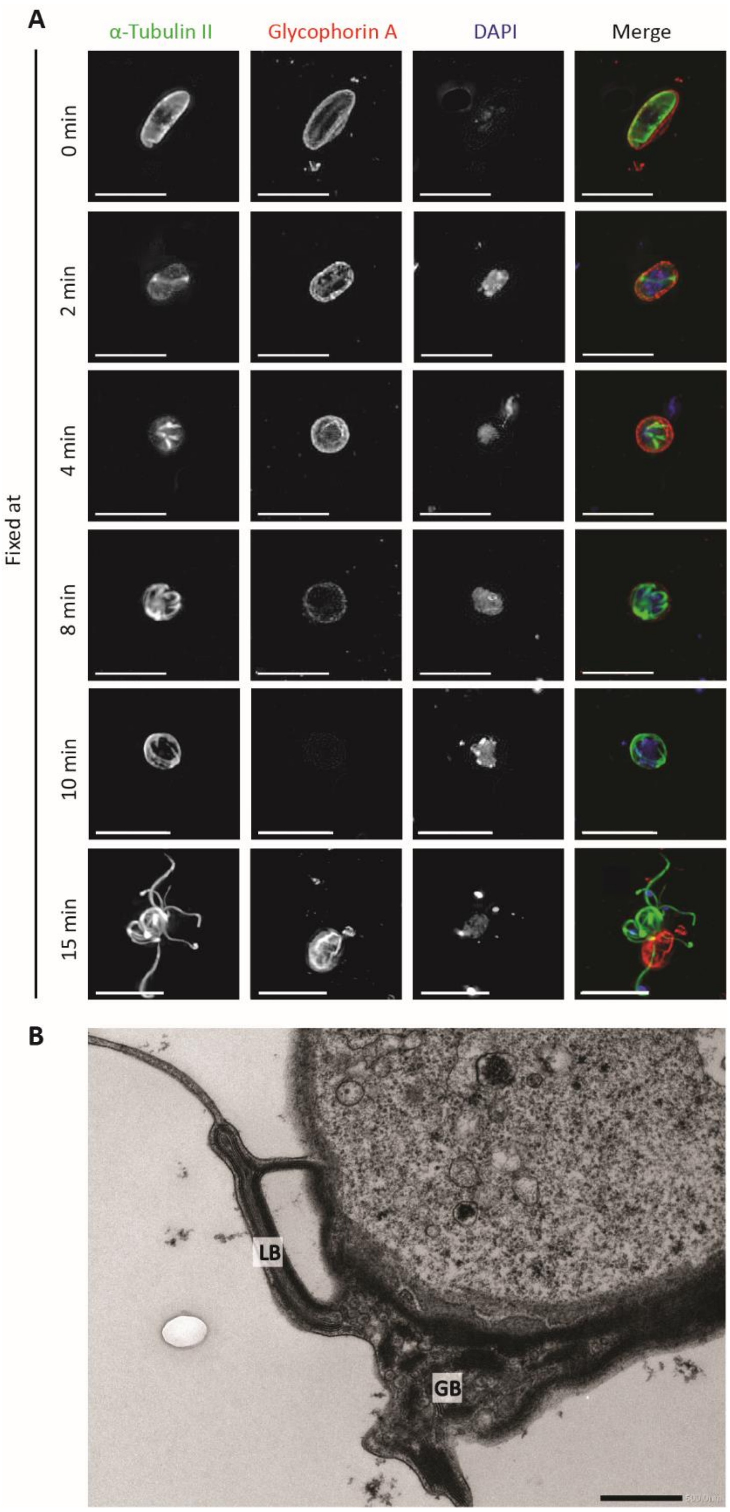

Fig. S7. *P. falciparum* microgametogenesis

Characterisation of *P. falciparum* (NF54) microgametogenesis *in vitro* which marks the immediate life cycle progression that occurs in the mosquito midgut following transmission from host to vector. **(A)** An IFA time-course of microgametogenesis, depicting individual channels and the merge of alpha tubulin- labelled cytoskeleton (green), glycophorin A-labelled host erythrocyte (red) and

Disease Models & Mechanisms • Supplementary information

DNA (blue). Drastic cytoskeletal rearrangement occurs across the entirety of the process as parasites egress from the host erythrocyte and replicate their genome three times, alternating with three endomitotic divisions. Scale bars = 10µm. **(B)** Electron microscopy of activated male gametocyte fixed at 25 minutes post activation. Distinctive cellular features of intraerythrocytic *P. falciparum* gametocytes, the Laveran's bib (LB) and Garnham bodies (G).

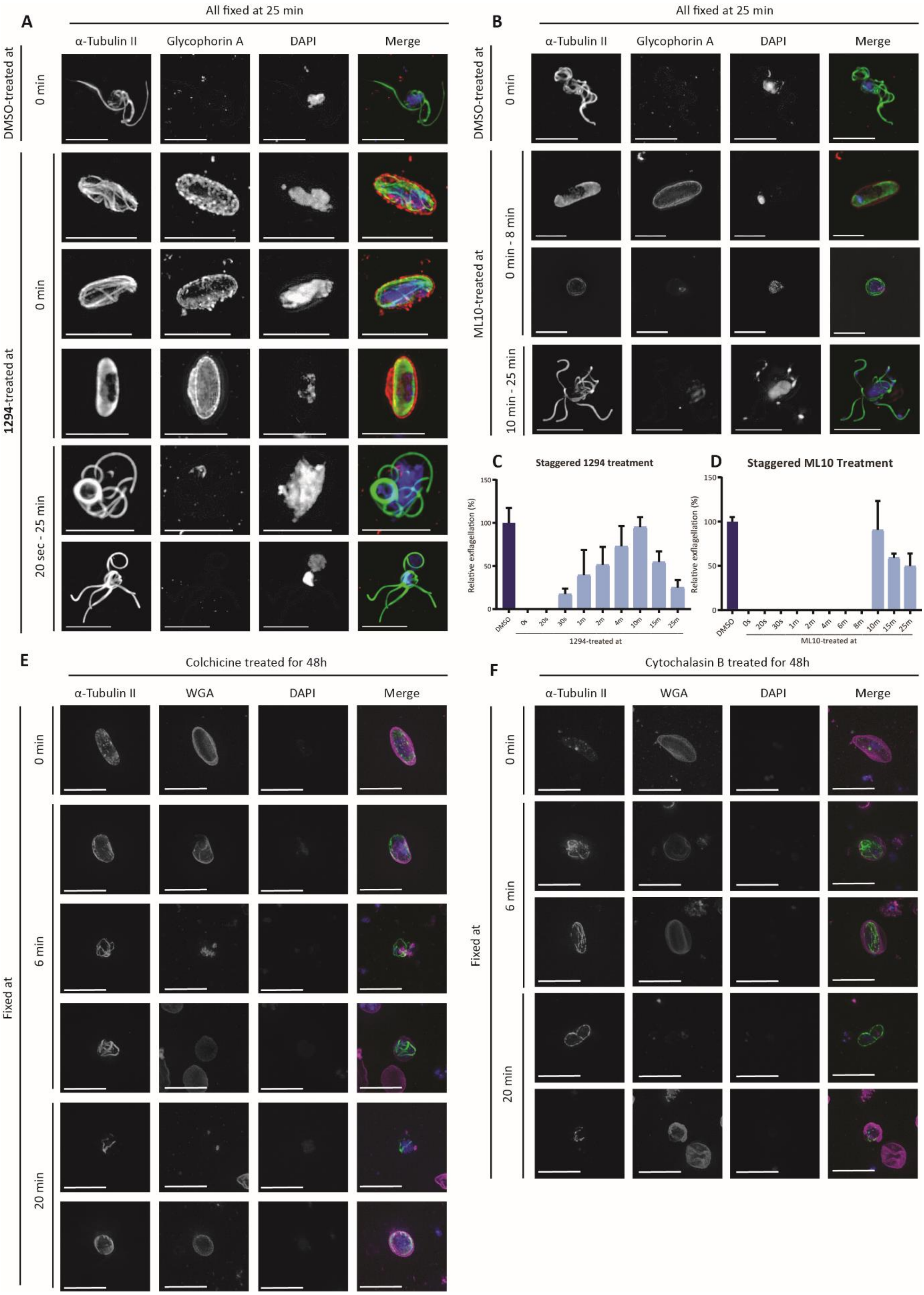
A
All fixed at 25 min
α-Tubulin II
Glycophorin A
DAPI
Merge
DMSO-treated at
0 min
1294-treated at
0 min
20 sec - 25 min
B
All fixed at 25 min
α-Tubulin II
Glycophorin A
DAPI
Merge
DMSO-treated at
0 min
ML10-treated at
0 min - 8 min
10 min - 25 min
C
Staggered 1294 treatment
Relative exflagellation (%)
150
100
50
0
DMSO
0s
20s
30s
1m
2m
4m
10m
15m
25m
1294-treated at
D
Staggered ML10 Treatment
Relative exflagellation (%)
150
100
50
0
DMSO
0s
20s
30s
1m
2m
4m
6m
8m
10m
15m
25m
ML10-treated at
E
Colchicine treated for 48h
α-Tubulin II
WGA
DAPI
Merge
Fixed at
0 min
6 min
20 min
F
Cytochalasin B treated for 48h
α-Tubulin II
WGA
DAPI
Merge
Fixed at
0 min
6 min
20 min

Fig. S8. Phenotype of known small molecular inhibitors of *P. falciparum* microgametogenesis

IFAs depicting phenotypes of **(A)** 1294, **(B)** ML10, **(D)** Colchicine and **(E)** Cytochalasin B during microgametogenesis. Individual channels of alpha tubulin-labelled cytoskeleton (green), glycophorin A-labelled (red) or WGA-labelled (far-red) host erythrocyte and DNA (blue) are shown alongside merged channels. Scale bars = 10µm. Parasites treated with **(A)** 1294 and **(B)** ML10 were activated, treated in time increments and fixed at 25 minutes, the point DMSO controls exflagellate. **(A)** 1294-treated gametocytes showed one of two phenotypes within the active window; i) gametocytes failed to develop at all, failing to replicate DNA, remaining falciform and intraerythrocytic, or ii) gametocytes failed to round up or egress, but host erythrocytes appeared fragmented, gametocytes replicated DNA and formed elongated axonemes which coiled around the falciform cell body. **(B)** ML10-treatment resulted in two distinct phenotypes; 1) gametocytes demonstrated no morphological progression and retained the morphology of intraerythrocytic stage V gametocytes which failed to fully replicate DNA or exflagellate or 2) gametocytes failed to exflagellate but rounded up and egressed from the host erythrocyte. Phenotypes are grouped according to the distinct phenotypes observed across 30 second-2-minute increments within the stated time windows. Exflagellation rates of gametocytes treated with **(C)** 1294 or **(D)** ML10 relative to DMSO controls when activated and treated at the stated time points. Error bars represent the SEM of 3 biological replicates. Gametocytes were treated with **(E)** Colchicine and **(F)** Cytochalasin B for 48 hours before activating and fixing at the stated timepoints relative to activation. **(E)** Colchicine treatment resulted in impaired axoneme formation and organisation but did not affect DNA replication. **(F)** Cells treated with Cytochalasin B exhibited impaired axoneme assembly and either rounded up without DNA replication or remained falciform but replicated DNA.

Disease Models & Mechanisms • Supplementary information

Table S1. Probe volumes added to lysate aliquots.

	Probe	Volume added (uL)	AzTB
A	DMSO control	10	+
B	Probe **2** 10 µM	10	-
C	Probe **2** 1 µM	1	+
D	Probe **2** 2 µM	2	+
E	Probe **2** 5 µM	5	+
F	Probe **2** 10 µM	10	+

Table S2. Fractionation elution buffer table.

SDB-RPS (1)	Ammonium Formate (100 mM), 40% ACN (v/v), 0.5% Formic Acid (v/v)
SDB-RPS (2)	Ammonium Formate (150 mM), 60% ACN (v/v), 0.5% Formic Acid (v/v)
Buffer	5% Ammonium Hydroxide (v/v), 80% ACN (v/v)

Table S3. PAL results

Click here to download Table S3

Table S4. Raw Counts

Click here to download Table S4

Disease Models & Mechanisms • Supplementary information

Supplementary Materials and Methods

CHEMICAL SYNTHESIS

GENERAL SYNTHESIS METHODS

All chemicals were purchased from Sigma-Aldrich Ltd, Fluorochem Ltd., Acros Organics and used without further purification. All reactions were performed under nitrogen or argon atmosphere using dried glassware. Silica gel column flash chromatography was performed using high-purity grade silica gel, pore size 60 Å, 220-440 mesh particle size, 35-75 µm particle size.

Analytical and preparatory HPLC runs were performed on a Agilent 1200 series system with a G1315D detector, a G1361A preparative pump, a semi-prep Daicel Chiralpak IE column 10×250 mm, 5 µm and eluting with 60:40 hexanes:EtOAc, 10 mL/min. For prep scale, 5–10 mg was injected at a time.

NMR spectra were recorded on 400 MHz Bruker instruments at room temperature and were referenced to residual solvent signals. Data are presented as follows: chemical shift, multiplicity (br s = broad singlet, s = singlet, d = doublet, t = triplet, q = quartet, m = multiplet) and integration.

The purity of compounds was verified by ^{1}H-NMR as well as RP-HPLC on a Waters 2767 system equipped with a photodiode array and an ES mass spectrometer. Separation was achieved using a XBridge C18 (5 µm, 4.6 mm × 100 mm) column, equipped with an XBridge C18 guard column (5 µm, 4.6 mm × 20 mm) eluting with a gradient of H_2O/ACN. Purity of tested compounds was ≥ 95%, unless specified.

Mass spectrometry was performed using chemical ionisation (CI), electron ionisation (EI) or electrospray ionisation on an AUTOSPEC P673 spectrometer by the Chemistry Department Mass Spectrometry Service at Imperial College London.

Unless otherwise stated all reactions were performed using anhydrous solvents, reacted under an atmosphere of argon, monitored by TLC and stirred at RT until completion.

SYNTHESIS OF PARENT MOLECULE 1

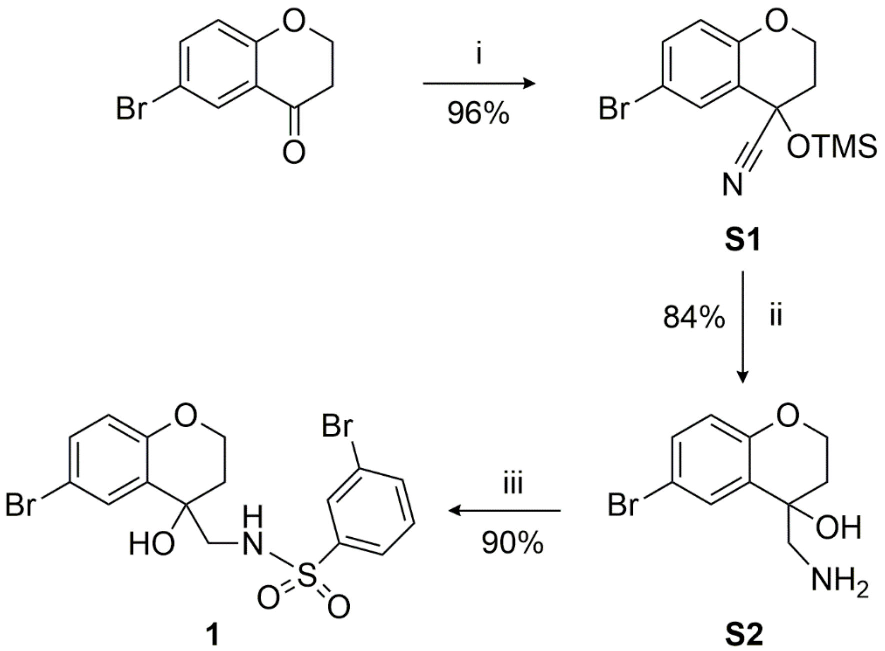

SCHEME 1: Synthetic route to affinity pulldown inhibitor, Compound 1. **i** - ZnI_2, TMS-CN, 50°C, ON. **ii** - $LiAlH_4$, THF, 0°C, 3 h. **iii** - 3-bromobenzenesulfonyl chloride, TEA, DCM, RT, 5 h.

Compound S1 - 6-bromo-4-((trimethylsilyl)oxy)chromane-4-carbonitrile

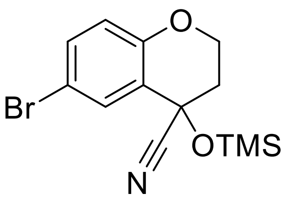

A solution of 6-bromochroman-4-one (454 mg, 2 mmol) and ZnI_2 (32 mg, 0.1 mmol) were suspended in DCM and cooled to 0 °C. Trimethylsilyl cyanide (0.38 mL, 3 mmol) was added dropwise and the resulting solution was stirred overnight. The resulting solution was diluted with DCM (20 mL), washed with $NaHCO_3$ (3 × 20 mL), extracted with DCM (2 × 60 mL), dried over $MgSO_4$ and concentrated *in vacuo* to afford the title compound, as an orange oil (626 mg, 96%). Due to column instability, compound **S1** was carried forward without further purification.

^{1}H NMR (400 MHz, Methanol-d$_4$) δ 7.63 (d, J = 2.5 Hz, 1H, ArC**H**), 7.45 (dd, J = 8.8, 2.5 Hz, 1H, ArC**H**), 6.83 (d, J = 8.9 Hz, 1H, ArC**H**), 4.43 – 4.31 (m, 2H, OC**H$_2$**CH$_2$), 2.49 (ddd, J = 14.0, 8.7, 4.4 Hz, 1H, OCH$_2$C**H$_2$**), 2.40 (ddd, J = 13.9, 5.5, 3.4 Hz, 1H, OCH$_2$C**H$_2$**), 0.23 (s, 9H, **TMS**). ^{13}C NMR (101 MHz, Methanol-d$_4$) δ 152.9, 133.9, 130.5, 123.1, 120.3, 119.5, 111.9, 65.3, 61.5, 35.5, -0.2. TLC (hexane: EtOAc – 3:1) Rf: 0.59

Compound S2 – 4-(aminomethyl)-6-bromochroman-4-ol

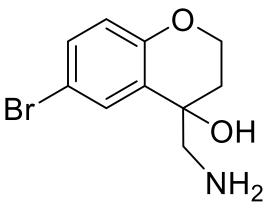

A suspension of $LiAlH_4$ (303 mg, 8 mmol) in THF was cooled to 0°C. Compound **S1** (652 mg, 2 mmol) was dissolved in THF and added drop-wise over 15 min. The reaction was stirred at 0°C until completion. The reaction was quenched using Fieser procedure, the following solutions were added

Disease Models & Mechanisms • Supplementary information

slowly at 0°C: dilute with Et_2O, H_2O (1 × w/v mass of $LiAlH_4$), 15% NaOH (1 × w/v mass of $LiAlH_4$), H_2O (1.5 × w/v mass of $LiAlH_4$). The solution was warmed to RT, stirred for 15 min, dried over $MgSO_4$, filtered and concentrated *in vacuo*. This afforded the tile compounds as a clear oil (433 mg, 84%).

^1H NMR (400 MHz, Methanol-d_4) δ 7.57 (d, J = 2.5 Hz, 1H, ArC**H**), 7.27 (dd, J = 8.8, 2.5 Hz, 1H, ArC**H**), 6.73 (d, J = 8.8 Hz, 1H, ArC**H**), 4.30 – 4.18 (m, 2H, OC**H_2**CH_2), 2.93 (q, 2H, COHC**H_2**NH_2), 2.23 (ddd, J = 14.1, 6.7, 3.6 Hz, 1H, OCH_2C**H_2**), 1.98 (ddd, J = 14.0, 8.1, 4.1 Hz, 1H, OCH_2C**H_2**), ^{13}C NMR (101 MHz, Methanol-d_4) δ 153.9, 131.4, 129.4, 129.3, 118.5, 112.0, 67.9, 63.2, 50.2, 31.7. MS: *m/z* (ES) 240 (50%, $[M-H]^+$), 258 (100%, $[M+H]^+$). HRMS, found 258.0134 ($C_{10}H_{13}NO_2Br$, $[M+H]^+$, requires 258.0130). TLC (DCM:MeOH – 90:10) R_f: 0.19.

Compound 1 – 3-bromo-N-((6-bromo-4-hydroxychroman-4-yl)methyl)benzenesulfonamide

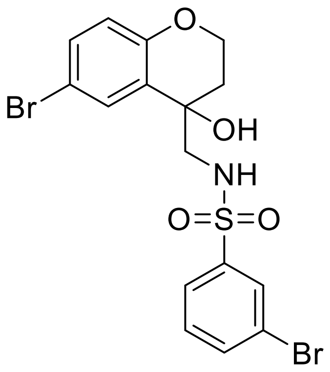

Compound **S2** (26 mg, 0.10 mmol) was dissolved in DCM under an inert atmosphere at 0 °C, before Triethylamine (TEA) (0.04 mL, 0.25 mmol) was added dropwise. A solution of 3-bromobenzenesulfonyl chloride (33 mg, 0.13 mmol) in DCM was then added over 10 min. The reaction was stirred at RT until completion. The resulting solution was diluted with H_2O (20 mL), extracted with DCM (3 × 20 mL), dried over $MgSO_4$ and concentrated *in vacuo*. The crude residue was purified by column chromatography (33% Pet. Ether in Et_2O) to afford the title compound, as a white foam (43 mg, 90%).

^1H NMR (400 MHz, Methanol-d_4) δ 8.01 (t, *J* = 1.9, 1.9 Hz, 1H, ArC**H**), 7.82 (dt, *J* = 7.9, 1.3, 1.3 Hz, 1H, ArC**H**), 7.78 (dt, 1H, ArC**H**), 7.45 – 7.51 (m, 2H, ArC**H**), 7.25 (dd, *J* = 8.8, 2.5 Hz, 1H, ArC**H**), 6.70 (d, *J* = 8.7 Hz, 1H, ArC**H**), 4.28 – 4.19 (m, 2H, OC**H_2**CH_2), 3.31 – 3.10 (m, 2H, COHC**H_2**NH_2), 2.38 – 2.30 (m, 1H, OCH_2C**H_2**), 2.01 – 1.92 (m, 1H, OCH_2C**H_2**). ^{13}C NMR (101 MHz, Methanol-d_4) δ 153.8, 142.8, 135.2, 131.7, 130.7, 129.7, 129.2, 128.2, 125.2, 122.5, 118.5, 111.9, 67.3, 63.2, 51.1, 32.0. MS: *m/z* (ES) 474 $[M-H]^-$. HRMS, found 473.9025 ($C_{16}H_{14}NO_4SBr_2$, $[M-H]^-$, requires 473.9010). TLC (hexane:EtOAc - 1:1) R_f: 0.43.

Disease Models & Mechanisms • Supplementary information

SYNTHESIS OF ARYL AZIDE PROBE 2

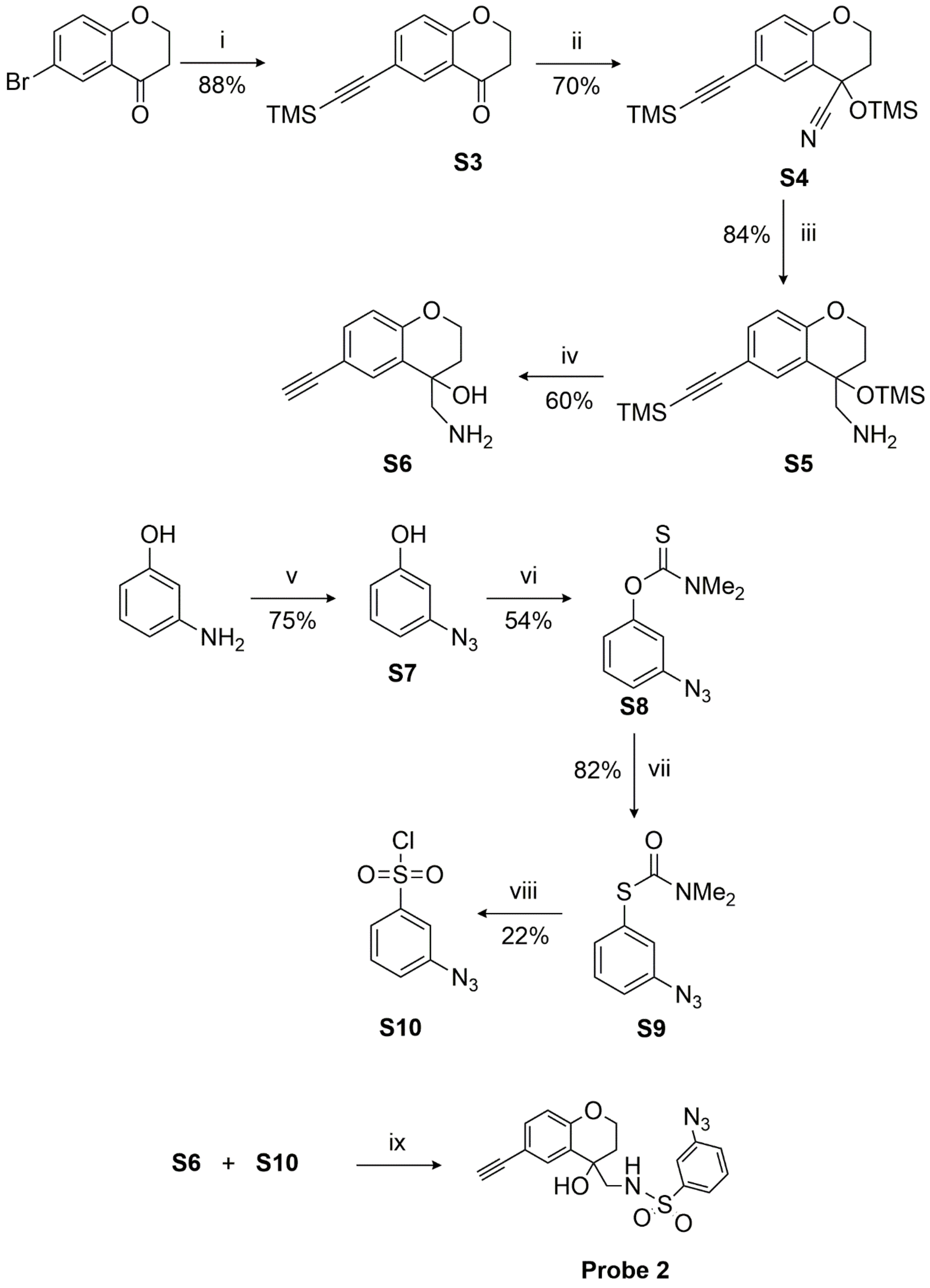

Disease Models & Mechanisms • Supplementary information

SCHEME 2: Synthetic route to aryl azide containing probe 2. **i** - CuI, $PdCl_2(PPh_3)_2$, TEA, $C_5H_{10}Si$, DCM, 70°C, 24 h. **ii** - ZnI_2, TMS-CN, 50°C, ON. **iii** - $LiAlH_4$, THF, 0°C, 3 h. **iv** - K_2CO_3, DCM:MeOH (5:2), RT, 5 h. **v** - HCl/H_2O, $NaNO_2$, 0°C, 10 min. Followed by NaN_3, 0°C, 1 h. **vi** - DABCO, NMP, 50°C, 24 h. **vii** - $Pd(t\text{-}Bu_3P)_2$, Toluene, 100°C, 72 h. **viii** - NCS, HCl, ACN, 0°C, 6 h. **ix** – TEA, DCM, RT, 5 h.

Compound S3 - 6-((trimethylsilyl)ethynyl)chroman-4-one

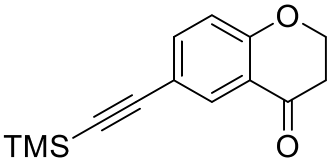

A solution of 6-bromo-4-chromanone (1.2 g, 5.3 mmol), CuI (30 mg, 0.2 mmol) and TEA (18 mL, 15.9 mmol) was sparged with argon. Bis(triphenyl phosphine) palladium chloride (112 mg, 0.2 mmol) and trimethylsilylacetylene (2.2 mL, 15.9 mmol) were added, the mixture was heated to 75 °C and stirred for 24 h. The resulting solution was diluted into Et_2O (20 mL), washed with NH_4Cl:brine 9:1 (3 × 20 mL), washed with brine (60 mL), dried over $MgSO_4$ and concentrated *in vacuo*. The crude residue was purified by column chromatography (0 to 50 % EtOAc in Hexane) to afford the title compound, as a pale yellow oil (1.1 g, 88%).

^1H NMR (400 MHz, $CDCl_3$) δ 8.01 (d, J = 2.0 Hz, 1H, ArC**H**), 7.53 (dd, J = 8.6, 2.2 Hz, 1H, ArC**H**), 6.90 (d, J = 8.6 Hz, 1H, ArC**H**), 4.58 – 4.50 (m, 2H, $OC\mathbf{H_2}CH_2$), 2.85 – 2.77 (m, 2H, $OCH_2C\mathbf{H_2}$), 0.23 (s, 9H, TMS).^{13}C NMR (101 MHz, $CDCl_3$) δ 191.0, 161.7, 139.0, 131.2, 121.1, 118.2, 116.7, 103.8, 94.0, 67.2, 37.7, 0.1.

Compound S4 - 6-((trimethylsilyl)ethynyl)-4-((trimethylsilyl)oxy)chromane-4-carbonitrile

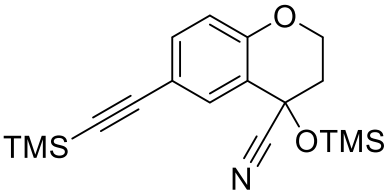

Compound **S3** (730 mg, 3.0 mmol) and ZnI_2 (19 mg, 0.06 mmol) were suspended in DCM and cooled to 0 °C. Trimethylsilyl cyanide (560 µL, 4.5 mmol) was added dropwise and the resulting solution was stirred overnight. The resulting solution was diluted with DCM (20 mL), washed with $NaHCO_3$ (3 × 20 mL), extracted with DCM (2 × 60 mL), dried over $MgSO_4$ and concentrated *in vacuo* to afford the title compound, as an orange oil (718 mg, 70%). Due to column instability, compound **S4** was carried forward without further purification.

^1H NMR (400 MHz, Methanol-d_4) δ 7.58 (d, J = 2.0 Hz, 1H, ArC**H**, 7.37 (dd, J = 8.6, 2.1 Hz, 1H, ArC**H**), 6.83 (d, J = 8.6 Hz, 1H, ArC**H**), 4.43 – 4.34 (m, 2H, $OC\mathbf{H_2}CH_2$), 2.53 – 2.35 (m, 2H, $OCH_2C\mathbf{H_2}$), 0.26 (s, 9H, TMS), 0.21 (s, 9H, TMS).

Compound S5 - (6-((trimethylsilyl)ethynyl)-4-((trimethylsilyl)oxy)chroman-4-yl)methanamine

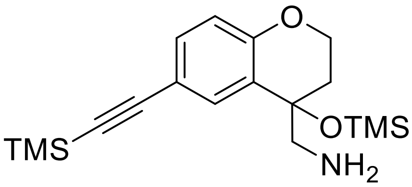

Compound **S4** (718 mg, 2.1 mmol) was dissolved in THF and added dropwise, over 15 min, to a stirred suspension of $LiAlH_4$ (319 mg, 8.4 mmol) in THF pre-cooled to 0°C. The reaction was

Disease Models & Mechanisms • Supplementary information

stirred at 0°C until completion. The reaction was quenched using the Fieser procedure, the following solutions were added slowly at 0 °C: dilute with Et_2O, H_2O (1 × w/v mass of $LiAlH_4$), 15% NaOH (1 × w/v mass of $LiAlH_4$), H_2O (1.5 × w/v mass of $LiAlH_4$). The solution was warmed to RT, stirred for 15 min, dried over $MgSO_4$, filtered and concentrated *in vacuo* to afford the title compound, as an orange oil (425 mg, 84%).

^{1}H NMR (400 MHz, Methanol-d_4) δ 7.46 (d, J = 2.1 Hz, 1H, ArC**H**), 7.24 (dd, J = 8.5, 2.1 Hz, 1H, ArC**H**), 6.74 (d, J = 8.5 Hz, 1H, ArC**H**), 4.35 – 4.26 (m, 1H, COHC**H₂**NH₂), 4.24 – 4.17 (m, 1H, COHC**H₂**NH₂), 3.00 – 2.86 (m, 2H, OC**H₂**CH₂), 2.30 – 2.17 (m, 1H, OCH₂C**H₂**), 2.14 – 2.03 (m, 1H, OCH₂C**H₂**), 0.22 (s, 9H, TMS), -0.02 (s, 9H, TMS).

Compound S6 - 4-(aminomethyl)-6-ethynylchroman-4-ol

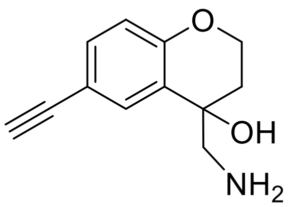

Compound **S5** was shown by ^{1}H NMR to be a mixture of trimethylsilyl (TMS) protected/ deprotected compound, therefore was fully deprotected before further purification. Compound **S5** (425 mg, 1.2 mmol) was dissolved in DCM in MeOH (5:2, 5 mL), before K_2CO_3 (200 mg, 1.5 mmol) was added and solution stirred at RT for 5 h. The resulting solution was diluted with H_2O (20 mL), extracted with DCM (3 × 20 mL), washed with $NaHCO_3$ (2 × 60 mL), dried over $MgSO_4$ and concentrated *in vacuo*. The crude residue was purified by column chromatography (2% to 5% 7M NH_3 MeOH in DCM) to afford the title compound, as an orange oil (150 mg, 60%).

^{1}H NMR (400 MHz, Methanol-d_4) δ 7.56 (d, J = 2.1 Hz, 1H, ArC**H**), 7.24 (dd, J = 8.5, 2.1 Hz, 1H, ArC**H**), 6.74 (d, J = 8.5 Hz, 1H, ArC**H**), 4.29 – 4.18 (m, 2H, OC**H₂**CH₂), 2.97 – 2.82 (m, 2H, COHC**H₂**NH₂), 2.24 – 2.15 (m, 1H, OCH₂C**H₂**), 2.00 – 1.91 (m, 1H, OCH₂C**H₂**). ^{13}C NMR (101 MHz, Methanol-d_4) δ 156.5, 133.6, 132.0, 128.5, 118.1, 115.5, 84.5, 76.9, 69.1, 64.6, 51.5, 33.2. MS: *m/z* (ES) 186 (100%, $[M-OH]^+$), 204 (85%, $[M+H]^+$). HRMS, found 204.1025 ($C_{12}H_{14}NO_2$, $[M+H]^+$, requires 204.1025). TLC (DCM:MeOH – 90:10) R_f: 0.14.

Compound S7 - 3-azidophenol

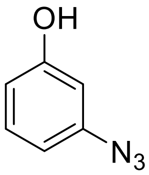

3-Aminophenol (400 mg, 3.6 mmol) was dissolved in H_2O (1.6 mL) and HCl (1.6 mL) and cooled to 0 °C. $NaNO_2$ was dissolved in H_2O (10 mL), cooled to 0 °C and added dropwise to the reaction over 10 min. The reaction was stirred 20 min at 0°C, before NaN_3 (240 mg, 3.6 mmol) in H_2O (4 mL) was added dropwise over 10 min. The reaction was stirred for 90 min at 0 °C, before dilution in H_2O (20 mL). The aqueous phase was extracted with Et_2O (3 × 30 mL) and the combined organic phases were washed with $NaHCO_3$ (3 × 100 mL), brine (2 × 100 mL), dried over $MgSO_4$ and concentrated *in vacuo*. The crude residue was purified by column chromatography (10% EtOAc in Hexane) to afford the title compound, as a colourless oil (400 mg, 75%).

^{1}H NMR (400 MHz, $CDCl_3$) δ 7.21 (t, J = 8.1 Hz, 1H, ArC**H**), 6.68 – 6.59 (m, 2H, ArC**H**), 6.52 (t, J = 2.2 Hz, 1H, ArC**H**), 5.87 (s, 1H, ArCO**H**). ^{13}C NMR (101 MHz, $CDCl_3$) δ 156.7, 141.5, 130.9, 112.3, 111.7, 106.4. Analysed by analytical LC-MS 20-98% ACN in H_2O R_t = 8.65 min, ES^- $(M - H)^-$ m/z = 134.

Compound S8 - O-(3-azidophenyl) dimethylcarbamothioate

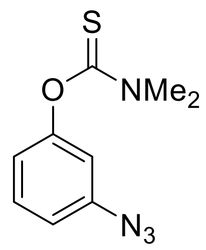

Compound **S7** (400 mg, 2.9 mmol) and 1,4-diazabicyclo[2.2.2]octane (395 mg, 3.2 mmol) were dissolved in NMP (15 mL), before dimethyl thiocarbamoyl chloride (406 mg, 3.6. mmol) was added portion-wise over 10 min. The reaction was stirred at 50°C for 48 h and the resulting solution was diluted EtOAc (50 mL), washed with H_2O (3 × 50 mL), brine (2 × 50 mL), dried over $MgSO_4$ and concentrated *in vacuo*. The crude residue was purified by column chromatography (8 to 15% EtOAc in Hexane) to afford the title compound, as a colourless oil (354 mg, 54%).

^{1}H NMR (400 MHz, $CDCl_3$) δ 7.34 (t, J = 8.1 Hz, 1H, ArC**H**), 6.91 (ddd, J = 8.1, 2.2, 0.9 Hz, 1H, ArC**H**), 6.86 (ddd, J = 8.2, 2.2, 0.9 Hz, 1H, ArC**H**), 6.76 (t, J = 2.2 Hz, 1H, ArC**H**), 3.43 (s, 3H, N(C**H**$_3$)$_2$), 3.32 (s, 3H, N(C**H**$_3$)$_2$). ^{13}C NMR (101 MHz, $CDCl_3$) δ 187.2, 154.9, 140.9, 130.0, 119.5, 116.5, 113.9, 43.3, 38.7.

Compound S9 - S-(3-azidophenyl) dimethylcarbamothioate

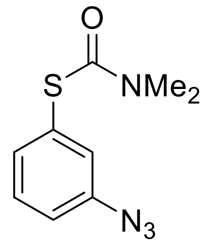

Compound **S8** (100 mg, 0.45 mmol) was dissolved in toluene (5 mL) and heated to 100°C under argon. Pd(tBu$_3$P)$_2$ (5.0 mg, 2 mol%) was added and the reaction stirred 72 h at 100°C. The resulting solution was concentrated *in vacuo* and the crude residue was purified by column chromatography (5 to 20 % EtOAc in Hexane) to afford the title compound, as a colourless oil (82 mg, 82%). The reaction was monitored by IR, for the appearance of an indicative carbonyl stretch at 1667.13 cm^{-1}.

^{1}H NMR (400 MHz, $CDCl_3$) δ 7.38 (t, J = 7.8 Hz, 1H, ArC**H**), 7.32 – 7.28 (m, 1H, ArC**H**), 7.22 (t, J = 2.0 Hz, 1H, ArC**H**), 7.09 – 7.04 (m, 1H, ArC**H**), 3.15 – 3.01 (m, 6H, N(C**H**$_3$)$_2$). ^{13}C NMR (101 MHz, $CDCl_3$) δ 166.2, 140.6, 132.2, 130.1, 126.1, 120.0, 37.0. IR v_{max} (neat, cm^{-1}) 2900 (N_3), 1667 (CO). HRMS, found 223.0650 ($C_9H_{114}OS$, $[M + H]^+$, requires 223.0654).

A small amount of starting material was observed in the ^{1}H and ^{13}C spectra, the crude mixture was carried through to next reaction.

Compound S10 - 3-azidobenzenesulfonyl chloride

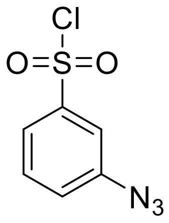

Compound **S9** (85 mg, 0.38 mmol) was suspended in ACN (2.6 mL)/HCl (2M, 530 *m*L) and cooled to 0 °C. N-chlorosuccinimide (200 mg, 1.5 mmol) was dissolved in

Disease Models & Mechanisms • Supplementary information

ACN (4 mL) and added dropwise to the reaction. The resulting solution was stirred at 0°C for 6 h and diluted with iPr_2O (20 mL). The organic phase was washed with H_2O (3 × 30 mL) and brine (2 × 30 mL), dried over $MgSO_4$ and concentrated *in vacuo*. The crude residue was purified by column chromatography (5 to 30% EtOAc in Hexane) to afford the title compound, as a colourless oil (18 mg, 22%).

^{1}H NMR (400 MHz, $CDCl_3$) δ 7.83 – 7.78 (m, 1H, ArC**H**), 7.67 (t, J = 2.1 Hz, 1H, ArC**H**), 7.62 (t, J = 8.0 Hz, 1H, ArC**H**), 7.40 – 7.35 (m, 1H, ArC**H**). ^{13}C NMR (101 MHz, $CDCl_3$) δ 145.8, 142.4, 131.3, 125.6, 123.1, 117.4.

Compound 2 - 3-azido-N-((6-ethynyl-4-hydroxychroman-4-yl)methyl)benzenesulfonamide

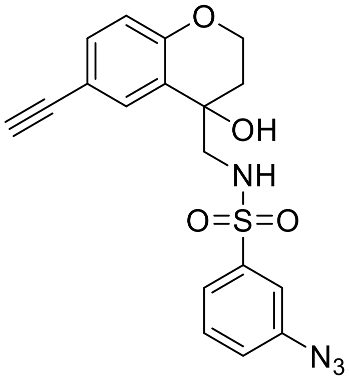

Compound **S6** (22 mg, 0.078 mmol) was dissolved in DCM under an inert atmosphere at 0°C, before triethylamine (TEA) (27 µL, 0.195 mmol) was added dropwise. A solution of compound **S10** (20 mg, 0.094 mmol) in DCM was then added over 10 min. The reaction was stirred at RT until completion. The resulting solution was diluted with H_2O (20 mL), extracted with DCM (3 × 20 mL), dried over $MgSO_4$ and concentrated *in vacuo*. The crude residue was purified by column chromatography (25% EtOAc in Hexane) to afford the title compound, as a white foam (15 mg, 56%). A 10 mM DMSO stock was prepared for biological testing.

^{1}H NMR (400 MHz, DMSO-d_6) δ 7.87 (s, 1H, CO**H**CH_2NH), 7.62 – 7.57 (m, 2H, ArC**H**), 7.50 (s, 1H, ArC**H**), 7.44 (d, J = 2.1 Hz, 1H, ArC**H**), 7.39 – 7.32 (m, 1H, ArC**H**), 7.23 (dd, J = 8.5, 2.0 Hz, 1H, ArC**H**), 6.74 (d, J = 8.4 Hz, 1H, ArC**H**), 5.46 (s, 1H, SO_2N**H**), 4.31 – 4.09 (m, 2H, OC$\mathbf{H_2}$$CH_2$), 3.98 (s, 1H, C≡C**H**), 3.12 (d, J = 13.3 Hz, 1H, COHC$\mathbf{H_2}$NH), 2.94 (d, J = 13.2 Hz, 1H, COHC$\mathbf{H_2}$NH), 2.29 – 2.16 (m, 1H, OCH_2C$\mathbf{H_2}$), 1.92 – 1.78 (m, 1H, OCH_2C$\mathbf{H_2}$). ^{13}C NMR (101 MHz, DMSO-d_6) δ 154.7, 142.4, 140.6, 132.2, 131.4, 130.8, 127.2, 123.0, 122.9, 116.9, 116.8, 113.2, 83.7, 78.9, 66.6, 63.1, 50.8, 31.9. HRMS, found 383.0820 ($C_{18}H_{15}N_4O_4S$, $[M - H]^-$, requires 383.0814).

SYNTHESIS OF COMPOUND 3

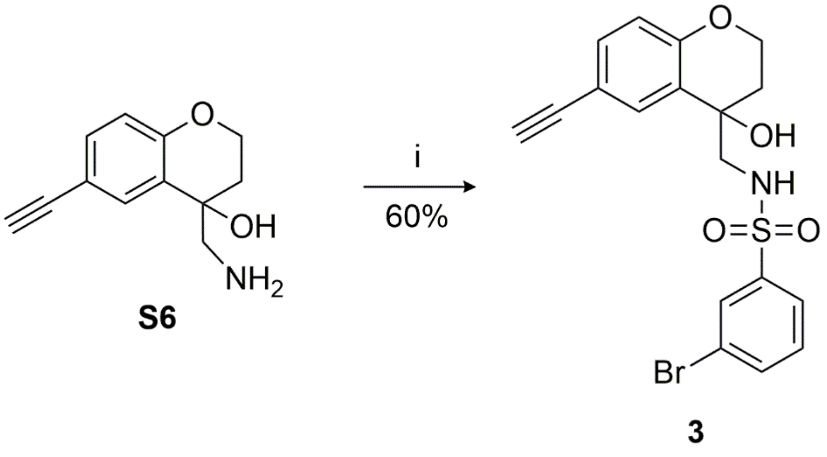

SCHEME 3: Synthesis of 3. i TEA, DCM, RT, 5 h.

Compound 3 - 3-Bromo-N-((6-ethynyl-4-hydroxychroman-4-yl)methyl)benzenesulfonamide

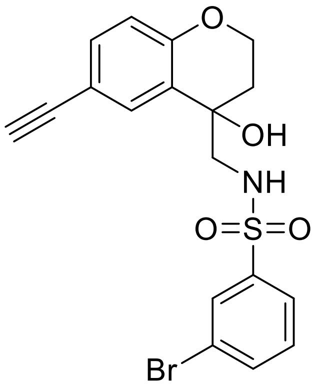

Compound **S6** (86 mg, 0.4 mmol) was dissolved in DCM under an inert atmosphere at 0 °C before TEA (27 µL, 1 mmol) was added dropwise. A solution of 3-bromobenzenesulfonyl chloride (149 mg, 0.6 mmol) in DCM was then added over 10 min. The reaction was stirred at RT until completion. The resulting solution was diluted with H_2O (20 mL), extracted with DCM (3 × 20 mL), dried over $MgSO_4$ and concentrated in vacuo. The crude residue was purified by column chromatography (33% Pet. Ether in Et_2O) to afford the title compound, as a white foam (150 mg, 60%).

^{1}H NMR (400 MHz, Methanol-d_4) δ 8.00 (t, J = 1.8 Hz, 1H, ArC**H**), 7.82 – 7.79 (m, 1H, ArC**H**), 7.78 – 7.74 (m, 1H, ArC**H**), 7.50 – 7.44 (m, 2H, ArC**H**), 7.22 (dd, J = 8.5, 2.1 Hz, 1H, ArC**H**), 6.72 (d, J = 8.5 Hz, 1H, ArC**H**), 4.26 –4.22 (m, 2H, OC$\mathbf{H_2}$CH_2), 3.31 (s, 1H, C≡C**H**), 3.27 (d, J = 13.4 Hz, 1H, COHC$\mathbf{H_2}$NH), 3.12 (d, J = 13.4 Hz, 1H, COHC$\mathbf{H_2}$NH), 2.37 – 2.29 (m, 1H, OCH_2C$\mathbf{H_2}$), 2.01 – 1.92 (m, 1H, OCH_2C$\mathbf{H_2}$); LCMS - 18 min 50 to 98% ACN in H_2O showed 92% purity. ES−m/z $(M - H)^-$ = 423.

Disease Models & Mechanisms • Supplementary information

SYNTHESIS OF COMPOUND 4

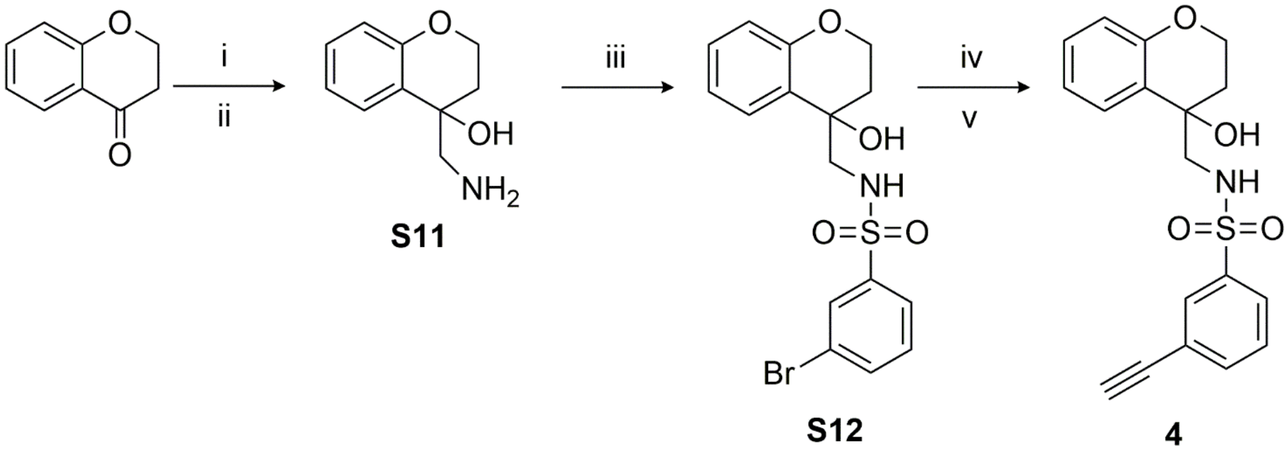

SCHEME 4: Synthesis of 4. i - TMSCN, ZnI2, DCM, rt. **ii** - $LiAlH_4$, THF, 70°C. **iii** - 3-bromobenzenesulfonyl chloride, TEA, DCM, rt. **iv** - Ethynyltrimethylsilane, $Pd(Ph_3)_2Cl_2$, CuI, TEA, DMF, 100°C. **v** - TBAF, THF, rt.

Compound S11 – 4-(aminomethyl)chroman-4-ol

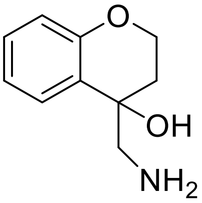

4-Chromanone (1.48 g, 10 mmol, 1 eq.) and zinc iodide (64 mg, 0.20 mmol, 0.02 eq.) was taken up in dry DCM (30 mL) and cooled to 0 °C under N_2. Cyanotrimethylsilane (1.8 mL, 15 mmol, 1.5 eq.) was added dropwise. The reaction mixture was allowed to warm up to room temperature and stirred overnight. It was then diluted with DCM (50 mL) and washed with a solution of sat. $NaHCO_3$ (3×). The combined organic phases were dried over $MgSO_4$ and concentrated *in vacuo*. The resulting orange oil was then used without further purification (2.42 g, 9.8 mmol, 98% yield >95% purity).

^{1}H NMR (400 MHz, $CDCl_3$) δ 7.59 (dd, *J* = 7.9, 1.7 Hz, 1H, ArC**H**), 7.32 (td, 1H, ArC**H**), 7.01 (td, *J* = 7.7, 7.6, 1.2 Hz, 1H, ArC**H**), 6.87 (dd, *J* = 8.3, 1.2 Hz, 1H, ArC**H**), 4.44 – 4.32 (m, 2H, OC**H_2**CH_2), 2.51 – 2.36 (m, 2H, OCH_2C**H_2**), 0.20 (s, 9H, **TMS**). ^{13}C NMR (101 MHz, $CDCl_3$) δ 153.5, 131.4, 128.7, 121.0, 120.8, 120.7, 117.6, 65.6, 61.3, 36.3, 1.2. TLC (hexane: EtOAc – 3:2) Rf: 0.86.

$LiAlH_4$ (835 mg, 2.2 mmol, 2.1 eq.) was dissolved in THF (20 mL) under N_2 at 0 °C. 4-((trimethylsilyl)oxy)chromane-4-carbonitrile (2.42 g, 9.8 mmol, 1 eq.) dissolved in THF (40 mL) was added dropwise. The mixture was then heated up at reflux for 2 h. It was then cooled down to 0 °C and worked-up according to the *Fieser*-procedure. The crude product was obtained as a yellow oil which was purified by flash column chromatography (DCM → DCM: 7M NH_3 in MeOH – 9:1) and obtained as a yellow, viscous liquid (1.423g, 7.9 mmol, 79%).

Disease Models & Mechanisms • Supplementary information

^{1}H NMR (400 MHz, Methanol-d_4) δ 7.44 (dd, 1H, ArC**H**). 7.16 (dt, 1H, ArC**H**), 6.93 (dt, 1H, ArC**H**), 6.79 (dd, 1H, ArC**H**), 4.29 – 4.18 (m, 2H, OC**H₂**CH₂), 2.96 (q, 2H, COHC**H₂**NH₂), 2.24 (ddd, 1H, OCH₂C**H₂**), 1.98 (ddd, 1H, OCH₂C**H₂**), ^{13}C NMR (101 MHz, DMSO-d_6) δ 154.7, 129.0, 128.5, 127.7, 120.4, 116.5, 68.2, 63.6, 51.6, 32.4. MS: *m/z* (ES) 180 (50%, $[M+H]^+$), 162 (100%, [M-OH]+). HRMS, found 180.1024 ($C_{10}H_{13}NO_2$, $[M+H]^+$, requires 180.1025). TLC (DCM:MeOH - 90:10) Rf: 0.13.

Compound S12 - 3-bromo-N-((4-hydroxychroman-4-yl)methyl)benzenesulfonamide

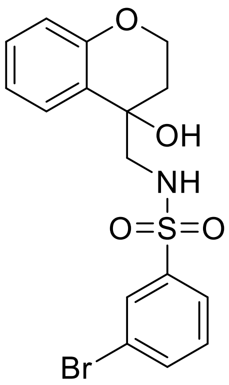

Compound **S11** (450 mg, 2.5 mmol) was dissolved in DCM under an inert atmosphere at 0 °C before TEA (170 µL, 6.25 mmol) was added dropwise. A solution of 3-bromobenzenesulfonyl chloride (900 mg, 3.5 mmol) in DCM was then added over 10 min. The reaction was stirred at RT until completion. The resulting solution was diluted DCM (20 mL), neutralised, washed with H_2O (3 × 30 mL), extracted with DCM (2 × 90 mL), washed with brine (200 mL), dried over $MgSO_4$ and concentrated in vacuo. The crude residue was purified by column chromatography (33% EtOAc in Hexane) to afford the title compound, as a white foam (742 mg, 75%).

^{1}H NMR (400 MHz, $CDCl_3$) δ 8.02 (t, J = 1.8 Hz, 1H, ArC**H**), 7.82 – 7.78 (m, 1H, ArC**H**), 7.76 – 7.73 (m, 1H, ArC**H**), 7.43 (t, J = 7.9 Hz, 1H, ArC**H**), 7.33 (dd, J = 7.8, 1.7 Hz, 1H, ArC**H**), 7.28 – 7.19 (m, 1H, ArC**H**), 6.93 (td, J = 7.5, 1.2 Hz, 1H, ArC**H**), 6.87 (dd, J = 8.3, 1.2 Hz, 1H, ArC**H**), 5.02 – 4.94 (m, 1H, SO₂N**H**), 4.32 – 4.21 (m, 2H, OC**H₂**CH₂), 3.41 – 3.24 (m, 2H, COHC**H₂**NH), 2.49 – 2.38 (m, 1H, OCH₂C**H₂**), 2.10 – 2.02 (m, 1H, OCH₂C**H₂**); Analysed by Analytical LC-MS 20-98% ACN in H_2O Rt = 10.8 min, ES− $(M - H)^-$ m/z = 398.

Compound 4 – 3-ethynyl-*N*-((4-hydroxychroman-4-yl)methyl)benzenesulfonamide

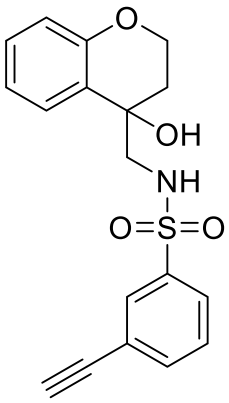

A mixture of **S12** (17 mg, 0.05 mmol, 1 eq.), CuI (5 mol%, 0.5 mg), dichlorobis(triphenylphosphine)palladium(II) (5 mol%, 1.5 mg), trimethylsilylacetylene (0.04 mL, 0.25 mmol, 5 eq.), and triethylamine (0.5 mL) in DMF (2 mL) in a microwave vial was stirred at 100°C for 1 h under microwave irradiation under argon. The mixture was diluted with toluene, filtered over celite and concentrated *in vacuo*. It was then dissolved in dry THF (1 mL) under N_2. Tetrabutylammonium fluoride (1 M in THF, 0.15 mL, 0.15 mmol, 3 eq.) was added dropwise. The reaction mixture was stirred at room temperature for 2.5 h. The reaction mixture was then diluted with EtOAc (10 mL) and washed with a solution of sat. NH_4Cl (aq.) (3×) and brine. The organic phases were combined, dried and concentrated *in vacuo* to give the crude product as a brown solid. The product was purified via flash

Disease Models & Mechanisms • Supplementary information

column chromatography on deactivated silica (hexane:EtOAc – 4:1). The resulting solid was further purified by preparative HPLC (H_2O:ACN 50:50 → ACN) to give the product as a white solid (1.7 mg, 0.005 mmol, 12% yield over two steps, ~90 % purity).

^{1}H NMR (400 MHz, $CDCl_3$) δ 7.99 (s, 1H, ArC**H**), 7.84 (dt, *J* = 7.9, 1.5, 1.5 Hz, 1H, ArC**H**), 7.71 (dt, *J* = 7.8, 1.3, 1.3 Hz, 1H, ArC**H**), 7.52 (t, *J* = 7.8, 7.8 Hz, 1H, ArC**H**), 7.33 (dd, *J* = 7.8, 1.6 Hz, 1H, ArC**H**), 7.24 (td, *J* = 8.7, 7.2, 1.7 Hz, 1H, ArC**H**), 6.93 (td, *J* = 7.6, 7.5, 1.2 Hz, 1H, ArC**H**), 6.87 (dd, *J* = 8.3, 1.2 Hz, 1H, ArC**H**), 4.90 (q, 1H, $SO_2N\mathbf{H}$), 4.34 – 4.20 (m, 2H, $OC\mathbf{H_2}CH_2$), 3.39 – 3.26 (m, 2H, $COHC\mathbf{H_2}NH$), 3.22 (s, 1H, C≡C**H**), 2.44 (ddd, *J* = 14.2, 6.3, 3.5 Hz, 1H, $OCH_2C\mathbf{H_2}$), 2.05 (ddd, *J* = 13.3, 8.2, 4.2 Hz, 1H, $OCH_2C\mathbf{H_2}$). *^{13}C NMR data not available due to low quantity.* MS: *m/z* (ES) 342 [M-H]-. HRMS, found 342.0762 ($C_{18}H_{16}NO_4S$, [M-H]-, requires 342.0800). TLC (hexane:EtOAc - 1:1) Rf: 0.46.

Disease Models & Mechanisms • Supplementary information

SYNTHESIS OF COMPOUND 5 AND ETHER LINKED ALKYNE PROBES 8 AND 9

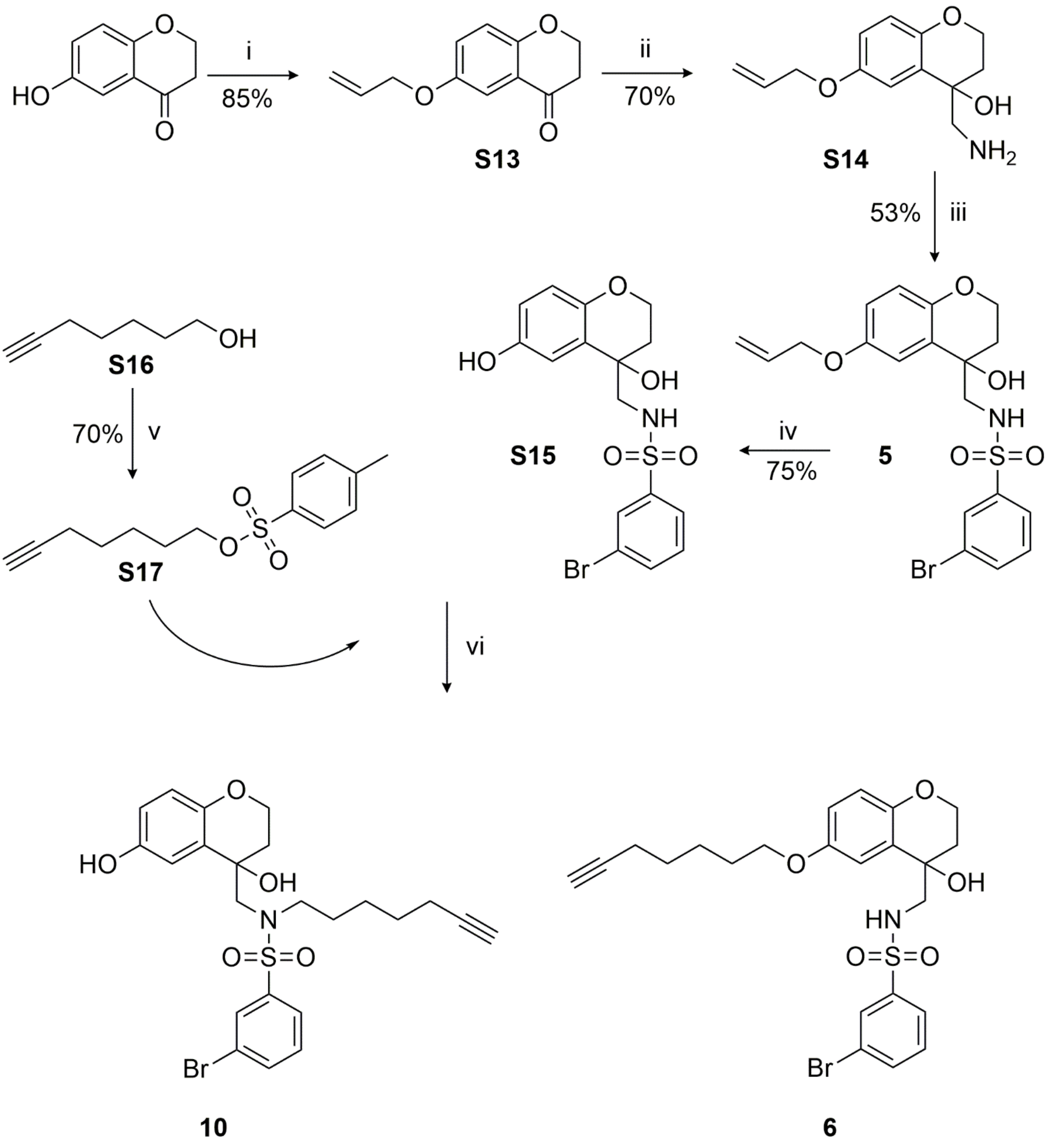

SCHEME 5: Synthesis of ether linked alkyne probe - compounds 8 and 9. i - K_2CO_3, Acetone, 70°C, 24 h. **ii** - ZnI_2, TMS-CN, 50°C, ON. Followed by $LiAlH_4$, THF, 0°C, 3 h. **iii** - TEA, DCM, RT, 5 h. **iv** - $Pd(PPh_3)_4$, K_2CO_3, MeOH, 60°C, ON. **v** - DMAP, TEA, DCM, 0°C, 30 min. **vi** - K_2CO_3, DMF, RT, 72 h.

Disease Models & Mechanisms • Supplementary information

Compound S13 - 6-(allyloxy)chroman-4-one

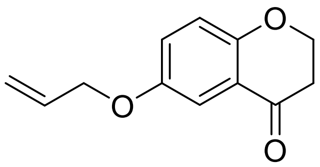

A suspension of 6-hydroxy-4-chromanone (830 mg, 5.1 mmol) and K_2CO_3 (1.1 g, 7.6 mmol) was prepared in acetone (30 mL). Allyl bromide (480 *μ*L, 5.1 mmol) was added dropwise and the reaction was allowed to reflux, at 56°C, for 24 h. The resulting solution was washed with sat. NH_4Cl (3 × 30 mL) and extracted with EtOAc (2 × 80 mL). The combined organic phases washed with brine (100 mL), dried over $MgSO_4$ and concentrated in vacuo. The crude residue was purified by column chromatography (10% EtOAc in Hexane) to afford the title compound, as a colourless oil (880 mg, 85%).

^{1}H NMR (400 MHz, $CDCl_3$) δ 7.23 (d, J = 3.2 Hz, 1H, ArC**H**), 7.03 (dd, J = 9.0, 3.2 Hz, 1H, ArC**H**), 6.82 (d, J = 9.0 Hz, 1H, ArC**H**), 6.04 – 5.89 (m, 1H, $OCH_2C\mathbf{H}CH_2$), 5.35 (*app-dq, J = 17.3, 1.6 Hz, 1H, $OCH_2CHC\mathbf{H_2}$), 5.22 (*app-dq, J = 10.5, 1.3 Hz, 1H, $OCH_2CHC\mathbf{H_2}$), 4.45 – 4.39 (m, 4H, $OC\mathbf{H_2}CHCH_2$ and $OC\mathbf{H_2}CH_2$), 2.75 – 2.67 (m, 2H, $OCH_2C\mathbf{H_2}$); ^{13}C NMR (101 MHz, $CDCl_3$) δ 191.7, 156.5, 152.8, 132.9, 125.5, 121.0, 119.1, 117.7, 108.6, 69.2, 67.0, 37.6; HRMS, found 205.0860 (C12H13O3, $[M + H]^+$, requires 205.0859) *apparent dq, hypothesised to be an overlapping dtd, with an almost identical J-coupling constant for the geminal and ^{4}J couplings.

Compound S14 - 6-(allyloxy)-4-(aminomethyl)chroman-4-ol

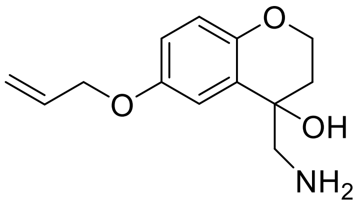

A solution of **S13** (640 mg, 3.0 mmol) and ZnI_2 (19 mg, 0.06 mmol) were suspended in DCM and cooled to 0 °C. Trimethylsilyl cyanide (565 μL, 4.5 mmol) was added dropwise and the resulting solution allowed to stir overnight and concentrated *in vacuo.* A suspension of $LiAlH_4$ (455 mg, 12 mmol) in THF was cooled to 0 °C and the crude cyanohydrin was dissolved in THF and added dropwise over 15 min. The reaction was allowed to stir at 0 °C until completion. The reaction was quenched using Fieser procedure and concentrated *in vacuo* and purified by flash column chromatography to afford the title compound, as a brown oil (630 mg, 70%).

^{1}H NMR (400 MHz, Methanol-d_4) δ 7.02 (d, J = 3.0 Hz, 1H, ArC**H**), 6.79 (dd, J = 8.9, 2.9 Hz, 1H, ArC**H**), 6.72 (d, J = 8.9 Hz, 1H, ArC**H**), 6.11 – 6.00 (m, 1H, $OCH_2C\mathbf{H}CH_2$), 5.40 (app-dq, J = 17.3, 1.7 Hz, 1H, $OCH_2CHC\mathbf{H_2}$), 5.25 (app-dq, J = 10.5, 1.5 Hz, 1H, $OCH_2CHC\mathbf{H_2}$), 4.50 (m, 2H, $OC\mathbf{H_2}CHCH_2$), 4.23 – 4.13 (m, 2H, $OC\mathbf{H_2}CH_2$), 2.98 – 2.86 (m, 2H, $COHC\mathbf{H_2}NH_2$), 2.25 – 2.17 (m, 1H, $OCH_2C\mathbf{H_2}$), 2.00 – 1.92 (m, 1H, $OCH_2C\mathbf{H_2}$); ^{13}C NMR (101 MHz, Methanol−d_4) *d* 153.9, 150.3, 135.2, 128.4, 118.5, 117.4, 117.4, 113.5, 70.4, 69.6, 64.2, 51.8, 33.8; HRMS, found 236.1287 ($C_{13}H_{18}NO_3$, $[M + H]^+$, requires 236.1282). *apparent dq, hypothesised to be an overlapping dtd, with an almost identical J-coupling constant for the geminal and ^{4}J couplings.

Disease Models & Mechanisms • Supplementary information

Compound 5 - N-((6-(allyloxy)-4-hydroxychroman-4-yl)methyl)-3-Bromobenzenesulfonamide

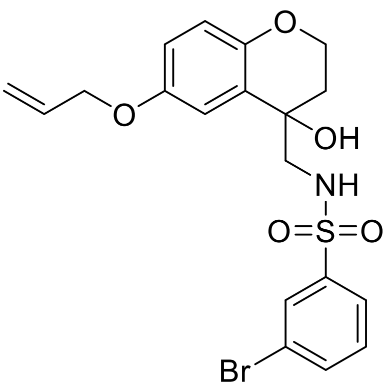

Compound **14** (540 mg, 2.3 mmol) was dissolved in DCM under an inert atmosphere at 0°C, before triethylamine (TEA) (800 µL, 5.75 mmol) was added dropwise. A solution of 3-bromobenzenesulfonyl chloride (980 mg, 3.4 mmol) in DCM was then added over 10 min. The reaction was stirred at RT until completion. The resulting solution was diluted DCM (20 mL), neutralised, washed with H_2O (3 × 20 mL), extracted with DCM (3 × 60 mL), washed with brine (100 mL), dried over $MgSO_4$ and concentrated in vacuo. The crude residue was purified by column chromatography (33% EtOAc in Hexane) to afford the title compound, as a brown oil (558 mg, 53%).

^{1}H NMR (400 MHz, MSO-d$_6$) δ 7.97 (t, J = 1.8 Hz, 1H, ArC**H**), 7.89 (s, 1H, SO$_2$N**H**), 7.86 – 7.79 (m, 2H, ArC**H**), 7.54 (t, J = 7.9 Hz, 1H, ArC**H**), 6.92 (d, J = 3.0 Hz, 1H, ArC**H**), 6.76 (dd, J = 8.9, 3.0 Hz, 1H, ArC**H**), 6.67 (d, J = 8.9 Hz, 1H, ArC**H**), 6.06 – 5.96 (m, 1H, OCH$_2$C**H**CH$_2$), 5.40 (s, 1H, CO**H**), 5.39 – 5.33 (m, 1H, OCH$_2$CHC**H$_2$**), 5.24 – 5.20 (m, J = 10.5, 1.5 Hz, 1H, OCH$_2$CHC**H$_2$**), 4.49 – 4.42 (m, 2H, OC**H$_2$**CHCH$_2$), 4.15 – 4.05 (m, 2H, OC**H**$_2$CH$_2$), 3.14 (d, J = 13.3 Hz, 1H, COHC**H$_2$**NH), 2.97 (d, J = 13.3 Hz, 1H, COHC**H$_2$**NH), 2.23 – 2.16 (m, 1H, OCH$_2$C**H$_2$**), 1.86 – 1.79 (m, 1H, OCH$_2$C**H$_2$**); ^{13}C NMR (101 MHz, DMSO-d$_6$) δ 161.2, 157.7, 152.4, 144.6, 143.5, 140.8, 138.4, 136.8, 135.0, 131.5, 126.5, 126.3, 125.3, 122.4, 78.1, 76.6, 72.1, 60.6, 41.9; HRMS, found 454.0161 ($C_{19}H_{19}NO_5S_{79}Br$, [M - H]$^-$, requires 452.0167).

Compound S15 - 3-bromo-N-((4,6-dihydroxychroman-4-yl)methyl)benzenesulfonamide

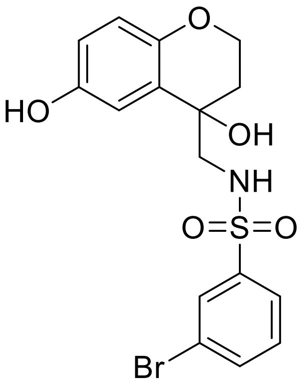

Compound **5** (530 mg, 1.2 mmol) was dissolved in MeOH (20 mL), before Pd[PPh$_3$]$_4$ (135 mg, 0.3 mmol) was added and allowed to stir for 10 min. K_2CO_3 (481 mg, 3.5 mmol) was added and the reaction was allowed to reflux, at 60°C), ON. The resulting solution was concentrated in vacuo, taken up in DCM (20 mL), washed with H_2O (3 × 20 mL), extracted with DCM (2 × 60 mL), washed with brine (100 mL), dried over $MgSO_4$ and concentrated in vacuo. The crude residue was purified by column chromatography (50% EtOAc in Hexane) to afford the title compound, as a white foam (356 mg, 75%).

^{1}H NMR (400 MHz, DMSO-d$_6$) *δ* 8.86 (s, 1H, ArCO**H**), 7.99 (t, J = 1.8 Hz, 1H, ArC**H**), 7.88 (s, 1H, SO$_2$N**H**), 7.87 – 7.83 (m, 1H, ArC**H**), 7.83 – 7.80 (m, 1H, ArC**H**), 7.55 (t, J = 7.9 Hz, 1H, ArC**H**), 6.73 (t, J = 1.6 Hz, 1H, ArC**H**), 6.56 (d, J = 1.6 Hz, 2H, ArC**H**), 5.35 (s, 1H, CO**H**CH$_2$NH), 4.14 – 3.99 (m,

Disease Models & Mechanisms • Supplementary information

2H, OC**H**$_2$CH$_2$), 3.06 (dd, J = 13.1, 7.4 Hz, 1H, COHC**H**$_2$NH), 2.90 (dd, J = 13.2, 4.9 Hz, 1H, COHC**H**$_2$NH), 2.23 – 2.12 (m, 1H, OCH$_2$C**H**$_2$), 1.88 – 1.77 (m, 1H, OCH$_2$C**H**$_2$); ^{13}C NMR (101 MHz, DMSO-d$_6$) δ 150.6, 146.9, 142.8, 135.2, 131.4, 129.0, 127.4, 125.6 , 122.1, 116.8, 116.1, 113.1, 67.1, 62.7, 51.3, 32.5; HRMS, found 411.9860 ($C_{16}H_{15}NO_5S_{79}Br$, [M - H]$^-$, requires 411.9854).

Compound 17 - 4-(hept-6-yn-1-yloxy)benzenesulfonyl chloride

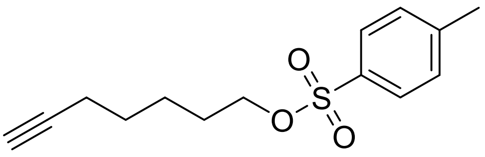

6-Heptyn-1-ol **S16** (670 μL, 5.4 mmol) was dissolved in DCM (40 mL) and cooled to 0°C. Freshly recrystallised para-toluenesulfonyl chloride (1.5 g, 6.5 mmol), DMAP (65 mg, 0.5 mmol) and TEA (875 μL, 3.0 mmol) were added sequentially and allowed to stir for 30 min. The resulting solution was washed with NH_4Cl (3 × 40 mL), extracted EtOAc (2 × 100 mL), dried over $MgSO_4$ and concentrated in vacuo. The crude residue was purified by column chromatography (50% EtOAc in Hexane) to afford the title compound, as a clear viscous solution (1.0 g, 70%).

^{1}H NMR (400 MHz, $CDCl_3$) δ 7.81 (d, J = 1.9 Hz, 1H, ArC**H**), 7.79 (d, J = 2.0 Hz, 1H, ArC**H**), 7.37 (d, J = 0.9 Hz, 1H, ArC**H**), 7.35 (d, J = 0.8 Hz, 1H, ArC**H**), 4.04 (t, J = 6.5 Hz, 2H, OC**H$_2$**CH$_2$), 2.46 (s, 3H, ArCC**H$_3$**), 2.16 (*app-td, J = 6.7, 2.6 Hz, 2H, OCH$_2$C**H$_2$**), 1.94 (t, J = 2.7 Hz, 1H, C≡C**H**), 1.72 – 1.64 (m, 2H, C**H$_2$**C≡CH), 1.53 – 1.39 (m, 4H, C**H$_2$**C**H$_2$**CH$_2$C≡CH); ^{13}C NMR (101 MHz, $CDCl_3$) δ 144.8, 133.3, 129.9, 128.0, 84.1, 70.4, 68.6, 28.5, 27.8, 24.6, 21.7, 18.3. *app-td, hypothesised to be a tt, unresolved due to similarities in J-coupling constants.

Compound 10 - 3-bromo-N-((4,6-dihydroxychroman-4-yl)methyl)-N-(hept- 6-yn-1-yl)benzenesulfonamide and Compound 6 - 3-bromo-N-((6-(hept-6- yn-1-yloxy)-4-hydroxychroman-4-yl)methyl)benzenesulfonamide

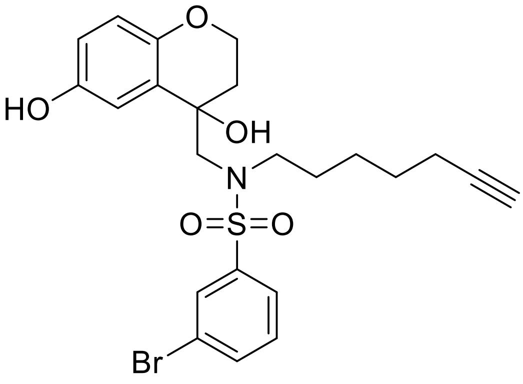

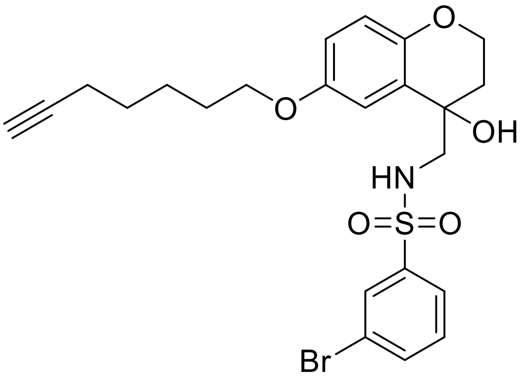

Compound **S15** (24 mg, 0.090 mmol), **S17** (40 mg, 0.10 mmol) and K_2CO_3 (30 mg, 0.21 mmol) were prepared in DMF (2 mL) and allowed to stir at 50°C for 6 h. The resulting solution was diluted in EtOAc, washed with minimal H_2O, extracted with EtOAc (20 mL), washed with brine (3 × 5 mL), dried over $MgSO_4$ and concentrated in vacuo. Compounds **56** and **57** were purified using PREP-

Disease Models & Mechanisms • Supplementary information

LCMS 30-minute method, 50 to 98% ACN in H_2O. Compounds **56** (Rt 6.9 min, ES− m/z $(M - H)^-$ = 508) and **57** (Rt 8 min, ES^- m/z $(M - H)^-$ = 508) to afford the title compounds, as two pale brown oils.

Compound **10**; ^{1}H NMR (400 MHz, DMSO-d_6) δ 8.94 (s, 1H, ArO**H**), 7.96 (t, J = 1.8 Hz, 1H, ArC**H**), 7.91 – 7.88 (m, 1H, ArC**H**), 7.84 – 7.80 (m, 1H, ArC**H**), 7.55 (t, J = 8.0 Hz, 1H, ArC**H**), 6.83 (t, J = 1.7 Hz, 1H, ArC**H**), 6.59 (d, J = 1.6 Hz, 2H, ArC**H**), 5.39 (s, 1H, CO**H**CH_2N), 4.22 – 4.06 (m, 2H, O$C\mathbf{H_2}CH_2$), 3.61 (d, J = 14.5 Hz, 1H, N$C\mathbf{H_2}CH_2$), 3.35 – 3.25 (m, 1H, COHC$\mathbf{H_2}$N), 3.21 (d, J = 14.5 Hz, 1H, N$C\mathbf{H_2}CH_2$), 3.17 – 3.08 (m, 1H, COHC$\mathbf{H_2}$N), 2.74 (t, J = 2.6 Hz, 1H, C≡C**H**), 2.33 – 2.22 (m, 1H, O$CH_2C\mathbf{H_2}$), 2.08 (app-td, J = 7.0, 2.6 Hz, 2H, C$\mathbf{H_2}$C≡CH), 1.98 – 1.86 (m, 1H, O$CH_2C\mathbf{H_2}$), 1.53 – 1.42 (m, J = 7.6 Hz, 2H, N$CH_2C\mathbf{H_2}CH_2$), 1.39 – 1.30 (m, 2H, N$CH_2CH_2C\mathbf{H_2}$), 1.19 – 1.09 (m, 2H, C$\mathbf{H_2}CH_2$C≡CH); ^{13}C NMR (101 MHz, DMSO-d_6) *δ* 150.8, 146.6, 141.4, 135.7, 131.7, 129.2, 128.1, 126.1, 122.4*,* 116.8, 116.1, 113.2, 84.4, 71.3, 68.4, 62.8, 56.3, 50.0, 32.5, 27.6, 27.2, 25.4, 17.6. *app-td, hypothesised to be a tt, unresolved due to similarities in J-coupling constants.

Compound **6**; ^{1}H NMR (400 MHz, DMSO-d_6) δ 7.79 (s, 1H, SO_2N**H**), 7.75 (dt, J = 7.7, 1.6 Hz, 1H, ArC**H**), 7.65 – 7.58 (m, 1H, ArC**H**), 7.55 (t, J = 7.7 Hz, 1H, ArC**H**), 7.34 (dd, J = 7.7, 1.7 Hz, 1H, ArC**H**), 7.19 – 7.09 (m, 1H, ArC**H**), 6.86 (td, J = 7.5, 1.2 Hz, 1H, ArC**H**), 6.74 (dd, J = 8.2, 1.2 Hz, 1H, ArC**H**), 5.38 (s, 1H, CO**H**CH_2NH), 4.40 (s, 1H, C≡C**H**), 4.24 – 4.09 (m, 2H, O$C\mathbf{H_2}CH_2$), 3.46 – 3.38 (m, 2H, O$C\mathbf{H_2}CH_2$), 3.11 (dd, J = 13.2, 6.7 Hz, 1H, COHC$\mathbf{H_2}$NH), 2.94 (dd, J = 13.2, 3.9 Hz, 1H, COHC$\mathbf{H_2}$NH), 2.46 (t, J = 7.0 Hz, 2H, C$\mathbf{H_2}$C≡CH), 2.28 – 2.17 (m, 1H, O$CH_2C\mathbf{H_2}$), 1.92 – 1.81 (m, 1H, O$CH_2C\mathbf{H_2}$), 1.61 – 1.51 (m, 2H, O$CH_2C\mathbf{H_2}$), 1.51 – 1.39 (m, 4H, O$CH_2CH_2C\mathbf{H_2}C\mathbf{H_2}$); ^{13}C NMR (101 MHz, DMSO-d_6) δ 154.1, 141.2, 134.7, 131.4, 129.5, 129.0, 128.8, 128.7, 127.5, 126.9, 125.8, 124.2, 120.0, 116.2, 92.8, 79.3, 66.8, 62.8, 60.6, 51.1, 32.3, 27.9, 25.0, 18.7; HRMS, found 506.0645 ($C_{23}H_{25}NO_5S_{79}Br$, $[M - H]^-$, requires 506.0637).

Disease Models & Mechanisms • Supplementary information

SYNTHESIS OF COMPOUND 7

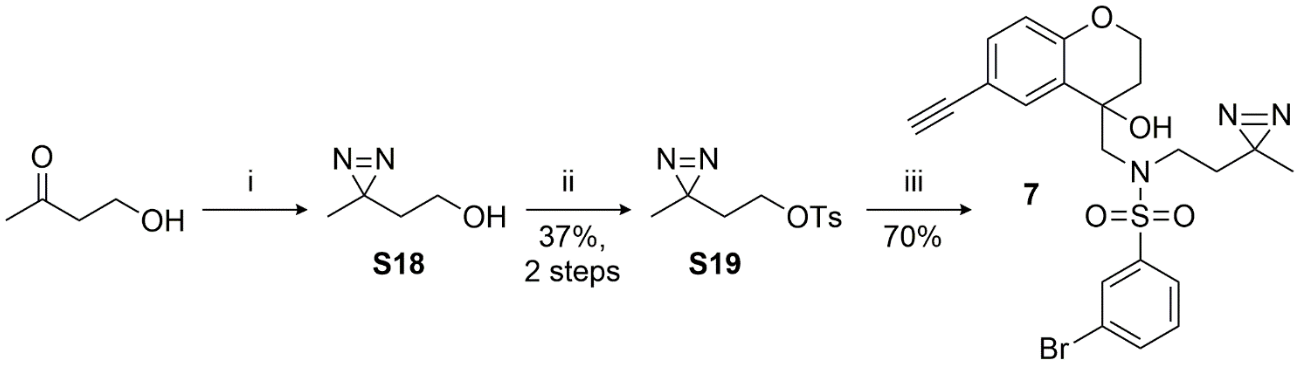

SCHEME 6: Synthesis of sulfonamide linked/diazirine containing photo-affinity probe. i - NH_3(l), HOSA, -78°C, ON. Followed by I_2, DCM, RT, 3 h. **ii** - p-TsCl, DMAP, TEA, DCM,RT, 30 min. **iii** - Compound 35, K_2CO_3, DMF, RT, ON.

Compound S18 - 2-(3-methyl-3H-diazirin-3-yl)ethan-1-ol

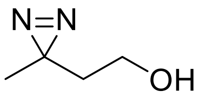

Ammonia (30 mL) was condensed into a 3-neck RBF at -78°C, under argon. 4-hydroxy-2-butanone (1.0 mL, 11 mmol) was added and allowed to stir at -78°C for 5 h. Hydroxylamine-O-sulfonic acid (1.7 g, 15 mmol) was dissolved in dry MeOH (10 mL) and added over 30 min. The reaction was allowed to stir overnight and allowed to warm to RT. The resulting slurry was filtered, washed with DCM (2 × 15 mL); before TEA (7 mL, 50 mmol) was added, followed by iodine portion-wise until a red/brown colour persisted. The reaction was washed with 1M HCl (3 × 30 mL), 10% sodium thiosulphate (3 × 30 mL) and brine (2 × 30 mL), dried over $MgSO_4$ and concentrated in vacuo to afford the crude title compound, as a yellow solid (~900 mg), in residual TEA. Due to the volatile nature of the product and necessity for base in the next reaction, the product was taken forward without further purification.

^{1}H NMR (400 MHz, Methanol) δ 3.77 – 3.66 (m, 2H, C**H**$_2$OH), 1.86 – 1.81 (m, 2H, C**H**$_2$CH$_2$OH), 1.40 (s, 3H, C**H**$_3$); ^{13}C NMR (101 MHz, Methanol) δ 59.2 (**C**H$_2$OH), 55.1 (**C**NHNH), 40.9 (**C**H$_2$CH$_2$OH), 23.1 (**C**H$_3$).

Compound S19 - 2-(3-methyl-3H-diazirin-3-yl)ethyl-4-methylbenzenesulfonate

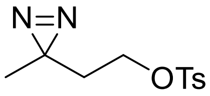

Compound **18** (200 mg, 2.0 mmol) was dissolved in anhydrous DCM (5 mL) and cooled to 0°C. Para-toluenesulfonyl chloride (455 mg, 2.4 mmol), TEA (300 μL, 2.4 mmol) and DMAP (25 mg, 0.2 mmol) were added. The reaction was warmed to RT and allowed to stir for 30 min, before being quenched with NH_4Cl (20 mL), extracted with EtOAc (3 × 20 mL), washed with brine (2 × 70 mL), dried over $MgSO_4$ and concentrated in vacuo. The crude residue was purified by column chromatography (5% EtOAc in Hexane) to afford the title compound, as a colourless oil (186 mg, 37%).

^{1}H NMR (400 MHz, $CDCl_3$) δ 7.85 – 7.77 (m, 2H, ArC**H**), 7.40 – 7.32 (m, 2H, ArC**H**), 3.94 (t, J = 6.4 Hz, 2H, $SO_3C\mathbf{H_2}$), 2.45 (s, 3H, ArCC**H₃**), 1.66 (t, J = 6.4 Hz, 2H, $SO_3CH_2C\mathbf{H_2}$), 0.99 (s, 3H, $CN_2C\mathbf{H_3}$); ^{13}C NMR (101 MHz, $CDCl_3$) δ 145.2, 132.8, 130.0, 128.1, 65.2, 34.3, 23.5, 21.8, 19.9; HRMS, found 333.0947 ($C_{13}H_{21}N_2O_4S_2$, $[M + H + DMSO]^+$, requires 333.0943).

Compound 7 - 3-bromo-N-((6-ethynyl-4-hydroxychroman-4-yl)methyl)-N-(2-(3-methyl-3H-diazirin-3-yl)ethyl)benzenesulfonamide

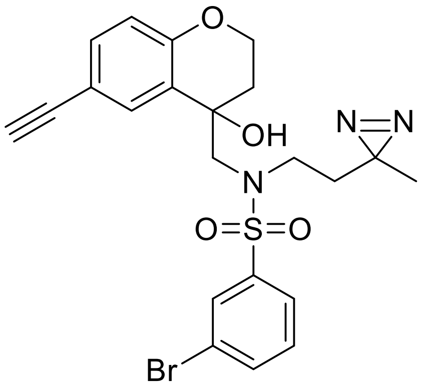

Compound **3** (85 mg, 0.20 mmol) and **S19** (100 mg, 0.38 mmol) were dissolved in DMF (4 mL); K_2CO_3 (95 mg, 0.69 mmol) was added, the reaction warmed to 50°C and allowed to stir overnight. The resulting solution was diluted in EtOAc (30 mL), washed with minimal H_2O (3 × 5 mL), extracted with EtOAc (3 × 20 mL), washed with brine (2 × 60 mL), dried over $MgSO_4$ and concentrated in vacuo. The crude residue was purified by column chromatography (5 to 15% EtOAc in Hexane) to afford the title compound, as a white foam (71 mg, 70%).

^{1}H NMR (400 MHz, $CDCl_3$) δ 7.88 (t, J = 1.8 Hz, 1H, ArC**H**), 7.74 – 7.70 (m, 1H, ArC**H**), 7.68 – 7.65 (m, 1H, ArC**H**), 7.52 (d, J = 2.1 Hz, 1H, ArC**H**), 7.40 (t, J = 7.9 Hz, 1H, ArC**H**), 7.32 (dd, J = 8.5, 2.1 Hz, 1H, ArC**H**), 6.78 (d, J = 8.5 Hz, 1H, ArC**H**), 4.38 – 4.26 (m, 2H, $OC\mathbf{H_2}CH_2$), 3.71 (d, J = 14.6 Hz, 1H, $COHC\mathbf{H_2}N$), 3.38 – 3.29 (m, 1H, $C\mathbf{H_2}CH_2$), 3.20 – 3.11 (m, 2H, $NC\mathbf{H_2}CH_2$ and $COHC\mathbf{H_2}N$), 2.99 (s, 1H, C≡C**H**), 2.60 – 2.52 (m, 1H, $CH_2C\mathbf{H_2}$), 2.06 – 1.94 (m, 1H, $OCH_2C\mathbf{H_2}$), 1.80 – 1.61 (m, 2H, $NCH_2C\mathbf{H_2}$), 0.98 (s, 3H, $CN_2C\mathbf{H_3}$); ^{13}C NMR (101 MHz, $CDCl_3$) δ 155.0, 140.7, 136.2, 133.8, 131.0, 130.9,130.1, 126.5, 125.8, 123.6, 117.7, 114.5, 83.4, 76.3, 69.9, 64.1, 57.3, 46.6, 34.5, 32.7, 24.1, 19.7; HRMS, found 504.0590 ($C_{22}H_{23}N_3O_4S_{79}Br$, $[M + H]^+$, requires 504.0593). Found 582.0669 ($C_{24}H_{29}N_3O_5S_{279}Br$, $[M + DMSO + H]^+$, requires 582.1580).

Disease Models & Mechanisms • Supplementary information

COMPOUND 10

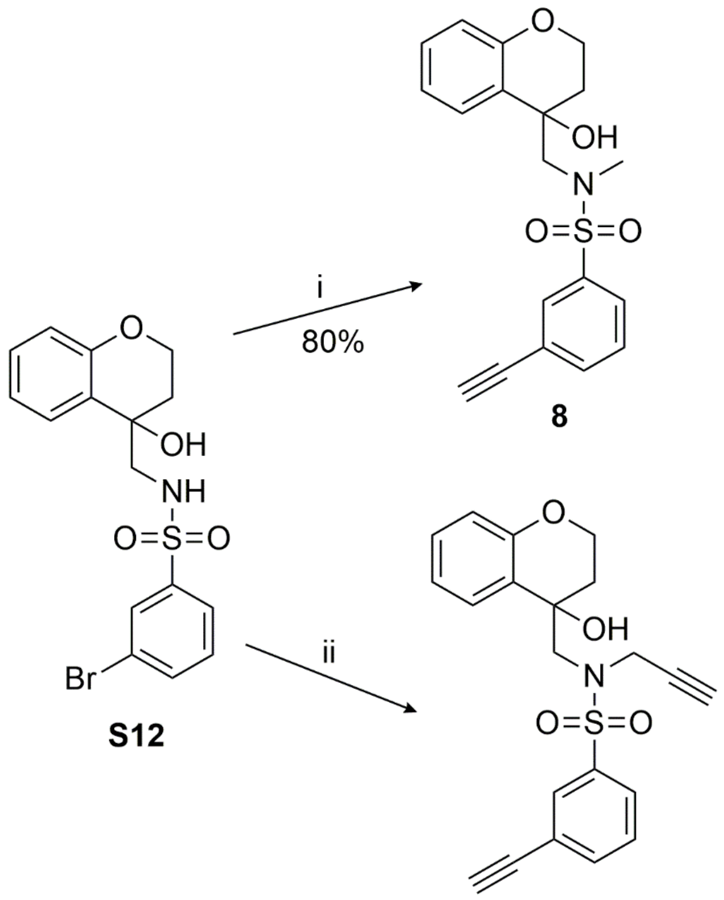

SCHEME 7: Synthesis of 10. i – MeI, K_2CO_3, DMF, 50°C, 4 h. **ii -** propargyl bromide, K_2CO_3, DMF, 50°C, 4 h.

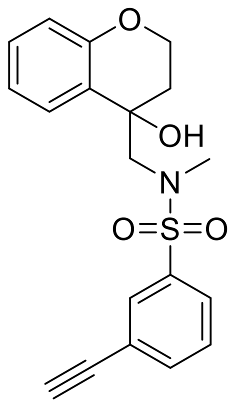

Compound 8 - 3-bromo-N-((4-hydroxychroman-4-yl)methyl)-Nmethylbenzenesulfonamide

Compound **S12** (40 mg, 0.10 mmol), synthesised as previously described, and K_2CO_3 (48 mg, 0.35 mmol) were suspended in DMF (1.5 mL), before methyl iodide (14 μL, 0.20 mmol) was added dropwise and the reaction heated to 50°C for 4 h. The resulting solution was diluted in EtOAc (10 mL), washed with minimal H_2O (3 × 3 mL), extracted with EtOAc (3 × 10 mL), washed with brine (30 mL), dried over $MgSO_4$ and concentrated in vacuo. The crude residue was purified by column chromatography (25% EtOAc in Hexane) to afford the title compound, as a pale brown oil (36 mg, 80%).

^{1}H NMR (400 MHz, $CDCl_3$) δ 7.90 (t, J = 1.8 Hz, 1H, ArC**H**), 7.72 (ddd, J = 8.0, 1.9, 1.0 Hz, 1H, ArC**H**), 7.68 (ddd, J = 7.9, 1.7, 1.0 Hz, 1H, ArC**H**), 7.40 (t, 1H, ArC**H**), 7.37 (dd, 1H, ArC**H**), 7.20

Disease Models & Mechanisms • Supplementary information

(ddd, J = 8.2, 7.3, 1.7 Hz, 1H, ArC**H**), 6.91 (ddd, J = 7.7, 7.2, 1.2 Hz, 1H, ArC**H**), 6.84 (dd, J = 8.2, 1.2 Hz, 1H, ArC**H**), 4.39 – 4.24 (m, 2H, OC**H$_2$**CH$_2$), 3.62 (d, J = 14.4 Hz, 1H, COHC**H$_2$**N), 3.11 (d, J = 14.4 Hz, 1H, COHC**H$_2$**N), 2.94 (s, 3H, NC**H$_3$**), 2.62 – 2.52 (m, 1H, OCH$_2$C**H$_2$**), 2.33 (s, 1H, CO**H**CH$_2$NH), 2.14 – 2.05 (m, 1H, OCH$_2$C**H$_2$**); ^{13}C NMR (101 MHz, CDCl$_3$) δ 154.4, 139.3, 136.0, 130.8, 130.3, 129.9, 126.7, 126.2, 126.0, 123.4, 120.9, 117.3 , 70.0, 63.8, 58.6, 38.3, 33.2; HRMS, found 456.0117 ($C_{18}H_{19}NO_6S_{79}Br$, [M – OH + HCO_2H]$^+$, requires 456.0116).

Compound 9 - 3-bromo-N-((4-hydroxychroman-4-yl)methyl)-N-(prop-2-yn-1-yl)benzenesulfonamide

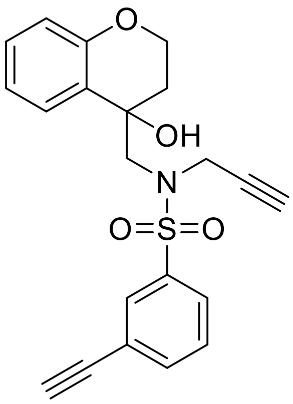

Compound **S12** (40 mg, 0.10 mmol), synthesised as previously described, and K_2CO_3 (48 mg, 0.30 mmol) were suspended in DMF (1.5 mL), before propargyl bromide (24 *μ*L, 0.20 mmol) was added dropwise and solution heated to 50°C for 4 h. The resulting solution was diluted in EtOAc (10 mL), washed with minimal H_2O (3 × 3 mL), extracted with EtOAc (3 × 10 mL, washed with brine (30 mL), dried over $MgSO_4$ and concentrated in vacuo. The crude residue was purified by column chromatography (25% EtOAc in Hexane) to afford the title compound, as a pale brown oil (38 mg, 87%).

^{1}H NMR (400 MHz, CDCl3) *δ* 8.01 (t, J = 1.8 Hz, 1H, ArC**H**), 7.78 – 7.70 (m, 2H, ArC**H**), 7.46 (dd, J = 7.8, 1.7 Hz, 1H, ArC**H**), 7.38 (t, J = 7.9 Hz, 1H, ArC**H**), 7.26 – 7.21 (m, 1H, ArC**H**), 6.95 (td, J = 7.5, 1.2 Hz, 1H, ArC**H**), 6.87 (dd, J = 8.3, 1.2 Hz, 1H, ArC**H**), 4.53 (dd, J = 18.6, 2.5 Hz, 1H, NC**H$_2$**C≡CH), 4.39 – 4.28 (m, 3H, NC**H$_2$**C≡CH mixed with OC**H$_2$**CH$_2$), 3.65 (d, J = 14.8 Hz, 1H, COHC**H$_2$**N), 3.55 (d, J = 14.8 Hz, 1H, COHC**H$_2$**N), 2.63 – 2.56 (m, 1H, OCH$_2$C**H$_2$**), 2.36 (s, 1H, CO**H**CH$_2$N), 2.15 – 2.09 (m, 1H, OCH$_2$C**H$_2$**), 2.08 (t, J = 2.5 Hz, 1H, CH2C≡C**H**); ^{13}C NMR (101 MHz, CDCl$_3$) δ 154.3, 140.5, 136.0, 130.8, 130.4, 129.9, 126.6, 126.4, 126.0, 122.9, 120.8, 117.3, 76.7*,* 74.5, 70.1, 63.7, 53.7, 39.2, 33.1; HRMS, found 418.0109 ($C_{19}H_{17}NO_3S_{79}Br$, [M - OH]$^+$, requires 418.0113).

CLICKED PROBES 11 AND 12

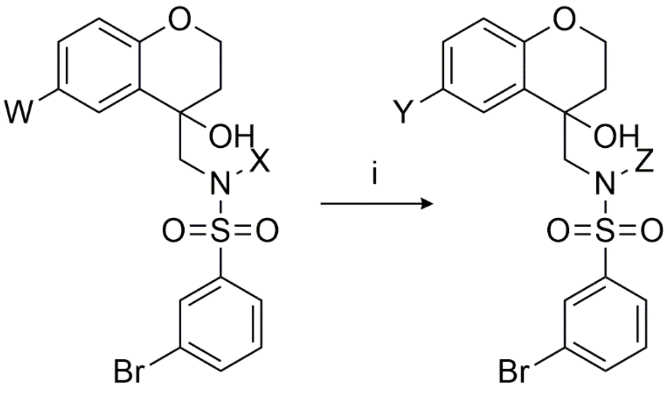

SCHEME 7: Synthesis of clicked probes 11 and 12. W = H or Ethyne, X = H or propyne, Y = H or 1-butyl-1H-1,2,3-triazole, Z = H or 1-butyl-1H-1,2,3-triazole. **i** - BuBr, NaN_3, $CuBr(PPh_3)_3$, H_2O/Acetone (1:1), RT, ON.

Compound 11 - 3-bromo-N-((6-(1-butyl-1H-1,2,3-triazol-4-yl)-4-hydroxychroman-4-yl)methyl)benzenesulfonamide

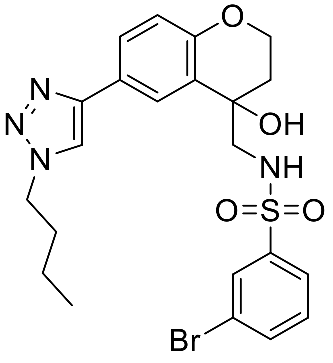

Compound **3** (10 mg, 0.02 mmol), 1-bromobutane (0.5 µL, 0.003 mmol), NaN_3 (0.3 mg, 0.003 mmol) and $CuBr(PPh_3)_3$ (0.1 mg, 5 mol%) were dissolved in H_2O/Acetone (0.2 mL, 1:1) and HCl (0.1 mL). The reaction was heated and allowed to stir at 50°C for 72 h. The reaction was diluted in H_2O (10 mL), extracted in EtOAc (3 × 10 mL), washed with brine (2 × 30 mL), dried over $MgSO_4$ and concentrated *in vacuo*. The crude residue was purified by column chromatography (10% EtOAc in Hexane) to afford the title compound, as a white foam (7 mg, 65%).

^{1}H NMR (400 MHz, DMSO-d_6) δ 8.40 (s, 1H, ArC**H**), 7.97 (t, J = 1.8 Hz, 1H, ArC**H**), 7.96 – 7.90 (m, 1H, ArC**H**), 7.85 – 7.78 (m, 3H, ArC**H** and COHCH$_2$N**H**), 7.59 (dd, J = 8.4, 2.1 Hz, 1H, ArC**H**), 7.51 (t, J = 7.9 Hz, 1H, ArC**H**), 6.81 (d, J = 8.4 Hz, 1H, ArCC=C**H**), 5.46 (s, 1H, CO**H**CH$_2$N), 4.36 (t, J = 7.0 Hz, 2H, NC**H$_2$**CH$_2$), 4.26 – 4.13 (m, 2H, OC**H$_2$**CH$_2$), 3.21 (dd, J = 13.3, 7.6 Hz, 1H, COHC**H$_2$**NH), 3.04 (dd, J = 13.3, 5.5 Hz, 1H, COHC**H$_2$**NH), 2.31 – 2.21 (m, 1H, OCH$_2$C**H$_2$**), 1.94 – 1.87 (m, 1H, OCH$_2$C**H$_2$**), 1.87 – 1.77 (m, 2H, NCH$_2$C**H$_2$**), 1.32 – 1.20 (m, 2H, CH$_2$C**H$_2$**CH$_3$), 0.90 (t, J = 7.3 Hz, 3H, CH$_2$CH$_2$C**H$_3$**); ^{13}C NMR (101 MHz, DMSO-d_6) δ 153.8, 146.3, 142.9, 135.0, 131.3, 128.9, 127.2, 125.7, 125.4, 124.4, 123.0, 122.0, 120.1, 116.7, 66.9, 62.9, 51.0, 49.1, 32.1, 31.6, 19.0, 13.2; HRMS, found 521.0855 ($C_{22}H_{26}N_4O_4S_{79}Br$, $[M + H]^+$, requires 521.0858).

Compound 12 - 3-bromo-N-((1-butyl-1H-1,2,3-triazol-4-yl)methyl)-N-((4- hydroxychroman-4-yl)methyl)benzenesulfonamide

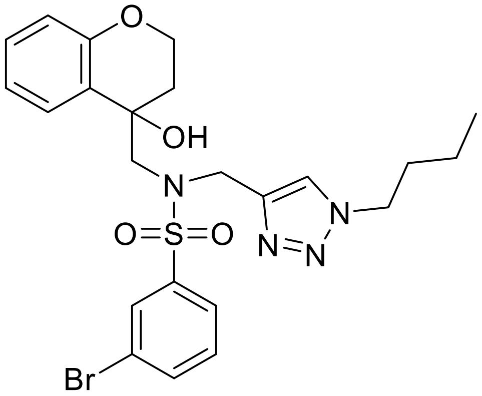

Compound **9** (60 mg, 0.90 mmol), 1-bromobutane (20 µL, 0.14 mmol), NaN_3 (12 mg, 0.012 mmol) and $CuBr(PPh_3)_3$ (5 mg, 5 mol%) were dissolved in H_2O/Acetone (1 mL, 1:1) and HCl (1.6 mL). The reaction was heated and allowed to stir at 50°C for 72 h. The reaction was diluted in H_2O (10 mL), extracted in EtOAc (3 × 10 mL), washed with brine (2 × 30 mL), dried over $MgSO_4$ and concentrated *in vacuo*. The crude residue was purified by column chromatography (50% EtOAc in Hexane) to afford the title compound, as a white foam (29 mg, 60%).

^{1}H NMR (400 MHz, DMSO-d_6) δ 7.83 (s, 1H, ArC**H**), 7.78 – 7.76 (m, 1H, ArC**H**), 7.76 – 7.73 (m, 1H, ArC**H**), 7.70 – 7.67 (m, 1H, ArC**H**), 7.49 (dd, J = 7.8, 1.6 Hz, 1H, ArC**H**), 7.37 (t, J = 7.9 Hz, 1H, ArC**H**), 7.19 – 7.14 (m, 1H, ArC**H**), 6.93 – 6.88 (m, 1H, ArC**H**), 6.79 – 6.76 (m, 1H, $NCH_2C=CH$), 5.61 (s, 1H, CO**H**CH_2N), 4.94 (d, J = 16.1 Hz, 1H, NC**H₂**C=C), 4.71 (d, J = 16.1 Hz, 1H, NC**H₂**C=C), 4.34 – 4.26 (m, 2H, O**CH₂**CH_2), 4.26 – 4.22 (m, 2H, N**CH₂**CH_2), 3.72 (d, J = 14.7 Hz, 1H, COHC**H₂**N), 3.57 (d, J = 14.7 Hz, 1H, COHC**H₂**N), 2.45 – 2.38 (m, 1H, OCH_2C**H₂**), 2.10 – 2.01 (m, 1H, OCH_2C**H₂**), 1.73 – 1.64 (m, 2H, NCH_2C**H₂**), 1.21 – 1.11 (m, 2H, C**H₂**CH_3), 0.88 (t, J = 7.3 Hz, 3H, CH_2C**H₃**); ^{13}C NMR (101 MHz, DMSO-d_6) δ 153.8, 141.6, 141.3, 135.3, 131.0, 129.3, 128.8, 127.7, 127.7, 126.2, 123.8, 121.8, 120.1, 116.2, 69.0, 63.2, 54.4, 48.9, 43.5, 32.2, 31.6, 19.0, 13.3; HRMS, found 535.0994 ($C_{23}H_{28}N_4O_4S_{79}Br$, [M + H]+, requires 535.1015).

Disease Models & Mechanisms • Supplementary information
